# Supplementary material for: Rational Design of DNA-Expressed Stabilized Native-Like HIV-1 Envelope Trimers
Source: Cell Rep. 2018 Sep 18;24(12):3324–3338.e5. doi: 10.1016/j.celrep.2018.08.051 (PMC6167709; doi:10.1016/j.celrep.2018.08.051)
Supplement: Document S1. Supplemental Data and Figures S1–S7 [file mmc1.pdf]

Cell Reports, Volume *24*

Supplemental Information

Rational Design of DNA-Expressed

Stabilized Native-Like HIV-1 Envelope Trimers

Yoann Aldon, Paul F. McKay, Joel Allen, Gabriel Ozorowski, Réka Felfödiné Lévai, Monica Tolazzi, Paul Rogers, Linling He, Natalia de Val, Katalin Fábián, Gabriella Scarlatti, Jiang Zhu, Andrew B. Ward, Max Crispin, and Robin J. Shattock

SUPPLEMENTAL FIGURES

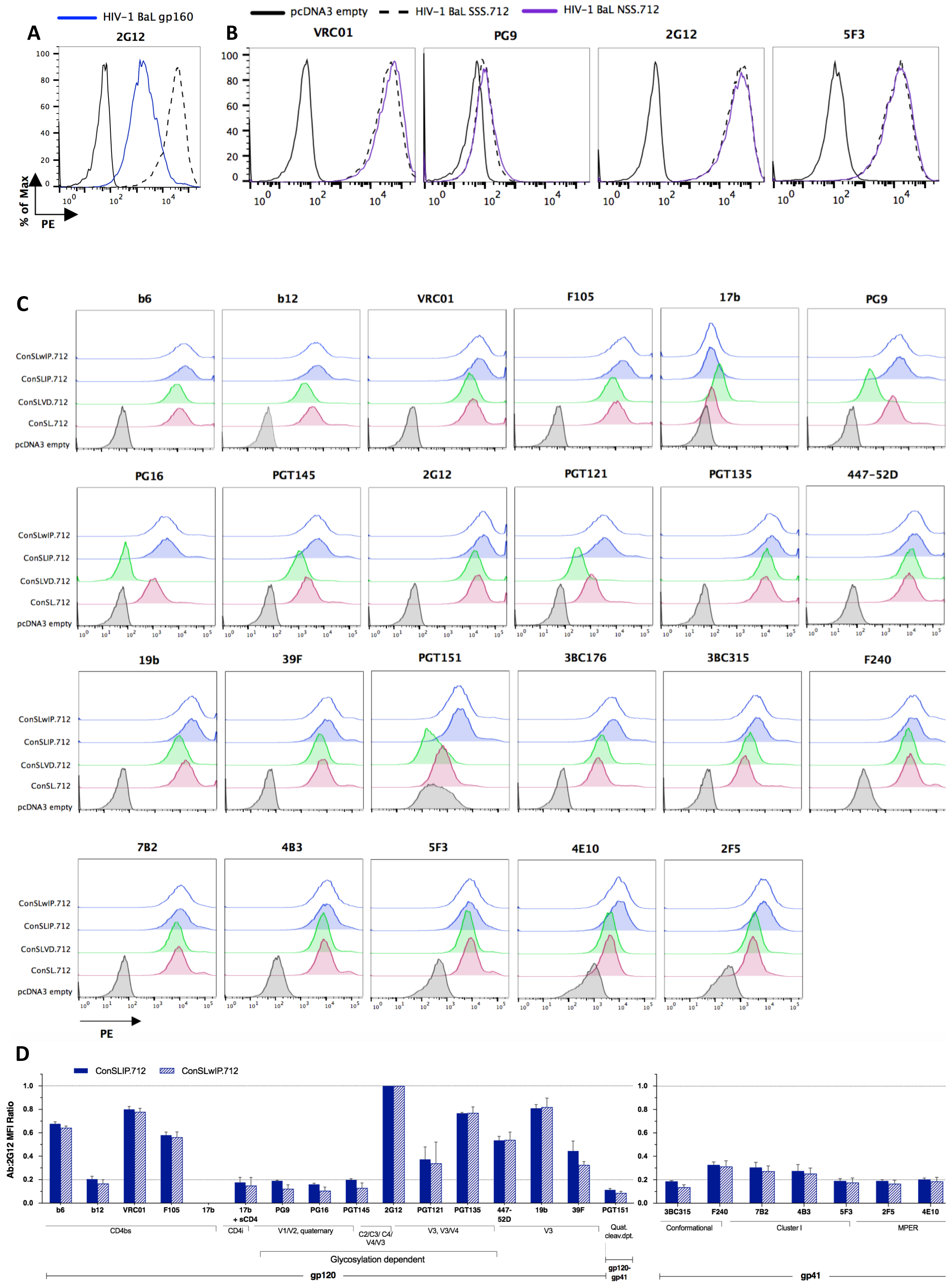

Figure S1. Effects of truncation at position 712 and signal sequence on surface expression of BaL and impact of IP, VD and linker orientation on the cleavage independent ConSL.712 design. Related to Figures 2 and 3. (**A**) FC analysis of HIV-1 BaL Env comparing the expression levels of gp160 and truncated version BaL SSS.712 transiently expressed in 293T.17 cells. Representative of n ≥ 2 independent experiments.

(**B**) Comparison of native signal sequence (NSS) versus secretion signal sequence (SSS) and their impact on Env surface antigenicity in 293T.17 cells. 293T.17 cells transfected with a pcDNA3 empty vector were included as a negative control to determine each mAb background. Representative of $n \geq 2$ independent experiments. (**C**) 293T.17 cells were transiently transfected with the indicated constructs and traces were plotted in FlowJo. Representative of $n \geq 2$ independent experiments. (**D**) Ab binding profile of ConSLIP.712 and ConSLwIP.712 were analysed as in Figure 2. Error bars represent mean ± SEM with $n \geq 2$ independent experiments.

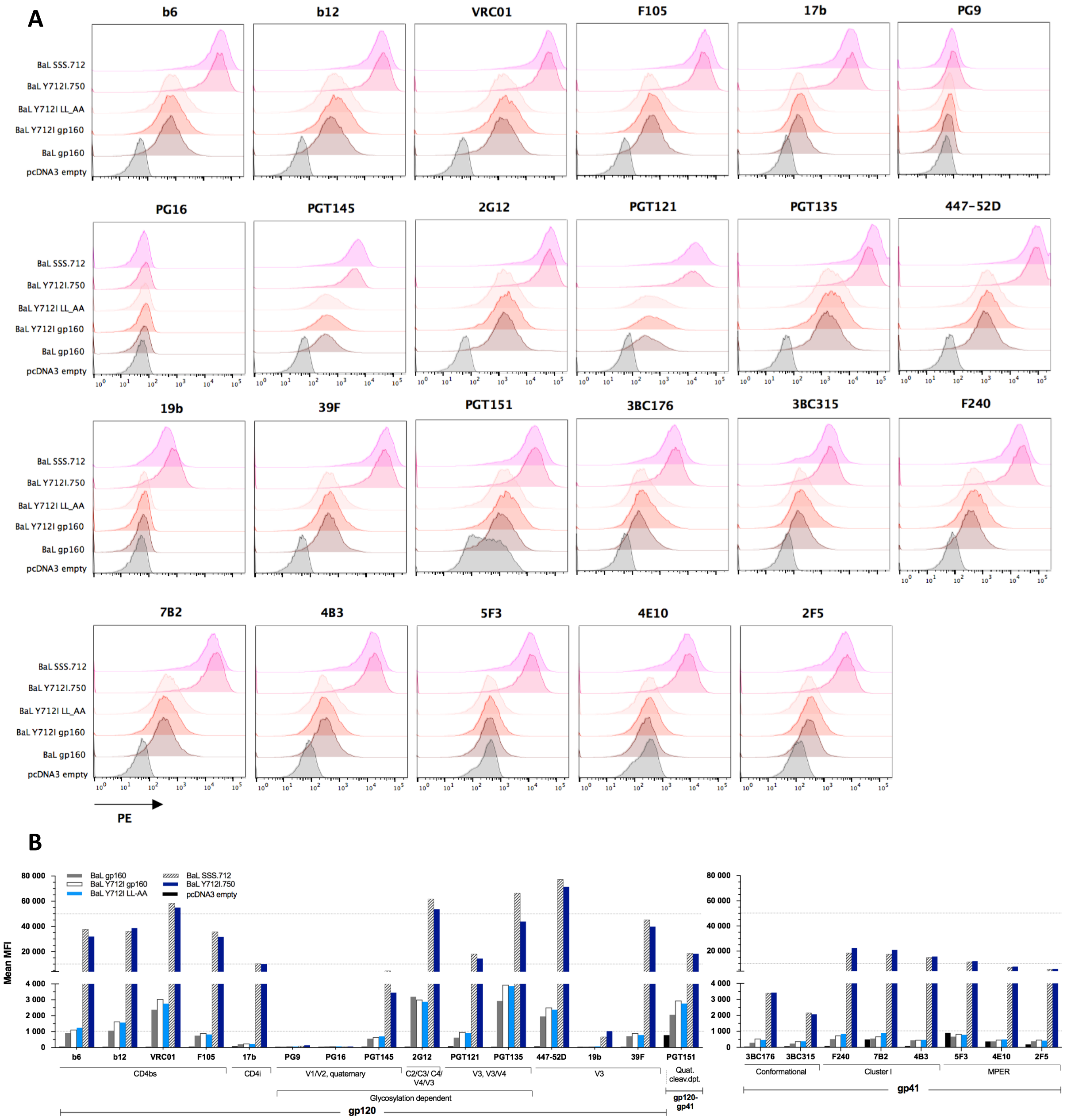

Figure S2. Effect of the endocytosis/recycling motif mutations and CT truncations on BaL Env surface expression. Related to Figure 3. (**A**) Traces overlay of extended BaL constructs FC data from Figure 3. (**B**) Extended mAbs panel from Figure 3.B with mean MFI values plotted. Representative of n ≥ 2 independent experiments.

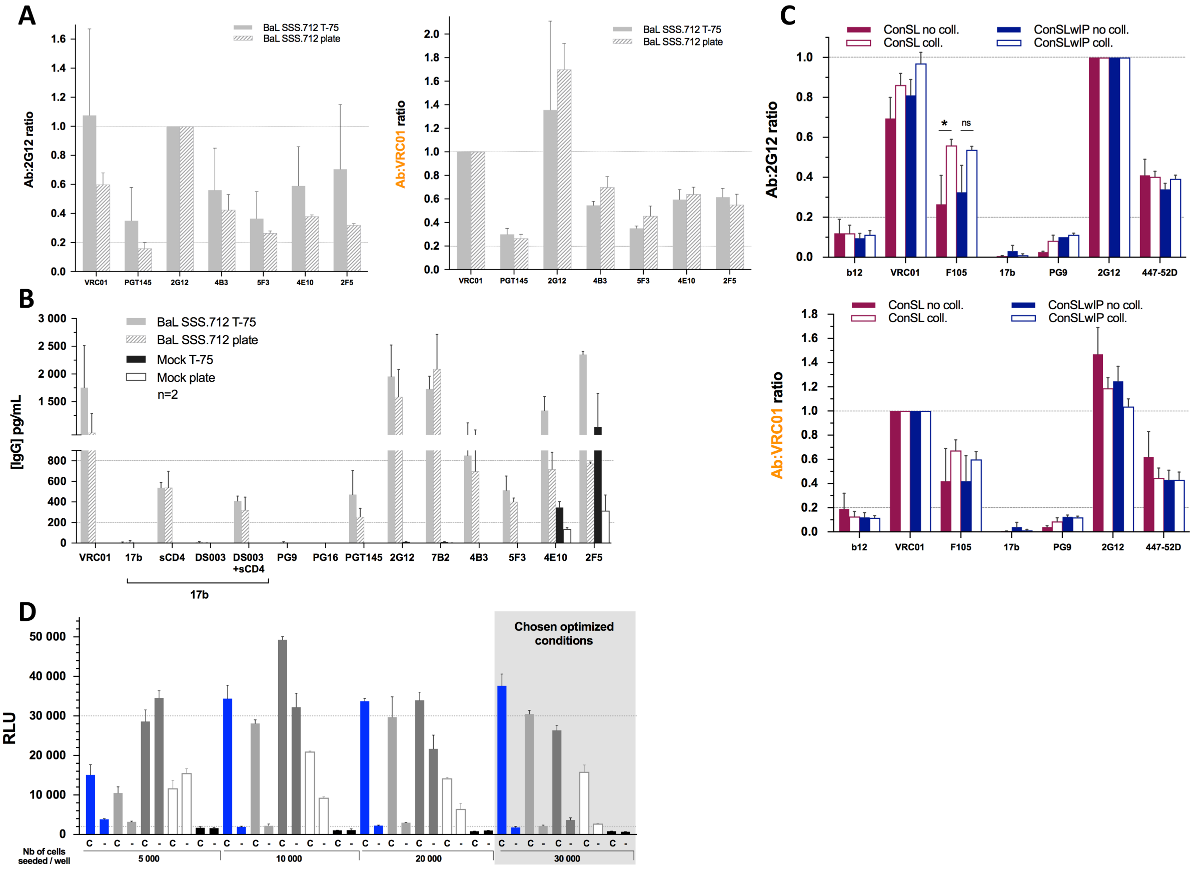

Figure S3. Cell ELISA assay optimization and validation for 293T.17 and human skeletal primary muscle cells. Related to Figure 3. (**A**) Comparison of CE transfection and seeded conditions using BaL SSS.712 expressing 293T.17 cells either transfected in T-75 then seeded onto the ELISA plate or cells directly seeded and transfected onto the plate. Both mAb:2G12 and mAb:VRC01 are shown (n ≥ 2). (**B**) Same as (**A**) using an extended panel and a standard IgG. DS003 is a microbicide from the International Partnership Microbicide and binds the CD4bs. (**C**) Comparison of collagen coated (coll.) plates versus non coated (no coll.) plates. Both mAb:2G12 and mAb:VRC01 are shown (n ≥ 2). (**D**) Muscle cells CE optimization using 4 mAbs at 10 µg/mL (2G12 blue, VRC01 light grey, PGT135 dark grey, F240 white) and different cell concentration seeded per well. ConSLIP.712 (‘C’ on the x axis) was used for optimization along with pcDNA3 empty (‘-‘ on the x axis) vector transfected wells to determine mAbs background. Best conditions are highlighted in grey. Error bars represent mean ± SEM with n ≥ 2 independent experiments for (**A**), (**B**) and (**C**) and mean ± SD of duplicate wells for (**D**). Unpaired t test *p* values: *<0.05.

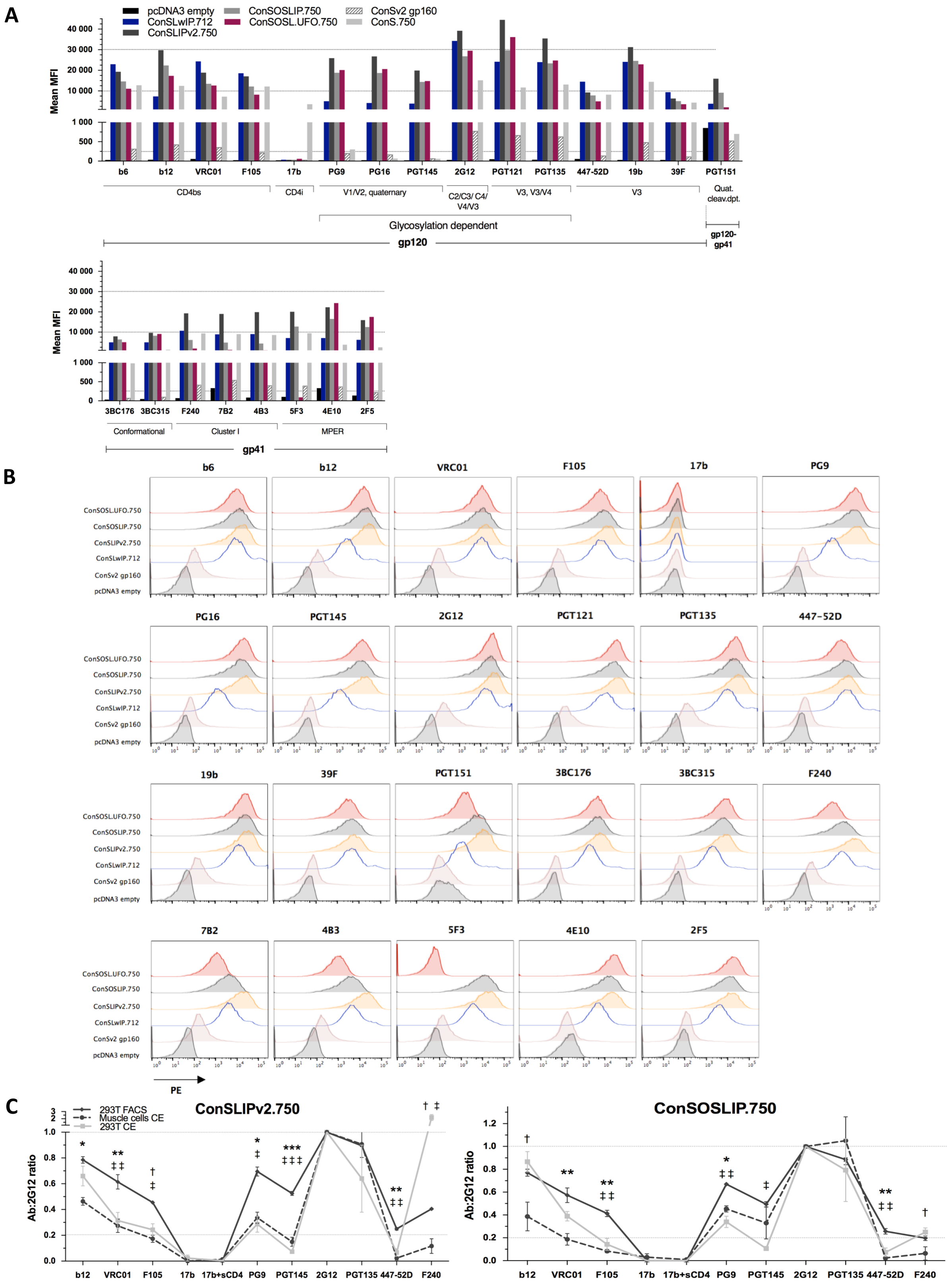

Figure S4. Enhancement of bNAbs binding and subsequent reduction of nNAbs binding by gp41 HR1 stabilization - Flow cytometry and cell ELISA. Related to Figure 4. (**A**) Mean MFI values corresponding to the bar graph of Figure 4.A of Env constructs expressed in 293T.17 and analysed by FC for 23 mAb. Representative of n ≥ 2 independent experiments (for ConSOSL.UFO.750 n ≥ 3). (**B**) Traces overlay of the corresponding experiment expressed in 293T.17 cells. Representative of n ≥ 2 independent experiments (ConSOSL.UFO.750 n ≥ 3). (**C**) Comparison of ConSLIPv2.750 and ConSOSLIP.750 epitope profiles in

muscle cell CE, 293T.17 CE and FC ($n \geq 2$). One-way ANOVA with Sidak's multiple comparisons, *p* values: *<0.05, **<0.01, ***<0.001 where each sign in (**C**) compares: *Muscle Cells CE vs. 293T.17 FC; † Muscle Cells CE vs. 293T.17 CE; ‡ 293T.17 FC vs. 293T CE.

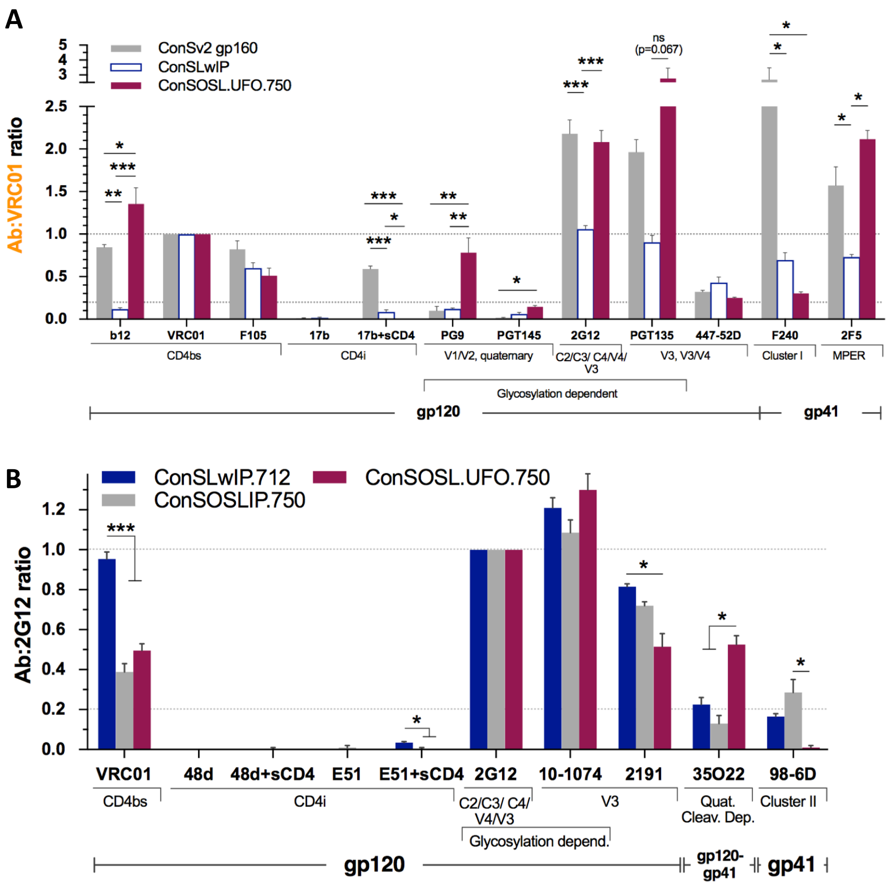

Figure S5. Enhancement of bNAbs binding and subsequent reduction of nNAbs binding by gp41 HR1 stabilization – Cell ELISA. Related to Figure 4. (**A**) 293T.17 CE assessing ConSv2 gp160, ConSLwIP.712 and ConSOSL.UFO.750 using mAb:VRC01 ratio to plot the mean ± SEM values ($n \geq 3$, except ConSv2 gp160 PGT135 $n = 2$). (**B**) Comparison of ConSLwIP.712, ConSOSLIP.750 and ConSOSL.UFO.750 in 293T.17 CE for mAb: VRC01, 48d (+/- sCD4), E51 (+/- sCD4), 2G12, 10-1074, 2191, 35O22 and 98-6D ($n \geq 2$). One-way ANOVA with Sidak's multiple comparisons, p values: *<0.05, **<0.01, ***<0.001.

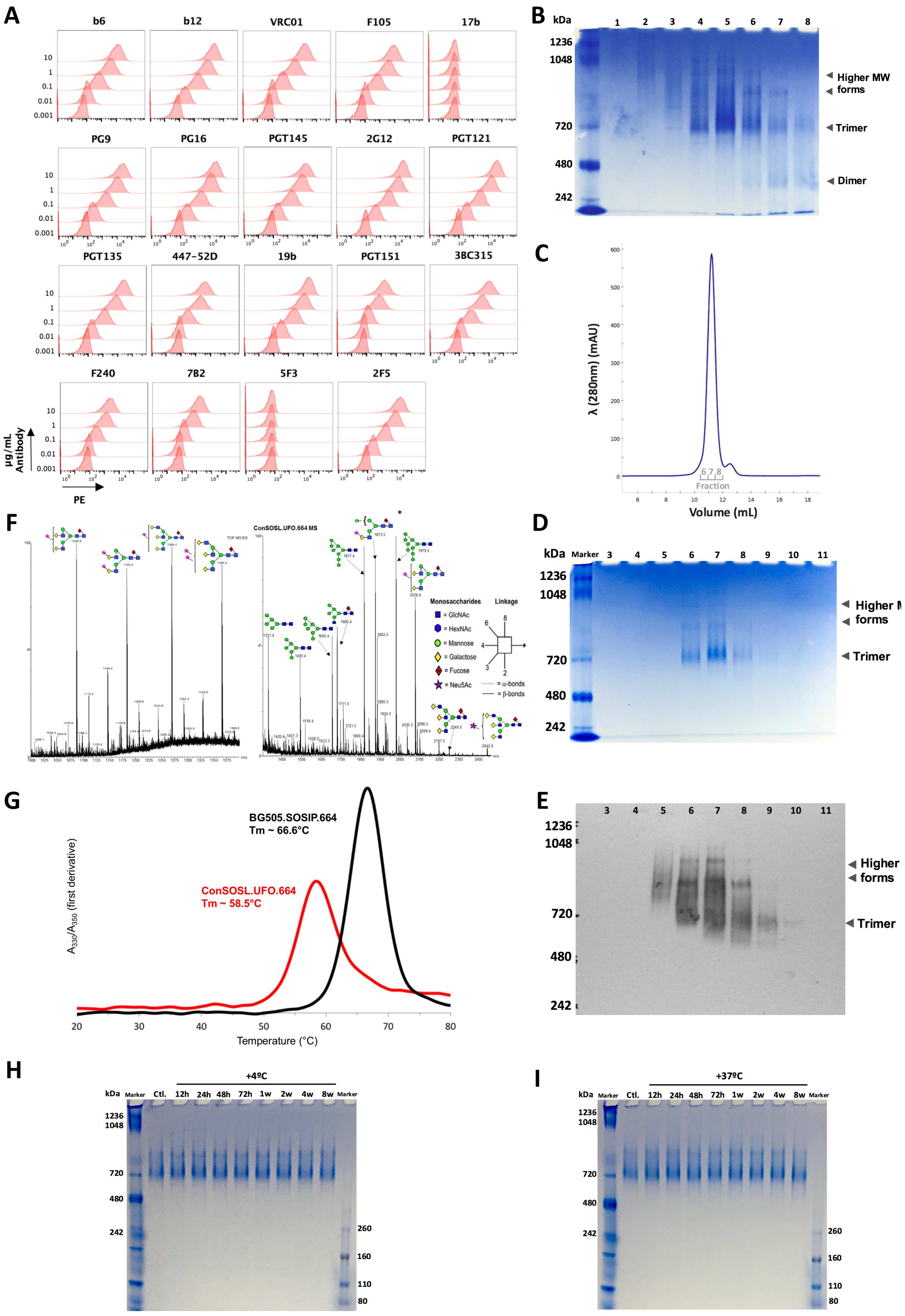

Figure S6. Antibodies titration against ConSOSL.UFO.750 and structural and glycosylation analysis of ConSOSL.UFO.664 Env trimers. Related to Figures 5 and 6. (**A**) Traces corresponding to the mean MFI value plotted in Figure 5. Representative of n = 2 independent experiments. (**B**) Colloidal blue stain of a native

gel running fractions of the SEC purification from Figure 6.A. (**C**) Fractions 4-6 of Figure 6.A were run a second time through a SEC column and the obtained SEC profile is shown. (**D**) Colloidal blue stain of a native gel running fractions of the SEC purification from (**C**). (**E**) Native PAGE western blot of (**D**) using 2G12 (1 µg/mL) for detection. **(B)-(E)** Representative of $n \geq 3$ independent experiments. (**F**) Mass spectrometry spectra of singly (left panel) and doubly charged ions extracted using driftscope. Abundant glycan structures and larger complex glycans are labelled. The m/z=1873 is a hybrid lacking a mannose on 6 arm, but both isomers are present. (**F**) Thermal stability assessment of ConSOSL.UFO.664 (red) using nanoDSF, Tm is indicated. BG505 SOSIP.664 (black) trimer is also plotted for comparison. (**H**) Thermostability of ConSOSL.UFO.664 at +4ºC and (**I**) +37ºC over 8 weeks, representative of n = 1 experiment.

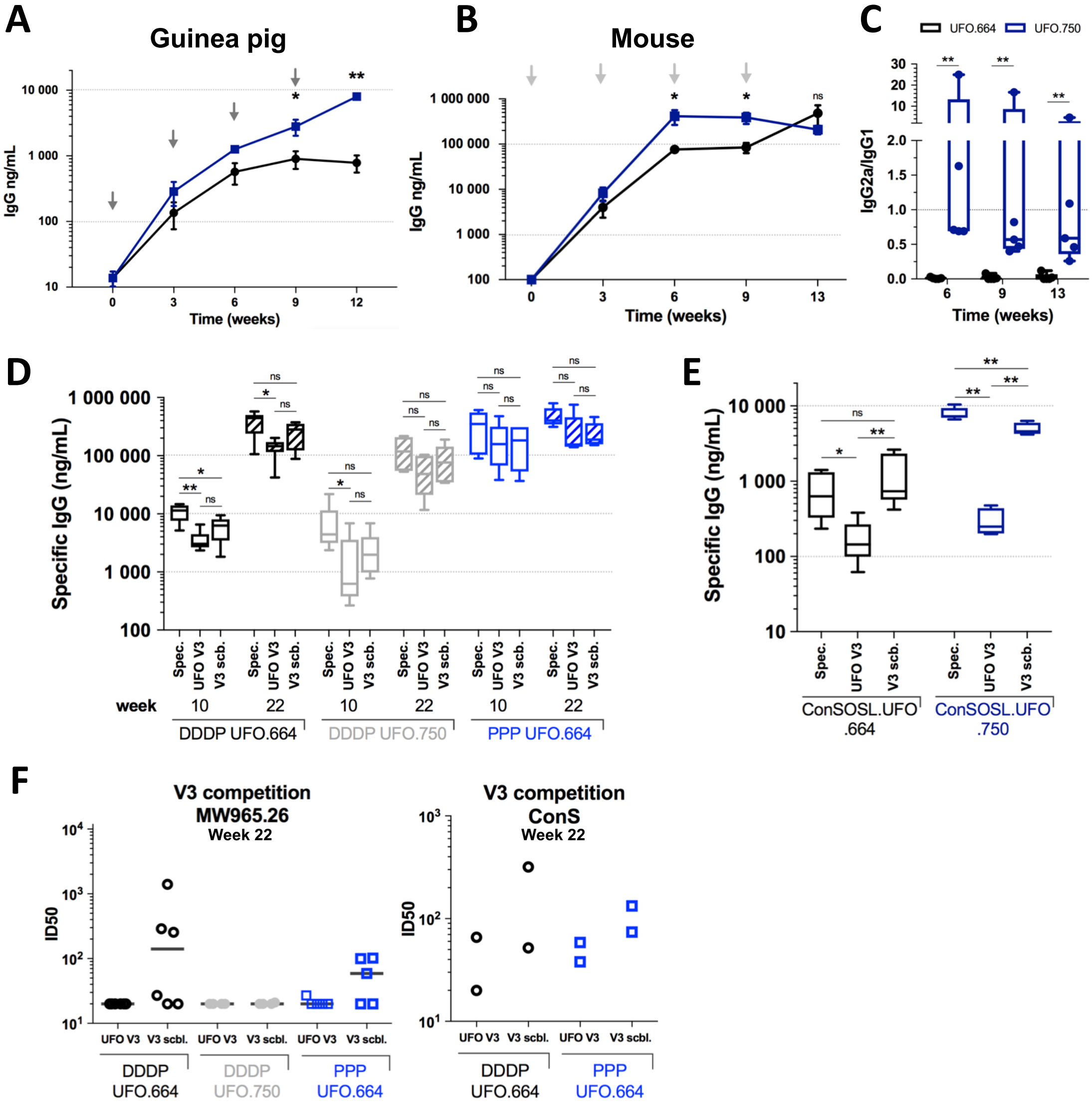

Figure S7. Immunogenicity of ConSOSL.UFO design in Guinea pigs and mice and V3 specific response in rabbits. Related to Figure 7. (**A**) Antigen specific serum IgG binding for DNA immunized Guinea pigs and (**B**) mice with ConSOSL.UFO.750 plasmid (blue) and ConSOSL.UFO.664 plasmid (black). Binding assessed by 9E10 capture ELISA for Guinea pig's sera and by directly coated ConSOSL.UFO.664 trimers for mouse sera. Arrows indicate immunization with DNA IM+EP. For (**A**) time points 0, 3 and 6, n = 6 animals per group, then for time points 9 and 12, n = 5. Mice, n = 5 per group. Error bars represent mean ± SEM values. (**C**) Mouse IgG1 and IgG2a antigen specific responses were assessed by ELISA and the IgG1/IgG2a ratio is shown here. (**D**) Rabbit serum samples from week 10 and 22 and (**E**) Guinea pig's serum samples from week 12 were incubated 30min prior loading onto the ELISA plates with 10ug/mL ConSOSL.UFO V3 cys-cyclised (UFO V3) peptide or MN.3 V3 scrambled linear (V3 scbl.) peptide as a control and the specific binding to ConSOSL.UFO.664 Myc-HIS captured antigen measured. The total specific response is plotted and referred as 'Spec.'. (**F**) Neutralization of HIV-1 MW965.26 and ConS pseudoviruses in TZM-bl assay by rabbit neutralizer sera from week 22 in competition with UFO V3 and V3 scbl. peptides. Data plotted as ID50 values (serum dilution that inhibits by 50% infectivity). Unpaired t-test, p values: *<0.05, **<0.01.

```
BG505_gp160_DQ208458_1      MRVMGIQRNCQHLFRWGTMILGMIIICSAAENLWVTVYYGVPVWKDAETTLFCASDAKAY     60
ConS_gp160                  MRVRGIQRNCQHLWRWGTLILGMLMICSAAENLWVTVYYGVPVWKEANTTLFCASDAKAY     60
ConSOSIP_712                ---------MDRAKLLLLLLLLLLPQAQAVENLWVTVYYGVPVWKEAETTLFCASDAKAY     51
ConSOSVD_712                ---------MDRAKLLLLLLLLLLPQAQAVENLWVTVYYGVPVWKEAETTLFCASDAKAY     51
ConSL_712                   ---------MDRAKLLLLLLLLLLPQAQAVENLWVTVYYGVPVWKEAETTLFCASDAKAY     51
ConSLVD_712                 ---------MDRAKLLLLLLLLLLPQAQAVENLWVTVYYGVPVWKEAETTLFCASDAKAY     51
ConSLIP_712                 ---------MDRAKLLLLLLLLLLPQAQAVENLWVTVYYGVPVWKEAETTLFCASDAKAY     51
ConSLwIP_712                ---------MDRAKLLLLLLLLLLPQAQAVENLWVTVYYGVPVWKEAETTLFCASDAKAY     51
ConSLIPv2_750               ---------MDRAKLLLLLLLLLLPQAQAVENLWVTVYYGVPVWKDAETTLFCASDAKAY     51
ConSOSLIP_750               ---------MDRAKLLLLLLLLLLPQAQAVENLWVTVYYGVPVWKDAETTLFCASDAKAY     51
ConSOSL_UFO_750             ---------MDRAKLLLLLLLLLLPQAQAVENLWVTVYYGVPVWKDAETTLFCASDAKAY     51
ConSOSL_UFO_664             ---------MDRAKLLLLLLLLLLPQAQAVENLWVTVYYGVPVWKDAETTLFCASDAKAY     51
ConSv2_gp160                ---------MDRAKLLLLLLLLLLPQAQAVENLWVTVYYGVPVWKDAETTLFCASDAKAY     51
                            |----   -Signal-Sequence  --||------------------------------
                            |           |               |         |         |         |
                            1           10              30        40        50        60

BG505_gp160_DQ208458_1      ETEKHNVWATHACVPTDPNPQEIHLENVTEEFNMWKNNMVEQMHTDIISLWDQSLKPCVK     120
ConS_gp160                  DTEVHNVWATHACVPTDPNPQEIVLENVTENFNMWKNNMVEQMHEDIISLWDQSLKPCVK     120
ConSOSIP_712                DTEKHNVWATHACVPTDPNPQEIHLENVTEEFNMWKNNMVEQMHTDIISLWDQSLKPCVK     111
ConSOSVD_712                DTEKHNVWATHACVPTDPNPQEIHLENVTEEFNMWKNNMVEQMHTDIISLWDQSLKPCVK     111
ConSL_712                   DTEKHNVWATHACVPTDPNPQEIHLENVTEEFNMWKNNMVEQMHTDIISLWDQSLKPCVK     111
ConSLVD_712                 DTEKHNVWATHACVPTDPNPQEIHLENVTEEFNMWKNNMVEQMHTDIISLWDQSLKPCVK     111
ConSLIP_712                 DTEKHNVWATHACVPTDPNPQEIHLENVTEEFNMWKNNMVEQMHTDIISLWDQSLKPCVK     111
ConSLwIP_712                DTEKHNVWATHACVPTDPNPQEIHLENVTEEFNMWKNNMVEQMHTDIISLWDQSLKPCVK     111
ConSLIPv2_750               DTEVRNVWATHACVPTDPNPQEIVLENVTENFNMWKNNMVEQMHTDIISLWDQSLKPCVK     111
ConSOSLIP_750               DTEVRNVWATHACVPTDPNPQEIVLENVTENFNMWKNNMVEQMHTDIISLWDQSLKPCVK     111
ConSOSL_UFO_750             DTEVRNVWATHACVPTDPNPQEIVLENVTENFNMWKNNMVEQMHTDIISLWDQSLKPCVK     111
ConSOSL_UFO_664             DTEVRNVWATHACVPTDPNPQEIVLENVTENFNMWKNNMVEQMHTDIISLWDQSLKPCVK     111
ConSv2_gp160                DTEVRNVWATHACVPTDPNPQEIVLENVTENFNMWKNNMVEQMHTDIISLWDQSLKPCVK     111
                            --------------------C1-Domain---------------------------||--
                                    |         |         |         |         |         |
                                    70        80        90        100       110       120

BG505_gp160_DQ208458_1      LTPLCVTLQCTNVTNNI---TDDMRGELKNCSFNMTTELRDKKQKVYSLFYRLDVVQINE     177
ConS_gp160                  LTPLCVTLNCTNVNVTNTTNNTEEKGEIKNCSFNITTEIRDKKQKVYALFYRLDVVPIDD     180
ConSOSIP_712                LTPLCVTLNCTNVNVTNTTNNTEEKGEIKNCSFNMTTELRDKKQKVYSLFYRLDVVPIDD     171
ConSOSVD_712                LTPLCVTLNCTNVNVTNTTNNTEEKGEIKNCSFNMTTELRDKKQKVYSLFYRLDVVPIDD     171
ConSL_712                   LTPLCVTLNCTNVNVTNTTNNTEEKGEIKNCSFNMTTELRDKKQKVYSLFYRLDVVPIDD     171
ConSLVD_712                 LTPLCVTLNCTNVNVTNTTNNTEEKGEIKNCSFNMTTELRDKKQKVYSLFYRLDVVPIDD     171
ConSLIP_712                 LTPLCVTLNCTNVNVTNTTNNTEEKGEIKNCSFNMTTELRDKKQKVYSLFYRLDVVPIDD     171
ConSLwIP_712                LTPLCVTLNCTNVNVTNTTNNTEEKGEIKNCSFNMTTELRDKKQKVYSLFYRLDVVPIDD     171
ConSLIPv2_750               LTPLCVTLNCTNVNVTNTTNNTEEKGEIKNCSFNITTELRDKKKKVYALFYRLDVVPIDD     171
ConSOSLIP_750               LTPLCVTLNCTNVNVTNTTNNTEEKGEIKNCSFNITTELRDKKKKVYALFYRLDVVPIDD     171
ConSOSL_UFO_750             LTPLCVTLNCTNVNVTNTTNNTEEKGEIKNCSFNITTELRDKKKKVYALFYRLDVVPIDD     171
ConSOSL_UFO_664             LTPLCVTLNCTNVNVTNTTNNTEEKGEIKNCSFNITTELRDKKKKVYALFYRLDVVPIDD     171
ConSv2_gp160                LTPLCVTLNCTNVNVTNTTNNTEEKGEIKNCSFNITTELRDKKKKVYALFYRLDVVPIDD     171
                            ------V1-Domain------ --------||------------V2-Domain-------
                                    |         |    |         |         |         |
                                    130       140  150       160       170       180

BG505_gp160_DQ208458_1      NQGNRSNNSNKEYRLINCNTSAITQACPKVSFEPIPIHYCAPAGFAILKCKDKKFNGTGP     237
ConS_gp160                  NNN-----NSSNYRLINCNTSAITQACPKVSFEPIPIHYCAPAGFAILKCNDKKFNGTGP     235
ConSOSIP_712                NNN-----NSSNYRLINCNTSAITQACPKVSFEPIPIHYCAPAGFAILKCNDKKFNGTGP     226
ConSOSVD_712                NNN-----NSSNYRLINCNTSACTQACPKVSFEPIPIHYCAPAGFAILKCNDKKFNGTGP     226
ConSL_712                   NNN-----NSSNYRLINCNTSAITQACPKVSFEPIPIHYCAPAGFAILKCNDKKFNGTGP     226
ConSLVD_712                 NNN-----NSSNYRLINCNTSACTQACPKVSFEPIPIHYCAPAGFAILKCNDKKFNGTGP     226
ConSLIP_712                 NNN-----NSSNYRLINCNTSAITQACPKVSFEPIPIHYCAPAGFAILKCNDKKFNGTGP     226
ConSLwIP_712                NNN-----NSSNYRLINCNTSAITQACPKVSFEPIPIHYCAPAGFAILKCNDKKFNGTGP     226
ConSLIPv2_750               NNN-----NSSNYRLINCNTSAITQACPKVSFEPIPIHYCAPAGFAILKCNDKKFNGTGP     226
ConSOSLIP_750               NNN-----NSSNYRLINCNTSAITQACPKVSFEPIPIHYCAPAGFAILKCNDKKFNGTGP     226
ConSOSL_UFO_750             NNN-----NSSNYRLINCNTSAITQACPKVSFEPIPIHYCAPAGFAILKCNDKKFNGTGP     226
ConSOSL_UFO_664             NNN-----NSSNYRLINCNTSAITQACPKVSFEPIPIHYCAPAGFAILKCNDKKFNGTGP     226
ConSv2_gp160                NNN-----NSSNYRLINCNTSAITQACPKVSFEPIPIHYCAPAGFAILKCNDKKFNGTGP     226
                                    ------------------||--------------C2-Domain---------
                                       |         |         |         |         |
                                       190       200       210       220       230
```

```
BG505_gp160_DQ208458_1   CPSVSTVQCTHGIKPVVSTQLLLNGSLAEEEVMIRSENITNNAKNILVQFNTPVQINCTR   297
ConS_gp160               CKNVSTVQCTHGIKPVVSTQLLLNGSLAEEEIIIRSENITNNAKTIIVQLNESVEINCTR   295
ConSOSIP_712             CKNVSTVQCTHGIKPVVSTQLLLNGSLAEEEVMIRSENITNNAKNILVQFNTPVQINCTR   286
ConSOSVD_712             CKNVSTVQCTHGIKPVVSTQLLLNGSLAEEEVMIRSENITNNAKNILVQFNTPVQINCTR   286
ConSL_712                CKNVSTVQCTHGIKPVVSTQLLLNGSLAEEEVMIRSENITNNAKNILVQFNTPVQINCTR   286
ConSLVD_712              CKNVSTVQCTHGIKPVVSTQLLLNGSLAEEEVMIRSENITNNAKNILVQFNTPVQINCTR   286
ConSLIP_712              CKNVSTVQCTHGIKPVVSTQLLLNGSLAEEEVMIRSENITNNAKNILVQFNTPVQINCTR   286
ConSLwIP_712             CKNVSTVQCTHGIKPVVSTQLLLNGSLAEEEVMIRSENITNNAKNILVQFNTPVQINCTR   286
ConSLIPv2_750            CKNVSTVQCTHGIKPVVSTQLLLNGSLAEEEIIIRSENITNNAKTIIVQLNESVEINCTR   286
ConSOSLIP_750            CKNVSTVQCTHGIKPVVSTQLLLNGSLAEEEIIIRSENITNNAKTIIVQLNESVEINCTR   286
ConSOSL_UFO_750          CKNVSTVQCTHGIKPVVSTQLLLNGSLAEEEIIIRSENITNNAKTIIVQLNESVEINCTR   286
ConSOSL_UFO_664          CKNVSTVQCTHGIKPVVSTQLLLNGSLAEEEIIIRSENITNNAKTIIVQLNESVEINCTR   286
ConSv2_gp160             CKNVSTVQCTHGIKPVVSTQLLLNGSLAEEEIIIRSENITNNAKTIIVQLNESVEINCTR   286
                         ----------------------------C2-Domain--------------------||-
                          |         |         |         |         |         |
                         240       250       260       270       280       290

BG505_gp160_DQ208458_1   PNNNTRKSIRIGPGQAFYATGDIIGDIRQAHCTVSKATWNETLGKVVKQLRKHFGNNTII   357
ConS_gp160               PNNNTRKSIRIGPGQAFYATGDIIGDIRQAHCNISGTKWNKTLQQVAKKLREHFNNKTI-   354
ConSOSIP_712             PNNNTRKSIRIGPGQAFYATGDIIGDIRQAHCNVSKATWNETLGKVVKQLRKHFGNNTII   346
ConSOSVD_712             PNNNTRKSIRIGPGQAFYATGDIIGDIRQAHCNVSKATWNETLGKVVKQLRKHFGNNTII   346
ConSL_712                PNNNTRKSIRIGPGQAFYATGDIIGDIRQAHCNVSKATWNETLGKVVKQLRKHFGNNTII   346
ConSLVD_712              PNNNTRKSIRIGPGQAFYATGDIIGDIRQAHCNVSKATWNETLGKVVKQLRKHFGNNTII   346
ConSLIP_712              PNNNTRKSIRIGPGQAFYATGDIIGDIRQAHCNVSKATWNETLGKVVKQLRKHFGNNTII   346
ConSLwIP_712             PNNNTRKSIRIGPGQAFYATGDIIGDIRQAHCNVSKATWNETLGKVVKQLRKHFGNNTII   346
ConSLIPv2_750            PNNNTRKSIRIGPGQWFYATGDIIGDIRQAHCNISGTKWNKTLQQVVKKLREHFNNKTI-   345
ConSOSLIP_750            PNNNTRKSIRIGPGQWFYATGDIIGDIRQAHCNISGTKWNKTLQQVVKKLREHFNNKTI-   345
ConSOSL_UFO_750          PNNNTRKSIRIGPGQWFYATGDIIGDIRQAHCNISGTKWNKTLQQVVKKLREHFNNKTI-   345
ConSOSL_UFO_664          PNNNTRKSIRIGPGQWFYATGDIIGDIRQAHCNISGTKWNKTLQQVVKKLREHFNNKTI-   345
ConSv2_gp160             PNNNTRKSIRIGPGQWFYATGDIIGDIRQAHCNISGTKWNKTLQQVVKKLREHFNNKTI-   345
                         -----------------V3-Loop- ----||--------------C3-Domain-----
                          |       ||       |          |         |         |
                         300   308/311    320        330       340       350

BG505_gp160_DQ208458_1   RFANSSGGDLEVTTHSFNCGGEFFYCNTSGLFNSTWISNTSVQGSNSTGSNDSITLPCRI   417
ConS_gp160               IFKPSSGGDLEITTHSFNCRGEFFYCNTSGLFNSTWIGNGTKN---NNNTNDTITLPCRI   411
ConSOSIP_712             RFANSSGGDLEVTTHSFNCGGEFFYCNTSGLFNSTWIGNGTKN---NNNTNDTITLPCRI   403
ConSOSVD_712             RFANSSGGDLEVTTHSFNCGGEFFYCNTSGLFNSTWIGNGTKN---NNNTNDTITLPCRI   403
ConSL_712                RFANSSGGDLEVTTHSFNCGGEFFYCNTSGLFNSTWIGNGTKN---NNNTNDTITLPCRI   403
ConSLVD_712              RFANSSGGDLEVTTHSFNCGGEFFYCNTSGLFNSTWIGNGTKN---NNNTNDTITLPCRI   403
ConSLIP_712              RFANSSGGDLEVTTHSFNCGGEFFYCNTSGLFNSTWIGNGTKN---NNNTNDTITLPCRI   403
ConSLwIP_712             RFANSSGGDLEVTTHSFNCGGEFFYCNTSGLFNSTWIGNGTKN---NNNTNDTITLPCRI   403
ConSLIPv2_750            IFNPSSGGDLEITTHSFNCGGEFFYCNTSGLFNSTWIGNGTKN---NNNTNDTITLPCRI   402
ConSOSLIP_750            IFNPSSGGDLEITTHSFNCGGEFFYCNTSGLFNSTWIGNGTKN---NNNTNDTITLPCRI   402
ConSOSL_UFO_750          IFNPSSGGDLEITTHSFNCGGEFFYCNTSGLFNSTWIGNGTKN---NNNTNDTITLPCRI   402
ConSOSL_UFO_664          IFNPSSGGDLEITTHSFNCGGEFFYCNTSGLFNSTWIGNGTKN---NNNTNDTITLPCRI   402
ConSv2_gp160             IFNPSSGGDLEITTHSFNCGGEFFYCNTSGLFNSTWIGNGTKN---NNNTNDTITLPCRI   402
                         -------------------------||----V4-Loop------------------||--
                         |         |         |         |      ||         |         |
                        360       370       380       390   399/401     410       420

BG505_gp160_DQ208458_1   KQIINMWQRIGQAMYAPPIQGVIRCVSNITGLILTRDGGSTNS-TTETFRPGGGDMRDNW   476
ConS_gp160               KQIINMWQGVGQAMYAPPIEGKITCKSNITGLLLTRDGGNNNTNETEIFRPGGGDMRDNW   471
ConSOSIP_712             KQIINMWQRIGQAMYAPPIQPVIRCVSNITGLILTRDGGSTNS-TTETFRPGGGDMRDNW   462
ConSOSVD_712             KQIINMWQRIGQCMYAPPIQCVIRCVSNITGLILTRDGGSTNS-TTETFRPGGGDMRDNW   462
ConSL_712                KQIINMWQRIGQPMYAPPIQGVIRCVSNITGLILTRDGGSTNS-TTETFRPGGGDMRDNW   462
ConSLVD_712              KQIINMWQRIGQCMYAPPIQGVIRCVSNITGLILTRDGGSTNS-TTETFRPGGGDMRDNW   462
ConSLIP_712              KQIINMWQRIGQPMYAPPIQGVIRCVSNITGLILTRDGGSTNS-TTETFRPGGGDMRDNW   462
ConSLwIP_712             KQIINMWQRIGQPMYAPPIQGVIRCVSNITGLILTRDGGSTNS-TTETFRPGGGDMRDNW   462
ConSLIPv2_750            KQIINMWQRVGQPMYAPPIQGKIRCVSNITGLLLTRDGGNNNTNETETFRPGGGDMRDNW   462
ConSOSLIP_750            KQIINMWQRVGQPMYAPPIQGKIRCVSNITGLLLTRDGGNNNTNETETFRPGGGDMRDNW   462
ConSOSL_UFO_750          KQIINMWQRVGQPMYAPPIQGKIRCVSNITGLLLTRDGGNNNTNETETFRPGGGDMRDNW   462
ConSOSL_UFO_664          KQIINMWQRVGQPMYAPPIQGKIRCVSNITGLLLTRDGGNNNTNETETFRPGGGDMRDNW   462
ConSv2_gp160             KQIINMWQRVGQAMYAPPIQGKIRCVSNITGLLLTRDGGNNNTNETETFRPGGGDMRDNW   462
                         ---------------C4-Domain-------------||V5Lp||-b24--||----C5-
                                  |         |         |         |          |        |
                                 430       440       450       460        470      479
```

```
BG505_gp160_DQ208458_1   RSELYKYKVVKIEPLGVAPTRAKRRVVGREKR----------AVGIGAVFLGFLGAAGST  526
ConS_gp160               RSELYKYKVVKIEPLGVAPTKAKRRVVEREKR----------AVGIGAVFLGFLGAAGST  521
ConSOSIP_712             RSELYKYKVVKIEPLGVAPTRCKRRVVGRRRR--------RRAVGIGAVFLGFLGAAGST  514
ConSOSVD_712             RSELYKYKVVKIEPLGVAPTRCKRRVVGRRRR--------RRAVGIGAVFLGFLGAAGST  514
ConSL_712                RSELYKYKVVKIEPLGVAPTKAKRRVVESEKRSGGGGSGGGGAVGIGAVFLGFLGAAGST  522
ConSLVD_712              RSELYKYKVVKIEPLGVAPTKAKRRVVESEKRSGGGGSGGGGAVGIGAVFLGFLGAAGST  522
ConSLIP_712              RSELYKYKVVKIEPLGVAPTKAKRRVVESEKRSGGGGSGGGGAVGIGAVFLGFLGAAGST  522
ConSLwIP_712             RSELYKYKVVKIEPLGVAPTKAKRRVVESEKRGGGGSGGGGSAVGIGAVFLGFLGAAGST  522
ConSLIPv2_750            RSELYKYKVVKIEPLGVAPTRAKRRVVESEKRGGGGSGGGGSAVGIGAVFLGFLGAAGST  522
ConSOSLIP_750            RSELYKYKVVKIEPLGVAPTRCKRRVVE----GGGGSGGGGSAVGIGAVFLGFLGAAGST  518
ConSOSL_UFO_750          RSELYKYKVVKIEPLGVAPTRCKRRVVE----GGGGSGGGGSAVGIGAVFLGFLGAAGST  518
ConSOSL_UFO_664          RSELYKYKVVKIEPLGVAPTRCKRRVVE----GGGGSGGGGSAVGIGAVFLGFLGAAGST  518
ConSv2_gp160             RSELYKYKVVKIEPLGVAPTRAKRRVVEREKR----------AVGIGAVFLGFLGAAGST  512
                         ---------C5-Domain-----------|           |----------HR-1--
                         |         |         |         |                   |
                        480       490       500       510                 520

BG505_gp160_DQ208458_1   MGAASMTLTVQARNLLSGIVQQQSNLLRAIEAQQHLLKLTVWGIKQLQARVLAVERYLRD  586
ConS_gp160               MGAASITLTVQARQLLSGIVQQQSNLLRAIEAQQHLLQLTVWGIKQLQARVLAVERYLKD  581
ConSOSIP_712             MGAASMTLTVQARNLLSGIVQQQSNLLRAPEAQQHLLKLTVWGIKQLQARVLAVERYLRD  574
ConSOSVD_712             MGAASMTLTVQARNLLSGIVQQQSNLLRAIEAQQHLLKLTDWGIKQLQARVLAVERYLRD  574
ConSL_712                MGAASMTLTVQARNLLSGIVQQQSNLLRAIEAQQHLLKLTVWGIKQLQARVLAVERYLRD  582
ConSLVD_712              MGAASMTLTVQARNLLSGIVQQQSNLLRAIEAQQHLLKLTDWGIKQLQARVLAVERYLRD  582
ConSLIP_712              MGAASMTLTVQARNLLSGIVQQQSNLLRAPEAQQHLLKLTVWGIKQLQARVLAVERYLRD  582
ConSLwIP_712             MGAASMTLTVQARNLLSGIVQQQSNLLRAPEAQQHLLKLTVWGIKQLQARVLAVERYLRD  582
ConSLIPv2_750            MGAASMTLTVQARNLLSGIVQQQSNLLRAPEAQQHLLKLTVWGIKQLQARVLAVERYLRD  582
ConSOSLIP_750            MGAASMTLTVQARNLLSGIVQQQSNLLRAPEAQQHLLQLTVWGIKQLQARVLAVERYLRD  578
ConSOSL_UFO_750          MGAASMTLTVQARNLLSGGSGSG-------------SGSTVWGIKQLQARVLAVERYLRD  565
ConSOSL_UFO_664          MGAASMTLTVQARNLLSGGSGSG-------------SGSTVWGIKQLQARVLAVERYLRD  565
ConSv2_gp160             MGAASMTLTVQARNLLSGIVQQQSNLLRAIEAQQHLLQLTVWGIKQLQARVLAVERYLRD  572
                         ------------------Heptad-Repeat-Helices-1-------------------
                         |         |         |         |         |         |
                        530       540       550       560       570       580      589

BG505_gp160_DQ208458_1   QQLLGIWGCSGKLICTTNVPWNSSWSNRNLSEIWDNMTWLQWDKEISNYTQIIYGLLEES  646
ConS_gp160               QQLLGIWGCSGKLICTTTVPWNSSWSNKSQDEIWDNMTWMEWEREINNYTDIIYSLIEES  641
ConSOSIP_712             QQLLGIWGCSGKLICCTNVPWNSSWSNRNLSEIWDNMTWLQWDKEISNYTQIIYGLLEES  634
ConSOSVD_712             QQLLGIWGCSGKLICCTNVPWNSSWSNRNLSEIWDNMTWLQWDKEISNYTQIIYGLLEES  634
ConSL_712                QQLLGIWGCSGKLICTTNVPWNSSWSNRNLSEIWDNMTWLQWDKEISNYTQIIYGLLEES  642
ConSLVD_712              QQLLGIWGCSGKLICTTNVPWNSSWSNRNLSEIWDNMTWLQWDKEISNYTQIIYGLLEES  642
ConSLIP_712              QQLLGIWGCSGKLICTTNVPWNSSWSNRNLSEIWDNMTWLQWDKEISNYTQIIYGLLEES  642
ConSLwIP_712             QQLLGIWGCSGKLICTTNVPWNSSWSNRNLSEIWDNMTWLQWDKEISNYTQIIYGLLEES  642
ConSLIPv2_750            QQLLGIWGCSGKLICTTNVPWNSSWSNKSQDEIWDNMTWMEWDKEINNYTDIIYSLIEES  642
ConSOSLIP_750            QQLLGIWGCSGKLICCTNVPWNSSWSNKSQDEIWDNMTWMEWDKEINNYTDIIYSLIEES  638
ConSOSL_UFO_750          QQLLGIWGCSGKLICCTNVPWNSSWSNKSQDEIWDNMTWMEWDKEINNYTDIIYSLIEES  625
ConSOSL_UFO_664          QQLLGIWGCSGKLICCTNVPWNSSWSNKSQDEIWDNMTWMEWDKEINNYTDIIYSLIEES  625
ConSv2_gp160             QQLLGIWGCSGKLICTTNVPWNSSWSNKSQDEIWDNMTWMEWDKEINNYTDIIYSLIEES  632
                         -------||CC-Lp||---------------Heptad-Repeat-Helices-2------
                         |         |         |         |         |         |
                        590       600       610       620       630       640      649

BG505_gp160_DQ208458_1   QNQQEKNEQDLLALDKWASLWNWFDISNWLWYIKIFIMIVGGLIGLRIVFAVLSVIHRVR  706
ConS_gp160               QNQQEKNEQELLALDKWASLWNWFDITNWLWYIKIFIMIVGGLIGLRIVFAVLSIVNRVR  701
ConSOSIP_712             QNQQEKNEQDLLALDKWASLWNWFDISNWLWYIKIFIMIVGGLIGLRIVFAVLSVIHRVR  694
ConSOSVD_712             QNQQEKNEQDLLALDKWASLWNWFDISNWLWYIKIFIMIVGGLIGLRIVFAVLSVIHRVR  694
ConSL_712                QNQQEKNEQDLLALDKWASLWNWFDISNWLWYIKIFIMIVGGLIGLRIVFAVLSVIHRVR  702
ConSLVD_712              QNQQEKNEQDLLALDKWASLWNWFDISNWLWYIKIFIMIVGGLIGLRIVFAVLSVIHRVR  702
ConSLIP_712              QNQQEKNEQDLLALDKWASLWNWFDISNWLWYIKIFIMIVGGLIGLRIVFAVLSVIHRVR  702
ConSLwIP_712             QNQQEKNEQDLLALDKWASLWNWFDISNWLWYIKIFIMIVGGLIGLRIVFAVLSVIHRVR  702
ConSLIPv2_750            QNQQEKNEQDLLALDKWASLWNWFDITNWLWYIKIFIMIVGGLIGLRIVFAVLSIVNRVR  702
ConSOSLIP_750            QNQQEKNEQDLLALDKWASLWNWFDITNWLWYIKIFIMIVGGLIGLRIVFAVLSIVNRVR  698
ConSOSL_UFO_750          QNQQEKNEQDLLALDKWASLWNWFDITNWLWYIKIFIMIVGGLIGLRIVFAVLSIVNRVR  685
ConSOSL_UFO_664          QNQQEKNEQDLLALD---------------------------------------------  640
ConSv2_gp160             QNQQEKNEQDLLALDKWASLWNWFDITNWLWYIKIFIMIVGGLIGLRIVFAVLSIVNRVR  692
                         -----------||--------MPER--------||------TM-Domain-----||---
                         |         |         |         |         |         |
                        650       660       670       680       690       700      709
```

```
BG505_gp160_DQ208458_1      QGYSPLSFQTHTPNPRGLDRPERIEEEDGEQDRGRSTRLVSGFLALAWDDLRSLCLFCYH    766
ConS_gp160                  QGYSPLSFQTLIPNPRGPDRPEGIEEEGGEQDRDRSIRLVNGFLALAWDDLRSLCLFSYH    761
ConSOSIP_712                QG----------------------------------------------------------    696
ConSOSVD_712                QG----------------------------------------------------------    696
ConSL_712                   QG----------------------------------------------------------    704
ConSLVD_712                 QG----------------------------------------------------------    704
ConSLIP_712                 QG----------------------------------------------------------    704
ConSLwIP_712                QG----------------------------------------------------------    704
ConSLIPv2_750               QGISPLSFQTLIPNPRGPDRPEGIEEEGGEQDRDRSIRLVN-------------------    743
ConSOSLIP_750               QGISPLSFQTLIPNPRGPDRPEGIEEEGGEQDRDRSIRLVN-------------------    739
ConSOSL_UFO_750             QGISPLSFQTLIPNPRGPDRPEGIEEEGGEQDRDRSIRLVN-------------------    726
ConSOSL_UFO_664             ------------------------------------------------------------    640
ConSv2_gp160                QGYSPLSFQTLIPNPRGPDRPEGIEEEGGEQDRDRSIRLVNGFLALAWDDLRSLCLFSYH    752
                            ------------CT-Domains----------------CT-Domains------------
                            |         |         |         |         |         |        |
                           710       720       730       740       750       760      769

BG505_gp160_DQ208458_1      RLRDFILIAARIVELLGHSSLKGLRLGWEGLKYLWNLLAYWGRELKISAINLFDTIAIAV    826
ConS_gp160                  RLRDFILIAARTVELLGR---KGLRRGWEALKYLWNLLQYWGQELKNSAISLLDTTAIAV    818
ConSOSIP_712                ------------------------------------------------------------    696
ConSOSVD_712                ------------------------------------------------------------    696
ConSL_712                   ------------------------------------------------------------    704
ConSLVD_712                 ------------------------------------------------------------    704
ConSLIP_712                 ------------------------------------------------------------    704
ConSLwIP_712                ------------------------------------------------------------    704
ConSLIPv2_750               ------------------------------------------------------------    743
ConSOSLIP_750               ------------------------------------------------------------    739
ConSOSL_UFO_750             ------------------------------------------------------------    726
ConSOSL_UFO_664             ------------------------------------------------------------    640
ConSv2_gp160                RLRDFILIAARTVELLGR---KGLRRGWEALKYLWNLLQYWGQELKNSAISLLDTTAIAV    809
                            ---------CT-------       ----------------CT-----------------
                            |         |                 |         |         |         |
                           770       780               790       800       810       820

BG505_gp160_DQ208458_1      AEWTDRVIEIGQRLCRAFLHIPRRIRQGLERALL    860
ConS_gp160                  AEGTDRVIEVVQRACRAILNIPRRIRQGLERALL    852
ConSOSIP_712                ----------------------------------    696
ConSOSVD_712                ----------------------------------    696
ConSL_712                   ----------------------------------    704
ConSLVD_712                 ----------------------------------    704
ConSLIP_712                 ----------------------------------    704
ConSLwIP_712                ----------------------------------    704
ConSLIPv2_750               ----------------------------------    743
ConSOSLIP_750               ----------------------------------    739
ConSOSL_UFO_750             ----------------------------------    726
ConSOSL_UFO_664             ----------------------------------    640
ConSv2_gp160                AEGTDRVIEVVQRACRAILNIPRRIRQGLERALL    843
                            ----------------CT---------------|
                                   |         |         |     |
                                  830       840       850   856
```

B. ConSOSL.UFO.750

```
MDRAKLLLLLLLLLLPQAQAVENLWVTVYYGVPVWKDAETTLFCASDAKAYDTEVRNVWATHACVPTDPNPQEIVLENVTENFNMWKNNMV
EQMHTDIISLWDQSLKPCVKLTPLCVTLNCTNVNVTNTTNNTEEKGEIKNCSFNITTELRDKKKKVYALFYRLDVVPIDDNNNNSSNYRLI
NCNTSAITQACPKVSFEPIPIHYCAPAGFAILKCNDKKFNGTGPCKNVSTVQCTHGIKPVVSTQLLLNGSLAEEEIIIRSENITNNAKTII
VQLNESVEINCTRPNNNTRKSIRIGPGQWFYATGDIIGDIRQAHCNISGTKWNKTLQQVVKKLREHFNNKTIIFNPSSGGDLEITTHSFNC
GGEFFYCNTSGLFNSTWIGNGTKNNNNTNDTITLPCRIKQIINMWQRVGQPMYAPPIQGKIRCVSNITGLLLTRDGGNNNTNETETFRPGG
GDMRDNWRSELYKYKVVKIEPLGVAPTRCKRRVVEGGGGSGGGGSAVGIGAVFLGFLGAAGSTMGAASMTLTVQARNLLSGGSGSGSGSTV
WGIKQLQARVLAVERYLRDQQLLGIWGCSGKLICCTNVPWNSSWSNKSQDEIWDNMTWMEWDKEINNYTDIIYSLIEESQNQQEKNEQDLL
ALDKWASLWNWFDITNWLWYIKIFIMIVGGLIGLRIVFAVLSIVNRVRQGISPLSFQTLIPNPRGPDRPEGIEEEGGEQDRDRSIRLVN*
```

Data S1. Sequence alignment and numbering using HXB2 numbering reference. Related to Figure 1. (A) Sequences were aligned using Clustal Omega online tool (EMBL-EBI). HXB2 numbering was attributed according to the lanl database using the HXB2 K03455 reference sequence (https://www.hiv.lanl.gov/content/sequence/HIV/REVIEWS/HXB2.html). Highlighted in grey are the amino acid introduced from BG505 (or specific to BG505), in yellow the amino acid reverted to the ConS gp160 sequence (or specific to ConS gp160) while pink highlighted amino acids indicate stabilization substitutions. The optimized cleavage site RRRRRR is coloured in blue and both cleavage site linker $(SG_4)_2$ and $(G_4S)_2$ are coloured in purple and pink respectively. (**B**) ConSOSL.UFO.750 amino acid sequence.
