# Supplementary material for: Rational Design of DNA-Expressed Stabilized Native-Like HIV-1 Envelope Trimers
Source: Cell Rep. 2018 Sep 18;24(12):3324–3338.e5. doi: 10.1016/j.celrep.2018.08.051 (PMC6167709; doi:10.1016/j.celrep.2018.08.051)
Supplement: Document S2. Article plus Supplemental Information [file mmc2.pdf]

Article

Cell Reports

Rational Design of DNA-Expressed Stabilized Native-Like HIV-1 Envelope Trimers

Graphical Abstract

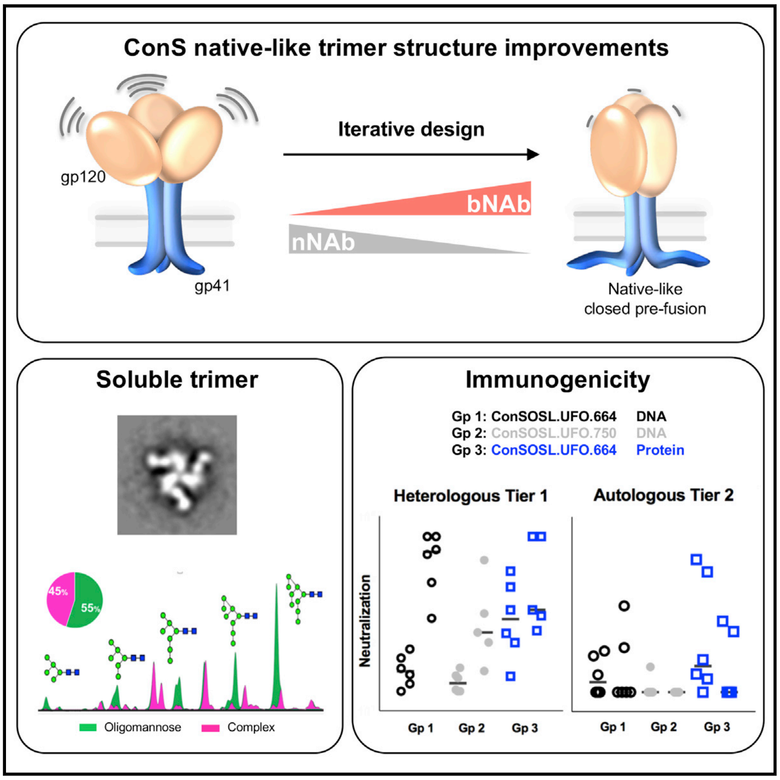

Authors

Yoann Aldon, Paul F. McKay, Joel Allen, ..., Andrew B. Ward, Max Crispin, Robin J. Shattock

Correspondence

r.shattock@imperial.ac.uk

In Brief

Aldon et al. developed membrane-bound and soluble stabilized HIV-1 Env trimer immunogens suitable for DNA/RNA or viral vector vaccines. The sequential iterative design and analysis in muscle cells have the potential to be used as a generalizable method for the expression of stabilized native-like trimers.

Highlights

- DNA-expressed closed pre-fusion native-like Env with preserved MPER exposure
- Env antigenicity varies across cell types and assays
- Muscle cells present properly folded and glycosylated membrane-bound Envs
- Fully glycosylated ConSOSL.UFO.664 induces autologous tier 2 neutralization

Aldon et al., 2018, Cell Reports *24*, 3324–3338
September 18, 2018 © 2018 The Authors.
https://doi.org/10.1016/j.celrep.2018.08.051

CellPress

OPEN
ACCESS
CellPress

Cell Reports
Article

Rational Design of DNA-Expressed Stabilized Native-Like HIV-1 Envelope Trimers

Yoann Aldon,[1] Paul F. McKay,[1] Joel Allen,[2] Gabriel Ozorowski,[3] Réka Felfödiné Lévai,[4] Monica Tolazzi,[5] Paul Rogers,[1] Linling He,[3] Natalia de Val,[3] Katalin Fábián,[4] Gabriella Scarlatti,[5] Jiang Zhu,[3] Andrew B. Ward,[3] Max Crispin,[2,6] and Robin J. Shattock[1,7,*]

[1]Imperial College London, Department of Medicine, Division of Infectious Diseases, Section of Virology, Norfolk Place, London W2 1PG, UK
[2]Oxford Glycobiology Institute, Department of Biochemistry, University of Oxford, Oxford, UK
[3]Department of Integrative Structural and Computational Biology, Collaboration for AIDS Vaccine Discovery, The Scripps Research Institute, La Jolla, CA 92037, USA
[4]Department of Immunology, National Food Chain Safety Office, Directorate of Veterinary Medicinal Products, Budapest, Hungary
[5]Viral Evolution and Transmission Unit, Division of Immunology, Transplantation, and Infectious Diseases, San Raffaele Scientific Institute, Milan, Italy
[6]Department of Immunology and Microbial Science, The Scripps Research Institute, La Jolla, CA 92037, USA
[7]Lead Contact
*Correspondence: r.shattock@imperial.ac.uk
https://doi.org/10.1016/j.celrep.2018.08.051

SUMMARY

The HIV-1-envelope glycoprotein (Env) is the main target of antigen design for antibody-based prophylactic vaccines. The generation of broadly neutralizing antibodies (bNAb) likely requires the appropriate presentation of stabilized trimers preventing exposure of non-neutralizing antibody (nNAb) epitopes. We designed a series of membrane-bound Envs with increased trimer stability through the introduction of key stabilization mutations. We derived a stabilized HIV-1 trimer, ConSOSL.UFO.750, which displays a dramatic reduction in nNAb binding while maintaining high quaternary and MPER-specific bNAb binding. Its soluble counterpart, ConSOSL.UFO.664, displays similar antigenicity, and its native-like Env structure is confirmed by negative stain-EM and glycosylation profiling of the soluble ConSOSL.UFO.664 trimer. A rabbit immunization study demonstrated that the ConSOSL.UFO.664 can induce autologous tier 2 neutralization. We have successfully designed a stabilized native-like Env trimer amenable to nucleic acid or viral vector-based vaccination strategies.

INTRODUCTION

HIV-1-envelope glycoprotein (Env) mediates entry into target cells and is the primary focus of vaccine immunogen design (Burton et al., 2012; Kwong et al., 2011). HIV virions present a restricted number of Env trimers, exposing highly variable outer domain epitopes with tremendous global diversity (Seaman et al., 2010; Zhu et al., 2006). It is likely that any protective vaccine against HIV infection will require the presence of HIV-specific broadly neutralizing antibodies (bNAbs), this hypothesis being strongly supported by the observation that passive infusion of NAbs is protective against challenge in macaques (Moldt et al., 2012).

To date, clinical studies using HIV-1 Env immunogens, mostly gp120 monomeric Env or non-native gp140/gp160 constructs, have failed to generate heterologous NAbs of any clinical significance (Flynn et al., 2005; Moody et al., 2012), instead preferentially recognizing non-neutralizing epitopes. Thus, current design efforts are focused on the generation of stabilized native-like trimers, exposing bNAb epitopes while restricting the presentation of non-neutralizing epitopes occluded on the native functional envelope. This has been greatly enhanced by the detailed characterization of the protein's quaternary structure and fine epitope mapping of the binding sites for bNAbs by crystallography and cryoelectron microscopy (cryo-EM) (Julien et al., 2013a; Kwon et al., 2015; Lyumkis et al., 2013; Sanders et al., 2013).

Efforts to recreate recombinant soluble Env gp140 trimers that mimic native-like conformation of natural functional trimers, where the cytoplasmic tail (CT) and transmembrane regions of gp41 are deleted, are ongoing (de Taeye et al., 2015; Kwon et al., 2015; Schiffner et al., 2015). However, these strategies are generally dependent upon enhanced furin expression in producer cell lines to ensure maximal cleavage of gp140, something that cannot be ensured for nucleic acid or viral vector-based strategies that rely on *in vivo* synthesis of the encoded immunogen. In addition, such approaches require affinity purification to enrich for appropriately folded native-like structures. Furthermore, the presentation of soluble gp140 trimers excludes any potential effects of the lipid bilayer, transmembrane spanning domain, and CT on the conformation and stability of Env (Chen et al., 2015; Dev et al., 2016). Many bNAbs have been shown to bind to closed pre-fusion forms of Env trimers, and recombinant membrane-bound Env showed that tier 2 HIV-1 neutralization could be achieved in a rabbit model (Crooks et al., 2015), suggesting that these forms that may better represent the functional membrane-tethered envelope spike could be engineered to target bNAb germline B cells (Guttman et al., 2015; Steichen et al., 2016). However, there have been limited comparative

 © 2018 The Authors.
This is an open access article under the CC BY license (http://creativecommons.org/licenses/by/4.0/).

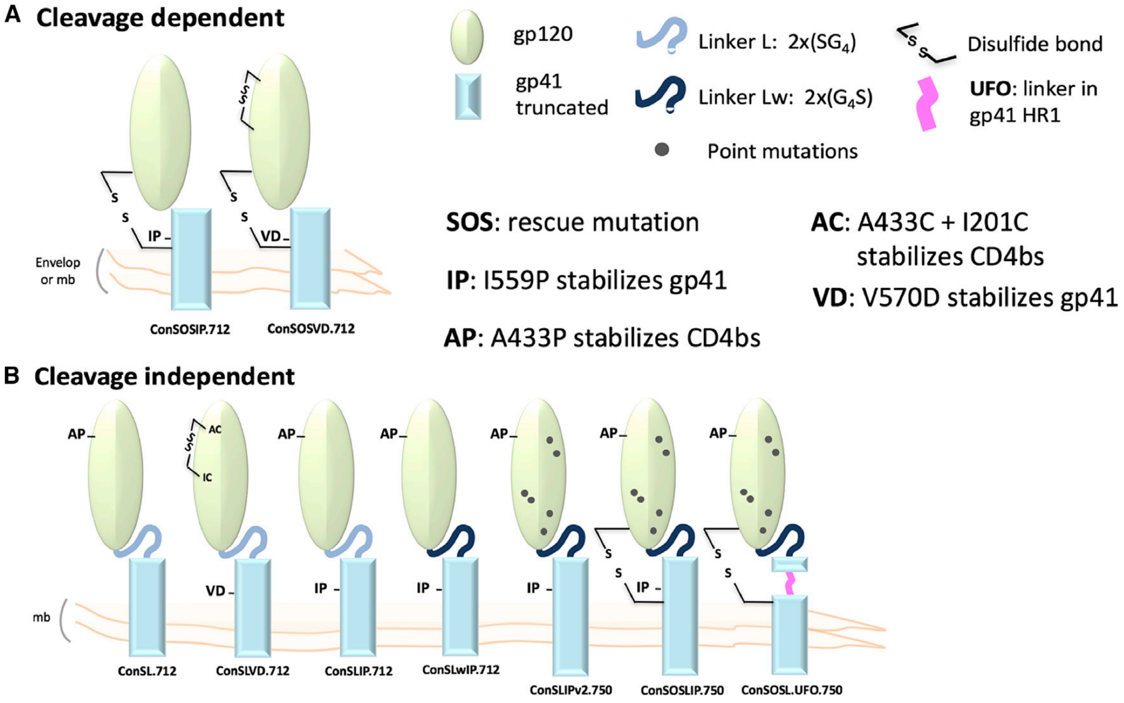

Figure 1. HIV-1 Env Membrane-Bound Stabilized Trimer Designs

Cleavage-dependent (A) and cleavage-independent (B) Env trimers depicted here as gp120-gp41 monomers for clarity. The designs are chronologically organized from left to right at bottom. Features from published gp140 stabilized trimers were introduced as indicated: SOS (Binley et al., 2000) and SOSIP (Sanders et al., 2002); AP and AC+IC (Kwon et al., 2015); VD (Kesavardhana and Varadarajan, 2014); Linker L (Kovacs et al., 2014); Linker Lw (Sharma et al., 2015); UFO: HR1 redesign from uncleaved pre-fusion optimized (Kong et al., 2016). The cytoplasmic tail is truncated at amino acid 712 or 750 (HXB2 numbering). See also Supplemental Data.

studies of the antigenicity and immunogenicity of membrane and soluble stabilized native-like trimers delivered by nucleic acid vaccines.

In the present study, we designed a series of membrane-bound envelopes based on a modified group M consensus Env sequence (ConS), using features from stabilized soluble trimers such as BG505 SOSIP.664 (Kwon et al., 2015; Liao et al., 2006; Sharma et al., 2015; Torrents de la Peña et al., 2017). Through sequential design iterations (Figure 1), we derived the Env ConSOSL.UFO.750 (uncleaved pre-fusion optimized [UFO]), which presents a closed pre-fusion form of membrane-bound Env trimers recognized by bNAbs while masking a number of nNAb epitopes and preserving MPER bNAb epitopes. We compare the antigenicity and immunogenicity of membrane-bound ConSOSL.UFO.750 to its soluble counterpart. The soluble ConSOSL.UFO.664 displayed similar (although not identical) antigenicity, as probed by monoclonal antibody (mAb) binding. Furthermore, analysis by negative stain-EM confirmed the native-like closed conformation of the soluble ConSOSL.UFO.664, and its glycosylation profile was characterized. These data suggest that the derived structural modifications are also sufficient to stabilize the soluble trimer. Immunogenicity studies demonstrated that the soluble form of the ConSOSL.UFO design was better than the membrane-bound version in eliciting autologous tier 2 and heterologous tier 1 neutralization in rabbits. The presented data suggest the ConSOSL.UFO design as an appropriate strategy for nucleic acid or vector-based expression of stabilized native-like HIV-1 envelope trimers.

RESULTS

Cleavage-Independent Design Presents Favorable Quaternary Native-like Features

Our initial studies focused on the modification of membrane-expressed Env based on the ConS sequence (Liao et al., 2006), which was modified to include a number of amino acids from the stabilized soluble BG505 SOSIP.664 trimer (Supplemental Data), including those previously identified to promote trimer stability (Kwon et al., 2015; Torrents de la Peña et al., 2017). Because recycling and endocytosis motifs found in the CT of the full-length gp160 Env led to poor surface expression (Santos da Silva et al., 2013), our first constructs were truncated after residue 712 immediately before the recycling motif YSPL. This truncation dramatically increased Env surface expression levels as shown by flow cytometry (FC) (Figures S1A and S1B).

These features were included in modified ConS Env designs to which an optimized "RRRRRR" efficient furin cleavage motif was introduced (Binley et al., 2002) (Supplemental Data). To stabilize our Env design toward a pre-fusion form, we introduced the SOS mutations (A501C+T605C), which provide a disulfide bond between gp41 and gp120 in combination with stabilizing mutations

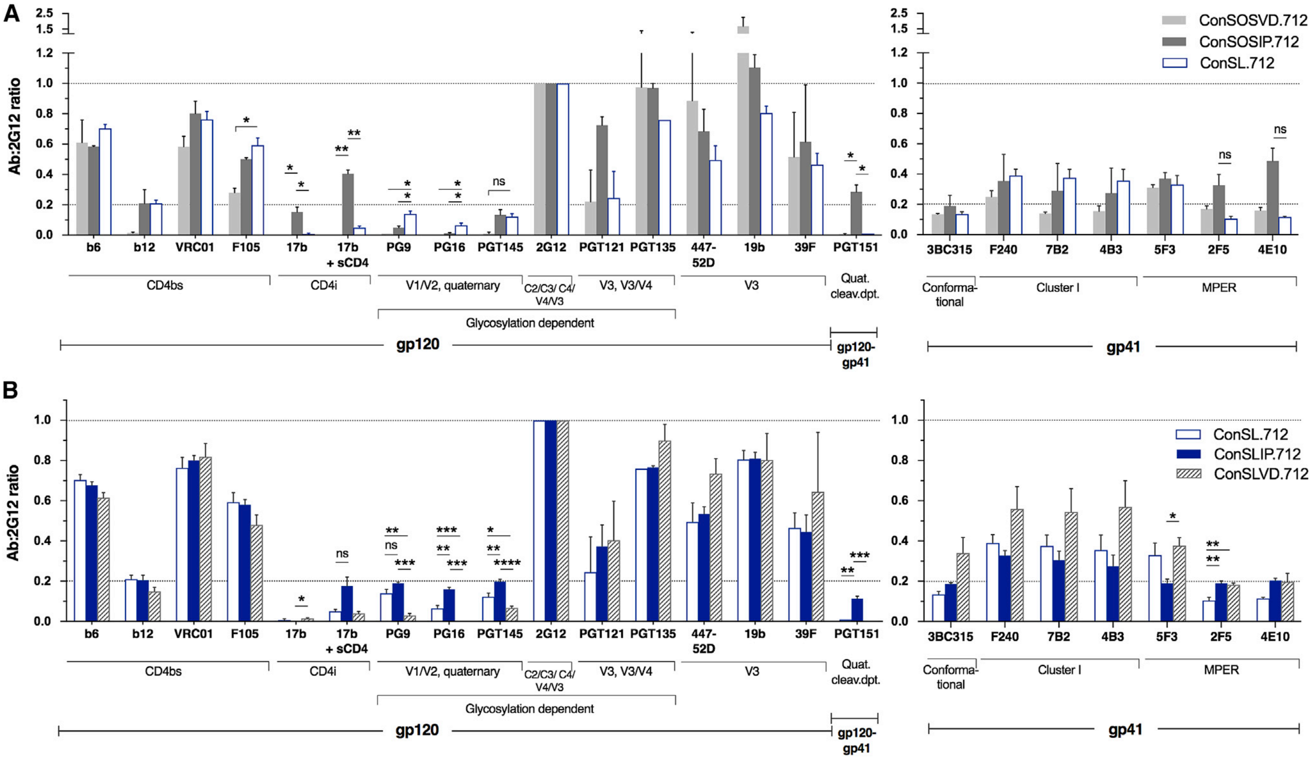

Figure 2. Linker-Stabilized Env Presents a Favorable Quaternary Stabilized Structure

(A) Cleavage-dependent (ConSOSVD.712, ConSOSIP.712) and cleavage-independent (ConSL.712) Envs were tested by flow cytometry for surface epitope exposure in 293T.17 cells.

(B) Stabilization of cleavage-independent Env with I559P mutation (ConSLIP.712) compared to A433C+I201C+V570D (ConSLVD.712). Data are displayed as mean fluorescence intensity (MFI) ratios (mAb:2G12), using 2G12 to normalize the data.

Error bars represent means ± SEMs, with $n \geq 2$ independent experiments. One-way ANOVA with Sidak's multiple comparisons. $^*p < 0.05$, $^{**}p < 0.01$, $^{***}p < 0.001$, ns, not significant. Quat. cleav. dpt., quaternary cleavage dependent. See also Supplemental Data.

in gp41: the I559P (IP) mutation (Binley et al., 2000; Sanders et al., 2002) in ConSOSIP.712 and the V570D (VD) mutation (Kesavardhana and Varadarajan, 2014) in ConSOSVD.712. Additional CD4 binding site (CD4bs) stabilization I201C+A433C mutations forming a disulfide bond to prevent CD4i conformational change (Kwon et al., 2015) were also included in ConSOSVD.712. We compared these cleavage-dependent Envs to a cleavage-independent design, ConSL.712, where the cleavage site between gp120 and gp41 subunits was replaced by a flexible serine-glycine (2x(SG_4)) peptide (Supplemental Data) (Kovacs et al., 2014). This bypasses the need for furin cleavage while preserving the gp120-gp41 association. The ConSL.712 also included the A433P CD4bs mutation as an alternative approach for preventing CD4i conformational change (Figures 1 and 2A) (Kwon et al., 2015).

The ConSL.712 exhibited higher binding of PG9 and PG16 compared to the cleaved Envs and similar binding of PGT145 compared to ConSOSIP.712 (Figure 2A). In addition, the CD4i 17b epitope was barely detectable upon soluble CD4 (sCD4) binding to ConSL.712, suggesting a stable conformational state. This is in contrast to ConSOSIP.712, which does not include the CD4bs stabilization and where the 17b binding was significantly higher both with and without sCD4. Although ConSOSIP.712 seemed to have a more relaxed conformation, the elevated PGT151 binding suggested that the Env precursor is correctly processed with better exposure of membrane proximal external region (MPER) 4E10 and 2F5 epitopes. For ConSOSVD.712 the chosen stabilization mutations clearly disrupted the quaternary structure, with low or no PG9, PG16, PGT145, and PGT151 binding and highly exposed V3 epitopes (447-52D, 19b). The ConSL.712 cleavage-independent design was selected for further improvement on the basis of favorable conformational stability and quaternary epitope features, as indicated by the preferential binding of PG9 and PG16.

Next, we assessed the relative impact of introducing IP (ConSLIP.712) or VD (ConSLVD.712) gp41 mutations into ConSL.712. The IP mutation restored PGT151 binding and increased the quaternary PG9, PG16, and PGT145 binding while reducing gp41 cluster I nnAb epitope exposure relative to the VD mutation (e.g., F240) (Figures 2B and S1C). Although not a direct comparison, because ConSLVD.712 also included the I201C+A433C mutations, we selected ConSLIP.712 for further optimization. We then assessed whether the linker sequence, 2x(SG_4) or 2x(G_4S), could have an impact on trimer folding and stability (Kovacs et al., 2014; Sharma et al., 2015). The ConSLwIP.712 with linker 2x(G_4S) exhibited identical

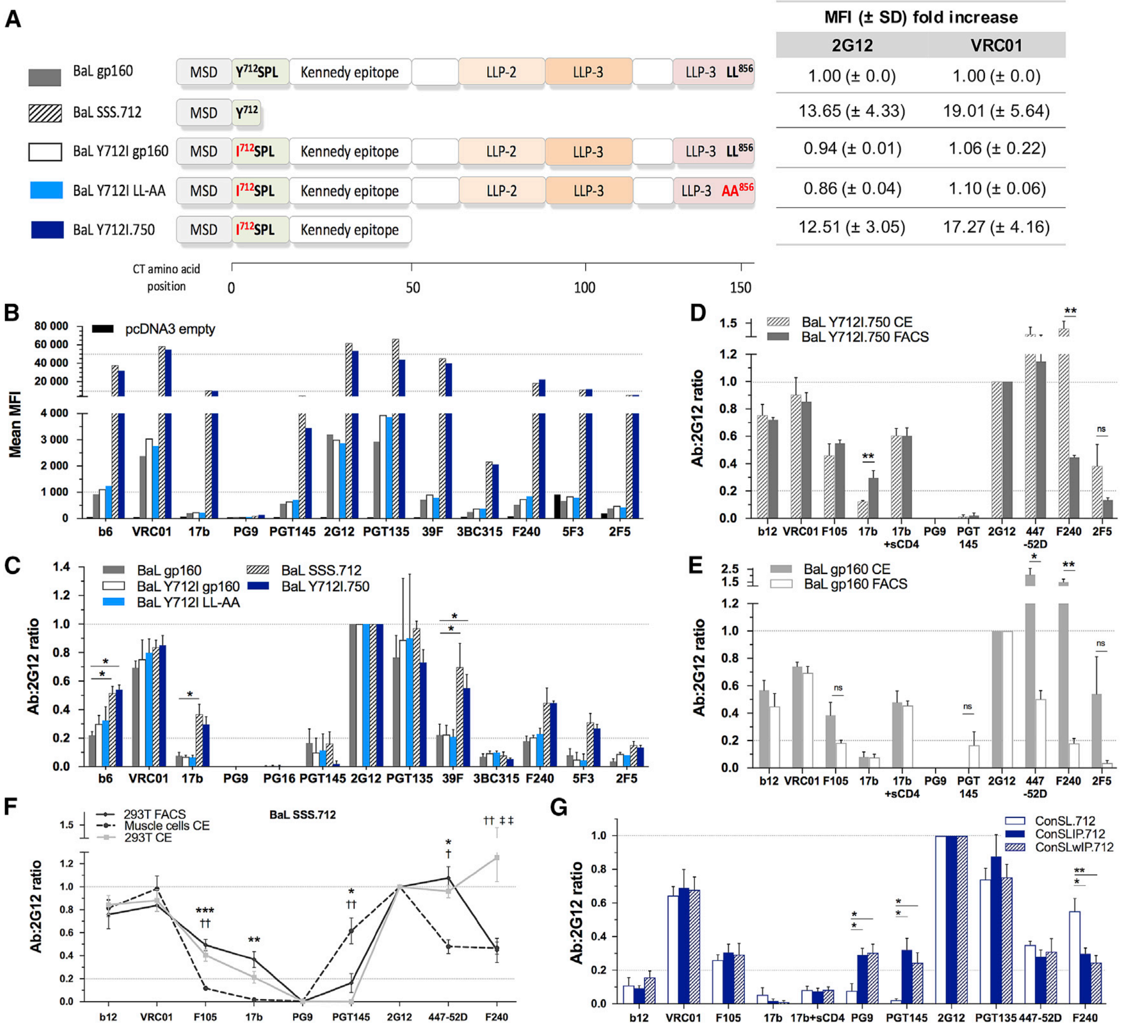

Figure 3. The Adhesion Status, Cellular Host Model, and Cytoplasmic Tail of Env Affect the Epitope Presentation

(A) Comparison of the BaL gp160 and modified BaL Env CT. Mutation of the recycling/endocytosis motifs are indicated in red. The impact on expression levels is reported as mean MFI (± SD) fold increase over BaL gp160 ($n \geq 2$).

(B) Mean MFI values of Env surface expression assessed by FC, representative of $n \geq 2$ independent experiments.

(C) mAb:2G12 ratios as in Figure 2 ($n \geq 2$).

(D and E) Comparison of mAb:2G12 ratios obtained from 293T.17 Cell ELISA (CE) and FC using (D) BaL Y712I.750 and (E) BaL gp160 (n = 3).

(F) Comparison of BaL SSS.712 epitope profile in muscle cell CE, 293T.17 CE, and FC ($n \geq 2$).

(G) Muscle cell CE mAb:2G12 of ConSL.712, ConSLIP.712, and ConSLVD.712 ($n \geq 3$).

For all of the graphs, error bars represent means ± SEMs. One-way ANOVA with Sidak's multiple comparisons. *$p < 0.05$, **$p < 0.01$, ***$p < 0.001$ for (C), (F), and (G), where each sign in (F) compares *muscle cells versus 293T.17 FC, †muscle cells CE versus 293T.17 CE, and ‡293T.17 FC versus 293T CE. Unpaired t test for (D) and (E). FACS, fluorescence-activated cell sorting; LLP, lentiviral lytic peptide domains; MSD, membrane-spanning domain; ns, not significant; SSS, secretion signal sequence. See also Figures S1A, S3, S4, and S5.

binding to the panel of mAbs tested (Figure S1D). These data show favorable quaternary native-like features of the linker-designed Envs, in combination with A433P and IP mutations, over cleavage-dependent Env designs.

The CT, Cellular Model, and Assay Format Impact on the Ectodomain Topology of Env

Previous work demonstrated the impact of mutating or truncating the CT on the ectodomain topology of Env (Chen et al., 2015). To conserve most of the full-length sequence of Env and preserve its native epitope display, we mutated known recycling motifs and truncated the CT after the Kennedy epitope (Bhakta et al., 2011; Byland et al., 2007). To facilitate comparison with previous studies, we tested four constructs based on the HIV-1 BaL Env, comparing them to the wild-type BaL gp160 (Figure 3A). Mutation of the conserved YxxØ motif (hydrophobic residue [Ø]) ^{712}YSPL^{715} to ^{712}ISPL^{715}, either alone (BaL Y712I gp160) or in association with the C-terminal dileucine ^{855}LL^{856}

mutation to AA (BaL Y712I LL-AA), did not lead to an increase in cell surface expression levels. This suggests that other endocytosis or recycling motifs such as ^{768}YHRL^{771} or the synergy of partially functional endocytosis motifs are sufficient to limit surface presentation of Env (Figures 3A, 3B, and S2).

In comparison, truncation at residue 750, together with the Y712I mutation (BaL Y712I.750), showed high surface expression levels comparable to BaL SSS.712 (secretion signal sequence [SSS]). However, 750 and 712 versions displayed higher 17b and V3 epitope exposure (447-52D, 39F) compared to BaL gp160 (Figures 3 and S2). Furthermore, there was a non-significant increase in F240, 5F3, and 2F5 binding. PG9 and PG16 binding was not observed because the BaL gp160 sequence lacks an essential glycosylation site in position 160. Other Abs presented similar binding ratios (e.g., VRC01, PGT145, 3BC315). The truncation at 750 highlighted the fact that a longer CT could be incorporated in our design, including the post-attachment neutralizing Kennedy epitope (Steckbeck et al., 2013), having the same impact on ectodomain epitopes as a truncation at 712.

Because we are interested in DNA vaccination approaches using the intramuscular route, we sought to evaluate the effect of the cellular model and assays on the Ab-binding profile of Env. We hypothesized that Env may display different Ab-binding profiles when analyzed in suspension by FC compared to direct analysis of adherent cells ("On Cell" ELISA [CE]). Based on published work, we optimized a CE, allowing us to examine Ab binding to surface expressed Env on adherent cells (Figures S3A–S3C) (Veillette et al., 2014). Significant changes in Ab-binding profiles were observed with BaL Y712I.750 or BaL gp160 across the two assays (Figures 3D and 3E). BaL Y712I.750 showed higher 17b epitope exposure in FC and higher F240 binding by CE, whereas BaL gp160 exhibited significantly higher 447-52D and F240 binding by CE but no change in 17b binding. Thus, the observed changes in Env antigenicity depend both on the sequence and the adherence status of the cells.

We next explored the use of human primary skeletal muscle cells as a model for membrane-expressed antigen as characterized by CE (Figure S3D). We were surprised to find that the Env Ab-binding profile of transfected muscle cells differed from 293T.17 by CE and FC (Figure 3F). Binding of nNAbs F105, 17b, and 447-52D was significantly reduced compared to 293T.17 cells. In contrast, binding of the PGT145 bNAb was significantly increased in muscle cells. These data suggest that muscle cell presentation of Env influences ectodomain structure and resultant epitope presentation. We demonstrate here that Env antigenicity varies not only between assays but also between cell types; the mechanism underpinning such differences merits further exploration.

Subsequently, we evaluated ConSL.712, ConSLIP.712, and ConSLwIP.712 in the muscle cell expression model (Figure 3G). Binding of PGT145 and PG9 proved to be higher in muscle cell CE for ConSLIP and ConSLwIP, but lower for ConSL compared to FC (Figures 2B and 3G). As observed for BaL SSS.712, a reduction of F105 and 447-52D binding was noted for the three constructs. However, F240 nNAb binding to ConSL.712 was increased in muscle cell CE compared to FC, and F240 binding to ConSL.712 was also significantly higher than ConSLIP.712 ($p < 0.05$) and ConSLwIP.712 ($p < 0.01$) in muscle cells. ConSLwIP.712 was selected for further design improvements.

Enhancement of Quaternary bNAb Epitope Affinity

Although most of the essential residues for quaternary structure- and glycan-dependent bNAb binding (e.g., PG9, PGT145, PGT121) are found in ConSLwIP.712 (Julien et al., 2013b; McLellan et al., 2011), binding of these Abs was relatively low compared to 2G12 or VRC01. To determine whether additional improvements could be made in the binding of quaternary Abs, we reverted any amino acid substitutions introduced into ConSLwIP.712 from BG505 that had not been identified as essential in previous studies (Kwon et al., 2015; Torrents de la Peña et al., 2017). In addition, we extended the CT to 750 to include the Kennedy epitope (Supplemental Data). Cytometry experiments revealed that the revertant Env ConSLIPv2.750 exhibited enhanced binding of PG9, PG16, PGT145, PGT121, b12, PGT151, and 35O22 bNAbs compared to ConSLwIP.712 (Figures 4A, S4A, and S4B). Moreover, even though important nNAb contact residues were left unchanged in ConSLIPv2.750 (e.g., K305, I307, S306, and I309 for 39F; ^{312}GPGQ^{315} for 447-52D) (Pantophlet et al., 2008), the degree of binding of these Abs to the target protein was reduced. A tryptophan at position A316W, thought to stabilize the V3 loop in AMC008 and BG505 SOSIP.664 trimers (de Taeye et al., 2015), was introduced in ConSLIPv2.750 with the aim of stabilizing the V3. However, because 19b binding remained high, we probed our constructs with the following additional V3-specific mAbs: 10-1074, 2191, 2219, 3074, and 3869. These mAbs showed that the V3 remains highly accessible, while the A316W mutation only perturbs the binding of 447-52D and 39F. Although binding of quaternary bNAbs was increased, gp41 cluster I, cluster II, and MPER epitopes were clearly more exposed, with significantly higher binding of F240, 7B2, 4B3, 98-6D, 167-D, and 5F3 compared to ConSLwIP.712 (Figures 4B, S4A, and S4B). More important, 17b and E51 CD4i Abs, which recognize distinct epitopes upon sCD4 binding, did not bind, indicating that ConSLIPv2.750 is unable to undergo CD4i conformational change. This was observed for both muscle cells and 293T.17 when assessed by FC and CE (Figures 4A, 4D, 4E, and S4C). In contrast, the matching gp160 (ConSv2 gp160) did efficiently undergo sCD4i conformational change, as shown by the high binding of 17b (Figure 4E). Despite the high binding of quaternary specific bNAbs, including PGT145, which recognizes solely native-like closed trimers, no conformational changes upon sCD4 binding, and high VRC-CH31 binding, we observed a greatly enhanced b12 signal in ConSLIPv2.750. This suggests that although the trimer has the features of a native-like trimer, transiently and partially open conformations of the trimer can be sampled and stabilized by b12 (Guttman et al., 2015; Li et al., 2011; Liu et al., 2008; Meyerson et al., 2013; Pugach et al., 2015). However, decreased binding of CD4bs nNAbs F105 and b6 are indicative of a structural improvement toward a more native-like conformation with functional epitope features (Figure 4) (Chen et al., 2009; Stieh et al., 2015). The reduction in VRC01 binding likely reflects differences in the V5 loop of ConSLIPv2.750 compared to ConSLwIP.712, with, respectively, two versus one N-linked glycans, which may

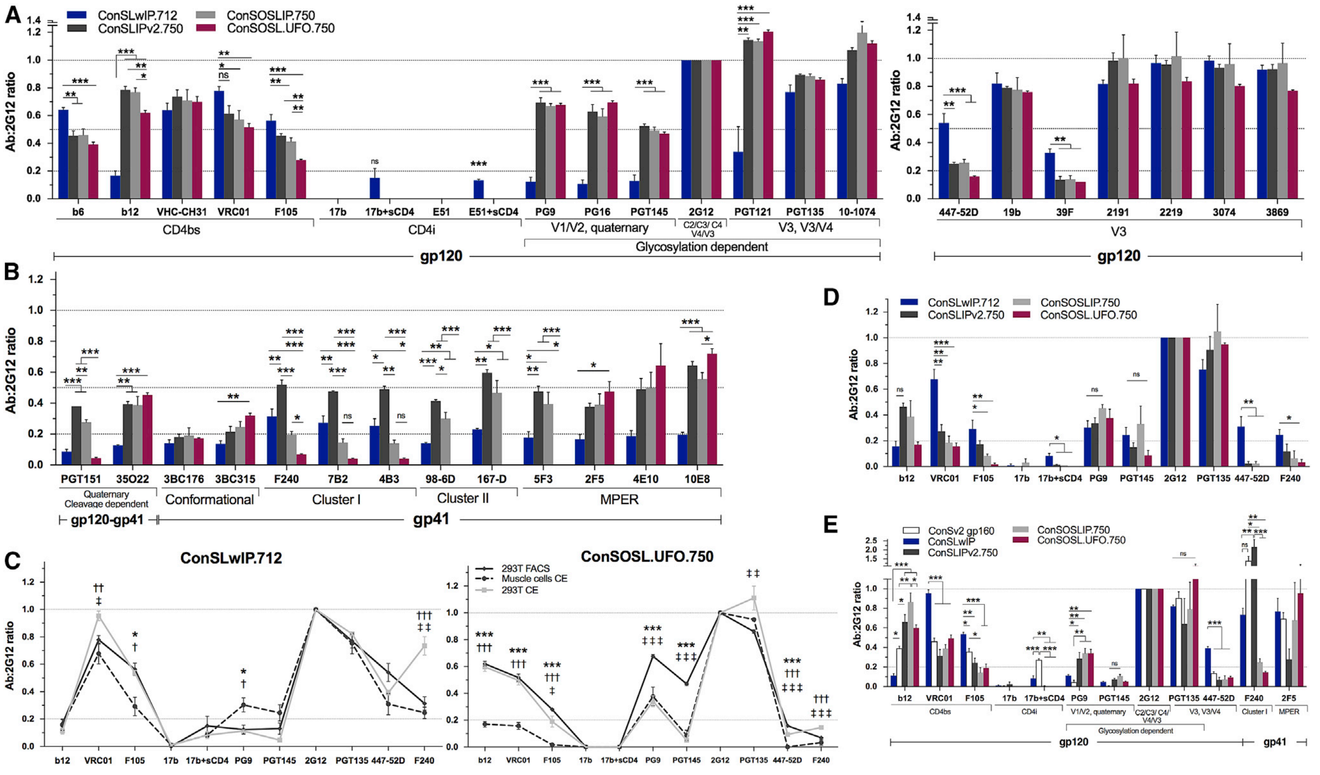

Figure 4. Enhancement of bNAb Binding and Subsequent Reduction of nNAb Binding by gp41 HR1 Stabilization

(A) mAb:2G12 binding profile as in Figure 2, comparing ConSLwIP.712 to subsequent stabilized constructs for gp120-specific mAbs ($n \geq 2$, ConSOSL.UFO.750, $n \geq 3$).

(B) Same as in (A) for gp41 and gp120-gp41 interface-specific mAbs.

(C) Comparison of ConSLwIP.712 and ConSOSL.UFO.750 epitope profiles in muscle cell CE, 293T.17 CE, and FC ($n \geq 2$).

(D) Muscle cell CE mAb:2G12 ($n \geq 3$, ConSOSLIP.750 PGT135, n = 2).

(E) mAb:2G12 ratios obtained from 293T.17 CE ($n \geq 3$, except PGT135, $n \geq 2$).

For all of the graphs, error bars represent means ± SEMs. One-way ANOVA with Sidak's multiple comparisons. *$p < 0.05$, **$p < 0.01$, ***$p < 0.001$, where each sign in (C) compares *muscle cells versus 293T.17 FC, †muscle cells CE versus 293T.17 CE, and ‡293T.17 FC versus 293T CE. ns, not significant. See also Figures S4 and S5.

limit the accommodation of the heavy and light chains around the V5 loop (Figure 4; Supplemental Data) (Li et al., 2011; Zhou et al., 2010, 2015). This hypothesis is supported by the high binding of the VRC01-class, VRC-CH31 bNAb, because it targets the CD4bs with a similar angle of approach to VRC01 but is not affected by the changes in ConSLIPv2.750.

gp41 and CD4bs nNAb Epitope Masking Using SOS and UFO Stabilization Features

We next evaluated whether the addition of the SOS mutations to ConSLIPv2.750 could further stabilize the trimer, thereby decreasing nNAb epitope exposure. The introduction of SOS in ConSOSLIP.750 noticeably improved the nNAb-binding profile, with >2-fold reduction in gp41 F240, 7B2, and 4B3 nNAb binding. However, the gp41 cluster II (98-6D, 167-D) and MPER 5F3 epitopes remained highly accessible (Figures 4B, 4D, and S4A–S4C). Binding of bNAbs was maintained to the same levels, except for a slight reduction for PGT151. Binding of PG9 and PGT145 was higher in muscle cells for ConSOSLIP.750 as compared to ConSLIPv2.750 and F105 binding was lower, although it did not reach statistical significance (Figure 4D). For both constructs, 447-52D binding significantly dropped to almost zero for muscle cells and 293T.17 CE. The lower b12 and VRC01 binding in muscle cells suggests a tighter conformation of the trimers constricting the CD4bs in these cells.

We then explored whether additional benefit could be provided through the introduction of a short serine-glycine linker $(GS)_4$ in the metastable gp41 HR1 loop (positions 548–568) derived from soluble UFO stabilized trimers (Kong et al., 2016). ConSOSL.UFO.750 was derived through the incorporation of the HR1 linker, eliminating the IP mutation and generating a gp41 HR1 that is 13 amino acids shorter than wild-type. This modification of the HR1 further reduced the binding of nNAbs F240, 7B2, and 4B3, confirmed across the three assays (FC, 293T.17 CE, and muscle cell CE) (Figure 4). Moreover, gp41 cluster II epitope binding by nNAbs 98-6D and 167-D and the MPER nNAb 5F3 were completely abolished in ConSOSL.UFO.750, while the gp41 conformation-specific bNAb 3BC315 binding increased relative to ConSLwIP.712 (Figures 4B and S5B). This suggests that ConSOSL.UFO.750 presents a majority of trimeric forms of Env on the cell surface in a pre-fusion state, because monomers and dimers would expose gp41 nNAb epitopes,

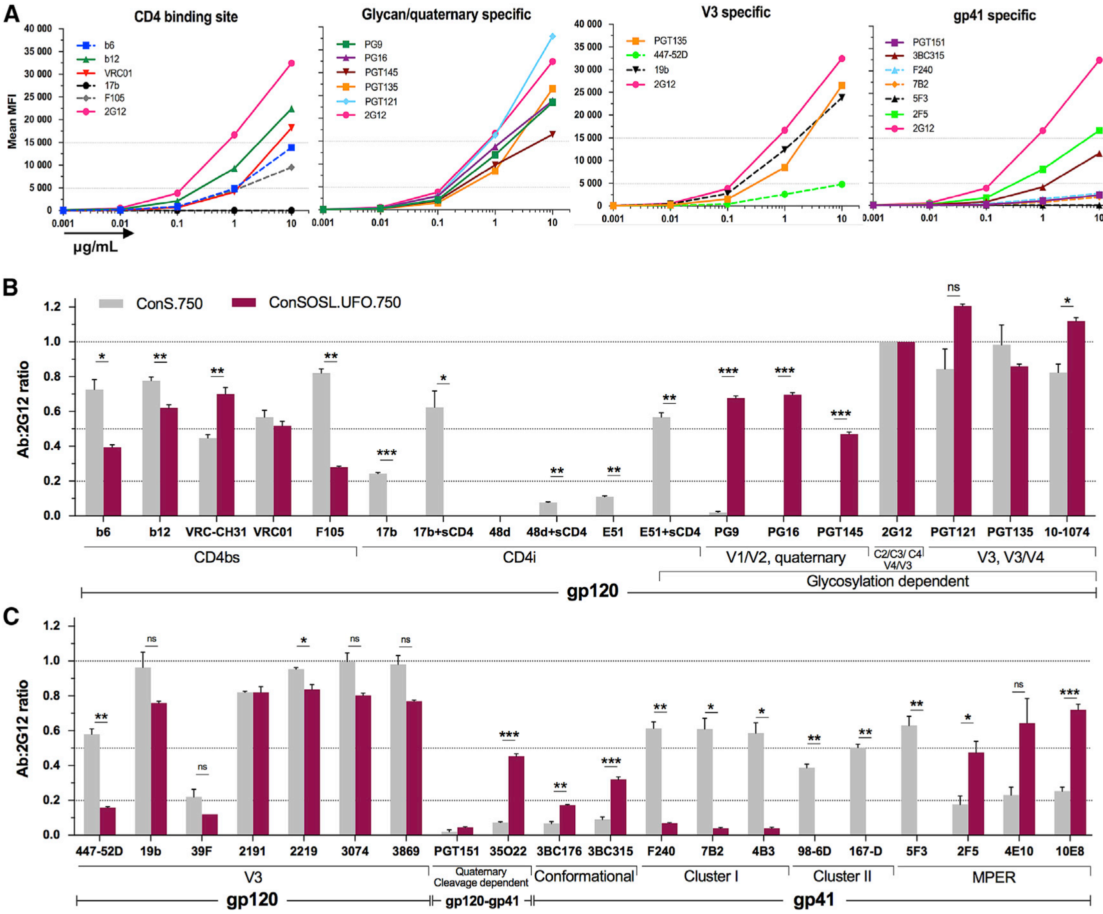

Figure 5. BNAbs Bind More Efficiently to ConSOSL.UFO.750 than nNAbs

(A) Mean MFI values of ConSOSL.UFO.750 surface expression assessed by FC using a panel of mAbs. These Abs were titrated starting at 10 μg/mL in a 10-fold dilution series. Titration curves grouped by Env domain specificity. 2G12 plotted on all graphs for comparison. Solid lines, bNAbs; dotted lines, nNAbs. Representative of n = 2 independent experiments.

(B) mAb:2G12 binding profile as in Figure 2, comparing ConS.750 for a panel of 15 mAbs (original ConS sequence truncated at position 750) to ConSOSL.UFO.750 (n ≥ 2; ConSOSL.UFO.750, n ≥ 3).

(C) Same as in (B) for an additional 20 mAbs.

For (B) and (C), error bars represent means ± SEMs. Unpaired t test. *p < 0.05, **p < 0.01, ***p < 0.001. ns, not significant.

See also Figure S6A.

and 98-6D and 167-D are known to tightly bind Env in its post-fusion state (Frey et al., 2010). It also suggests that the gp120 subunits are sitting down on gp41 and are masking these epitopes. In contrast, the high binding of PG9 and PGT145 was preserved and detected in the three assays, with lower binding observed in muscle cells and 293T.17 CE compared to FC, confirming the impact of cellular adherence on Env antigenicity (Figure 4). Although the binding levels of VRC01 and VRC-CH31 were maintained, we observed a reduction in b12 and a further reduction in b6 and F105 binding—close to zero for F105 in muscle cells. The binding of MPER bNAbs 2F5, 4E10, and 10E8 was retained as well as the binding of PGT135 and 2G12 mannose patch-specific bNAbs (Figure 4). The V3 remained exposed despite a minor decrease in binding for some mAbs. The observed reduction in binding of PGT151 to ConSOSL.UFO.750 relative to ConSOSLIP.750 indicates that the gp41 $(GS)_4$ linker interferes with the PGT151 epitope, likely modifying the contact region reached by the HCDR3 and formed between the HR1 regions of two gp41 protomers and the fusion peptide (Blattner et al., 2014; Kong et al., 2016; Lee et al., 2016). This hypothesis is supported by the binding data of the quaternary gp120-gp41 interface-specific bNAb 35O22, which showed strong and higher binding for ConSOSL.UFO.750 than for ConSOSLIP.750; this confirmed that the gp120-gp41 quaternary interface is conserved (Figures 4 and 6E). These results indicate that ConSOSL.UFO.750 presents a stabilized closed native-like conformation with a favored exposure of bNAb epitopes.

bNAbs Efficiently Bind to ConSOSL.UFO.750

We further evaluated the affinity of mAbs to ConSOSL.UFO.750 by titrating a panel of mAbs using FC (Figures 5A and S6A). Results showed no binding for 17b and a rapid drop for the nNAbs

b6, F105, 447-52D, F240, 7B2, and 4B3. However, 19b displayed strong affinity for the V3 loop. High binding at low concentration was observed for all of the bNAbs tested except PGT151, as expected.

To evaluate improvements made in relation to the original consensus sequence, we compared ConSOSL.UFO.750 to the consensus Env truncated at position 750 (ConS.750) (Figures 5B and S4A). We observed that binding of nNAbs b6 and F105 was greatly reduced, while binding of VRC-CH31 was increased and VRC01 maintained, indicating that the structural integrity of the CD4bs is preserved and favored bNAb binding over nNAb. CD4i mAbs 17b, 48d, and E51 did not bind to ConSOSL.UFO.750, while they did bind to ConS.750 without sCD4 (17b, E51) and upon sCD4 engagement. Quaternary and glycan-dependent bNAbs PG9, PG16, PGT145, and 35O22, which are low or absent in ConS.750, were dramatically increased in ConSOSL.UFO.750, demonstrating that the designed Env presents a more closed trimeric conformation and allows binding of these structure-dependent bNAbs. Glycosylation-dependent bNAbs such as 2G12, PGT121, PGT135, and 10-1074 were maintained or increased in ConSOSL.UFO.750. Although a modest decrease in binding was observed for 447-52D, 19b, 39F, 2219, 3074, and 3869 compared to ConS.750, the V3 of ConSOSL.UFO.750 remained exposed. gp41 nNAb binding was considerably higher in ConS.750, while very low or absent in ConSOSL.UFO.750. Moreover, the conformational gp41-specific bNAbs 3BC176 and 3BC315 were increased. Finally, binding of the MPER bNAbs 2F5, 4E10, and 10E8 was increased for ConSOSL.UFO.750, whereas 5F3 nNAb was abrogated. Together with the titration results, the improvements made to ConSOSL.UFO.750 in comparison to ConS.750 support our previous observations that ConSOSL.UFO.750 presents fewer nNAb epitopes and favors bNAb epitopes within a prefusion native-like Env conformation.

Soluble gp140 ConSOSL.UFO.664

To compare and contrast the structure and glycosylation of ConSOSL.UFO.750, we produced its soluble version as ConSOSL.UFO.664. The size exclusion chromatography (SEC) profile of ConSOSL.UFO.664 showed that the principal form of gp140 produced was trimeric (trimer peak ~54%), with a high yield achieved in 293T.17 cells and confirmed by native gels (Figures 6A, 6B, and S6B). Thus, the suggested trimeric form of ConSOSL.UFO.750 is supported by this soluble gp140 SEC profile.

After a second SEC purification of the trimer peak fractions (Figures S6C–S6E), both site-specific and whole glycoprotein glycan analyses were performed on trimeric ConSOSL.UFO.664 (Figures 6C and S6F). These analyses showed that ConSOSL.UFO.664 trimers present a fairly even distribution between oligomannose and complex-type glycans, representing 55% and 45% of the glycans, respectively.

Site-specific analysis of ConSOSL.UFO.664 revealed that both the intrinsic mannose patch (IMP) and the trimer-associated mannose patch (TAMP), observed on other Envs, are largely conserved. Sites such as N156 and N160 on the apex of the Env glycoprotein present a high proportion of oligomannose-type glycans and form the TAMP (Crispin and Doores, 2015). The glycans at several sites that constitute the IMP, including N262, N295, N332, and N448 on the ectodomain of gp120, predominantly present $Man_9GlcNAc_2$ (Figure 6D) (Pritchard et al., 2015a; Sok et al., 2016). The N-linked glycan at position N339 is more processed than for BG505, with a large population of hybrid-type glycans detected. ConSOSL.UFO.664 contains a proline in position 363, and therefore the N362 site, which is not a conserved site but is present in BG505, is not glycosylated. Site-specific analysis on BG505 SOSIP.664 revealed the proximity of N363 to the N339 site, and the absence of the N362 site in ConSOSL.UFO.664 could result in increased accessibility of the N339 glycan by mannosidases. Although N234 and N276 contain predominantly oligomannose-type structures, some complex glycans are found at these positions, indicating that this region of the C2 domain is partially accessible to α-mannosidases (Behrens et al., 2016; Doores et al., 2010; Pritchard et al., 2015a). The composition of the glycan at N156 is predominantly $Man_9GlcNAc_2$, showing that this site is shielded from enzymatic processing. The N160 glycan on the V2 loop is found in the vicinity of the V1 N156 glycan and displays mostly $Man_9GlcNAc_2$. This contrasts with BG505 SOSIP.664, which presents more processed N160 glycans (Behrens et al., 2016). The presence of glycan N130 in the vicinity of N160, which is lacking in BG505, could protect N160 from processing. Another N-linked glycan site at the apex of the trimer, N197, is significantly less processed than on BG505, with no complex glycans detected. This observation could suggest that the V1/V2 loops of the apex of ConSOSL.UFO.664 are more compact than on BG505 (Crispin and Doores, 2015).

The sites that were processed to the greatest extent on the gp120 subunit were N88, N130, N138, N190, N356, N406, and N464 (Figure 6D), as observed on BG505 SOSIP.664 (Behrens et al., 2016; Stewart-Jones et al., 2016). The glycans in the ectodomain of gp41, N611, N616, and N637 were comparable to a published BG505 SOSIP.664 gp41 site analysis (Behrens et al., 2016), and, similar to BG505, the N625 site was not detected.

To further assess the antigenicity of soluble ConSOSL.UFO.664, we developed a capture ELISA that allows presentation of trimers in the native-like conformation using a cMyc-HIS tag. Titration of mAbs demonstrated preferential binding of bNAbs over nNAbs (Figures 6E and 6F). PG9 and PG16 binding was similar to that of ConSOSL.UFO.750, whereas PGT145 binding was strongly reduced for the captured ConSOSL.UFO.664. Although PGT145 is able to neutralize viruses presenting Env with high $Man_9GlcNAc_2$ content, the reduction in PGT145 binding suggests that the epitope on the V2 apex is less accessible (McLellan et al., 2011; Walker et al., 2011). No conformational changes were induced upon sCD4 binding (17b, E51). Furthermore, VRC01, b12, and b6 had similar binding curves with good affinity to the trimer, while F105 bound poorly. In contrast to gp41 nNAbs, which bound poorly (F240) or not at all (4B3, 5F3, 98-6D, 167-D), the quaternary specific bNAb 3BC315 showed high binding to the trimer. Similar to the membrane-bound Env, the V3-specific 447-52D and 39F did not bind to the trimer, while 19b, 3074, and 10-1074 exhibited binding, confirming V3 exposure. Again, comparable to ConSOSL.UFO.750, bNAb PGT151 showed limited binding to ConSOSL.UFO.664, whereas 35O22 proved to have a high affinity for the soluble

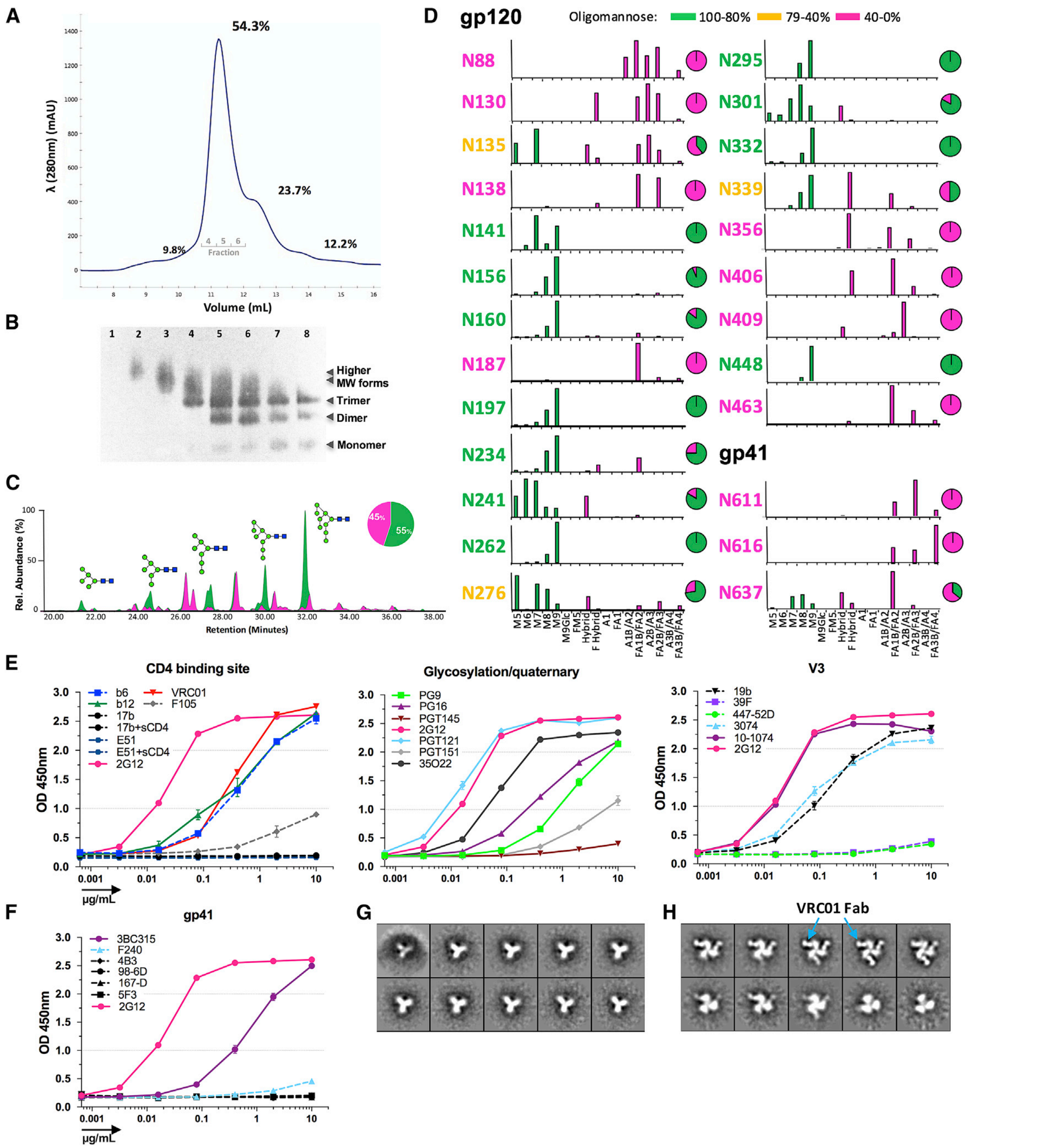

Figure 6. ConSOSL.UFO.664 Env Trimers Present a Native-Like Pre-fusion Conformation

(A) Size exclusion chromatography (SEC) profile of the ConSOSL.UFO.664 produced in 293T.17 cells.

(B) Native PAGE-western blotting of SEC fractions from (A), detected with 2G12.

(C) Hydrophilic interaction-ultra-performance liquid chromatography (HILIC-UPLC) profile of N-linked glycans from SEC purified ConSOSL.UFO.664 trimers. Oligomannose-type (green) and complex-type (pink) glycans were identified by endoglycosidase H (Endo H) digestion. Rel., relative.

(D) Relative N-glycosylation sites.

(E) mAbs titration against ConSOSL.UFO.664 Myc-HIS trimers captured by anti-cMyc 9E10 Ab. Representative of at least n = 2 independent experiments. The titration curves are grouped by Env domain specificity, and 2G12 is plotted on all of the graphs for comparison. Solid lines, bNAbs; dotted lines, nNAbs. OD, optical density.

(F) Same as in (E) for gp41-specific mAbs.

(G and H) Negative stain-electron microscopy (NS-EM) images showing views of (G) the propeller shape of the ligand-free trimer and (H) in complex with VRC01 Fab.

See also Figures S6B–S6I.

trimer. These data demonstrate that the soluble ConSOSL.UFO.664 trimer presents a pre-fusion native-like antigenic profile, with a more closed conformation limiting the N160 glycan processing by mannosidases and access to the PGT145 epitope.

Structural Analysis

We next assessed the ConSOSL.UFO.664 structure by negative stain-EM (NS-EM). Two-dimensional (2D) classification shows that 100% of the trimers presented a native-like structural propeller shape (Figure 6G). Incubation of ConSOSL.UFO.664 with molar excess of VRC01 antigen-binding fragment (Fab) reveals that a majority of trimers bound two to three Fab molecules, while the presence of a subpopulation of ligand-free trimers is consistent with the decrease in VRC01 binding measured by other assays, possibly attributed to differences in V5 sequence (Figure 6H). NanoDSF (differential scanning fluorimetry) thermal stability analysis demonstrates a melting profile that is similar to other well-behaved SOSIP constructs (e.g., BG505, B41) and a melting transition point of 58.5°C, roughly 3°C higher and −8°C lower than B41 and BG505, respectively (Figure S6G). In addition, the trimer was incubated at both 4°C and 37°C during an 8-week period, showing that the protein is stable and maintains structural integrity (Figures S6H and S6I).

Immunogenicity of the ConSOSL.UFO Design

We subsequently investigated the potential of the ConSOSL.UFO design to induce a humoral response in mice, guinea pigs, and rabbits. Mice and guinea pigs were injected with either ConSOSL.UFO.750 or ConSOSL.UFO.664 DNA with electroporation. After the second immunization, the ConSOSL.UFO.750 induced higher specific immunoglobulin G (IgG) than ConSOSL.UFO.664 in both species (Figures S7A and S7B). The isotype analyses of mice sera revealed that the membrane-bound Env triggered a more balanced T helper response and even a Th1 skewed response in some animals, while the soluble Env induced a dominant Th2 response (Figure S7C). This demonstrates that the membrane-bound and soluble Env delivered as DNA induces distinct T cell responses.

Because the Env delivered as naked DNA was immunogenic but induced low specific IgG titers in guinea pigs, we sought to evaluate the immunogenicity of ConSOSL.UFO in a DNA prime-protein boost study in rabbits following a similar schedule to that previously associated with the elicitation of tier 2 autologous NAb (Chakrabarti et al., 2013). Rabbits were immunized with 100 μg DNA at weeks 0, 4, and 8, and then boosted at week 20 with 25 μg ConSOSL.UFO.664 trimer with AddaVax adjuvant (DNA/DNA/DNA/protein immunization [DDDP]); one group was injected at weeks 4, 8, and 20 with protein plus adjuvant (PPP) (Figure 7A). Here, ConSOSL.UFO.664 induced a higher titer than the membrane-bound Env early in the DDDP scheme; however, both groups reached similar titers after the third immunization. The PPP group reached a high titer after two immunizations at 339 μg/mL. At week 22, 2 weeks after the final protein boost, all of the groups were efficiently boosted by ∼1–1.5 log compared to week 20.

We then determined the proportion of the V3-specific IgG at weeks 10 and 22. Competitive ELISAs using V3 peptides revealed that membrane-bound and soluble immunogens delivered as DNA induced the same levels of V3 response in both guinea pigs and rabbits (Figures 7B, S7D, and S7E). In addition, no difference in V3 response was observed between weeks 10 and 22, indicating that the protein boost did not skew the response toward V3 (Figure 7B).

Finally, we assessed the neutralization potency of the rabbit sera on a small panel of viruses. Both DDDP groups showed heterologous tier 1 neutralization against clade C MW965.26 virus at week 10 with a median infectious dose 50 (ID_{50}) (50% inhibition in TZM-bl assay) <50, while the PPP group had a median ID_{50} of 257 (Figure 7C). At week 22, the median ID_{50} of ConSOSL.UFO.750 and 664 DDDP groups increased to 161 and 2,817. As expected, neutralization against MW965.26 proved to be V3 mediated, as shown by V3 competitive neutralization data (Figure S7F). The three groups did not neutralize tier 1 clade B MN virus, and neutralization of tier 1 SF162 was limited. Tier 1 ConM virus was the most neutralized by sera from PPP at week 22. Despite the absence of glycan holes across the 21 conserved glycans of ConSOSL.UFO (McCoy et al., 2016), the 664 DDDP and PPP groups were able to induce autologous tier 2 neutralization at weeks 10 and 22 (Figure 7C). The DNA-induced NAb response was slightly increased in two rabbits after the protein boost for 664 DDDP, while neutralization titers decreased in the PPP ConS group between weeks 10 and 22. V3 competition showed that neutralization against ConS was partially directed to V3 (Figure S7F). Except for one rabbit at week 10, no autologous tier 2 neutralization was detected in the ConSOSL.UFO.750 group. It should be noted that the ConS virus used for neutralization has a 4% amino acid difference (excluding linkers and leader sequence) compared to ConSOSL.UFO. No heterologous tier 2 neutralization against TV1.29 was observed (Figure 7C).

DISCUSSION

The stabilized BG505 SOSIP.664 soluble trimer has been critical in resolving the structural topography of bNAb bound to native-like trimers using crystallography and EM (Huang et al., 2014; Julien et al., 2013a; Kwon et al., 2015; Sanders et al., 2013). However, a requirement for high furin expression to ensure maximal cleavage of gp140, combined with the necessity for Ab-based affinity purification to select appropriately folded trimers, means that this process is not amenable to nucleic acid (DNA/RNA) or viral vector strategies, in which the encoded transgene is generally targeted for expression by transduced muscle cells. To overcome these potential shortcomings, we developed a broadly applicable design for the expression of cleavage-independent stabilized membrane expressed and soluble trimers. Using a group M consensus sequence (Liao et al., 2006) as our starting point, we assessed the impact of sequential design iterations using features from stabilized soluble trimers such as BG505 SOSIP.664 (Figure 1). We derived the ConSOSL.UFO.750 Env that presents a closed pre-fusion form of membrane-bound Env trimers recognized by bNAbs, while masking a number of nNAb epitopes. To exploit any potential increase in trimer stability, orientation, and avidity afforded by membrane expression, we included the transmembrane region and truncated CT. The use of a cleavage-independent flexible

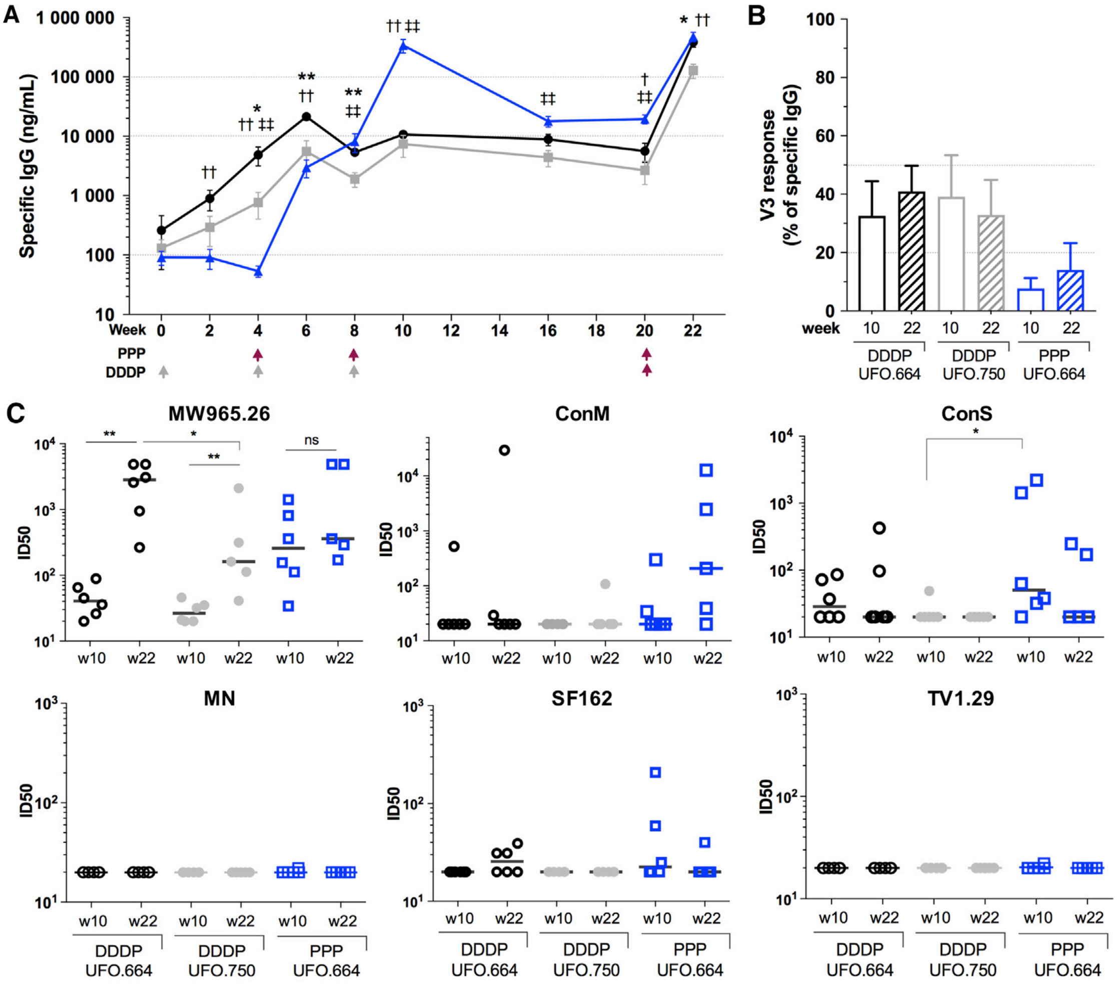

Figure 7. Immunogenicity of ConSOSL.UFO Design in Rabbits

(A) Antigen-specific serum IgG binding assessed by 9E10 capture ELISA for DNA immunized rabbits with ConSOSL.UFO.750 plasmid (gray) and ConSOSL.UFO.664 plasmid (black) both boosted with protein (DDDP) and for ConSOSL.UFO.664 protein alone (blue) (PPP). ConSOSL.UFO.664 Myc-HIS trimers were used as captured antigen, and IgG concentration determined using a captured standard rabbit IgG. Gray arrows, DNA IM+EP immunization; red arrows, protein immunization.

(B) Proportion of V3 response as assessed by competitive ELISA using matching V3 cys-cyclized peptide.

(C) Neutralization of HIV-1 pseudoviruses in TZM-bl assay by rabbit sera from weeks 10 and 22. Data plotted as ID_{50} values (serum dilution that inhibits by 50% infectivity).

MW965.26, MN, SF162, and ConM are tier 1 viruses; ConS and TV1.29 are tier 2. n = 6 animals per group, except for groups ConSOSL.UFO.750 DDDP and 664 PPP at week 22, where n = 5. Error bars represent means ± SEMs. Unpaired t test. *$p < 0.05$, **$p < 0.01$. For (A), *750 DDDP versus 664 DDDP; †664 PPP versus 664 DDDP; ‡664 PPP versus 750 DDDP. DDDP, DNA/DNA/DNA/protein immunization; PPP, protein/protein/protein. See also Figures S7A–S7F.

linker between gp120-gp41 homodimers removed the requirement for high levels of endogenous furin (Kovacs et al., 2014), while the inclusion of a short linker in the HR1 (Kong et al., 2016) provided further stabilization, restricting accessibility of nNAb epitopes in gp41. The introduction of the stabilization mutations taken from SOSIPv4.2 trimers H66R+A316W (de Taeye et al., 2015), including the M535+N543 from SOSIPv3, led to a reduced binding of some V3 nNAb epitopes (447-52D, 39F), but it was not sufficient to bury the V3 loop. This may be achieved by additional modifications like those incorporated into BG505 SOSIP MD39 (Steichen et al., 2016). Nevertheless, recent studies have shown that lower V3 responses do not enhance the elicitation of autologous or heterologous tier 2 neutralization (Kulp et al., 2017; Pauthner et al., 2017). Our incorporation of the A433P CD4bs-stabilizing mutation (Kwon et al., 2015), together with the H66R mutation, prevented CD4i conformational change and associated binding of CD4i nNAbs (e.g., 17b, 48d, E51). Although this was associated with a small decrease in the binding of CD4bs Abs (VRC01, b6, and F105), binding of b12 mAb increased, the latter suggesting some flexibility of the trimer to accommodate b12 (Li et al., 2011; Liu et al., 2008). Important improvements in the quaternary structure of ConSOSL.UFO.750 Env led to enhanced binding of PG9, PG16, PGT145, and 35O22, which is reflective of a native-like structure. Moreover,

incorporation of the gp41 HR1 linker stabilization occluded gp41 cluster I and completely masked cluster II epitopes also associated with a closed native-like conformation. Despite this apparent closed conformation, the trimers still bound MPER-specific bNAbs. This conflicts with a previous study suggesting that binding of MPER-specific bNAbs was associated with a more open conformation on viral particles (Chakrabarti et al., 2011). However, the interaction of the Env CT with viral matrix within viral particles may apply additional positioning effects on the exposure of MPER eptitopes and thus differ from cell membrane-expressed trimers. SOSIP stabilization of JR-FL gp160, when used in conjunction with an optimized cleavage strategy, increased both PG16 and PG9 binding and MPER exposure (Kesavardhana and Varadarajan, 2014). In this respect, the stabilization mutations appear to accommodate exposure of the MPER along with quaternary closed native-like features, while occluding gp41 cluster I and II nNAb epitopes.

Because our design strategy is intended for nucleic acid or viral vector delivery via the intramuscular route, we compared the antigenicity of Envs expressed in human primary skeletal muscle cells to 293T.17 assessed by FC and CE. Significant variations in antigenicity were observed between suspension cells in FC and adherent cells by CE, as well as between cell types (293T.17 versus muscle cells). nNAb F105, 17b, 447-52D, and F240 epitopes were generally less accessible when presented on muscle cells compared to 293T.17 across all of the tested constructs (Figures 3F, 4C, S4, and S5). Here, we show that muscle cells produce Envs that present glycosylation- and quaternary-dependent epitopes (PG9, PGT145, 2G12, PGT135). These data highlight the importance of assessing antigenicity in target cells for *in vivo* transduction.

To determine whether the ConSOSL.UFO design provided sufficient stabilization in the absence of membrane expression, we also produced a soluble version of the trimer and investigated its antigenicity and structure. The SEC profile of the soluble gp140 ConSOSL.UFO.664 indicated a predominance of trimers (Figures 6A, 6B, and S6B–S6E). Furthermore, the ELISA Ab-binding profile was very similar to that observed for ConSOSL.UFO.750 by FC, except for PGT145. The low PGT145 binding to soluble ConSOSL.UFO.664 contrasts with the high binding observed for ConSOSL.UFO.750 by FC and highlights the influence of the membrane context on Env antigenicity (Figures 5 and 6E). The BG505 SOSIP.664 trimer, which binds PGT145 with high affinity, presents processed N160 and N197 glycans with a limited proportion of $Man_9GlcNAc_2$ and lacks the N130 glycan (Behrens et al., 2016). By contrast, N130, N160, and N197 on ConSOSL.UFO.664 are dominated by $Man_9GlcNAc_2$, followed by $Man_8GlcNAc_2$ oligomannose forms. These glycan forms have been shown to inhibit PGT145 binding to the BG505 SOSIP trimer when produced in the presence of kifunensine, a treatment that generates Env with a uniform $Man_{8–9}GlcNAc_2$ glycan composition (Figure 6D) (Lee et al., 2017). It is unclear whether the differences in binding of PGT145 to soluble and membrane-bound forms of ConSOSL.UFO (Figures 5, 6D, and 6E) reflect differences in conformation or glycosylation. The former is supported by previous observations showing that PGT145 can still neutralize HIV pseudovirions produced in cells treated with kifunensine (Walker et al., 2011). However, the latter is supported by prior studies reporting differences in the glycosylation profile of membrane-bound and soluble Env (Go et al., 2015). These two findings may not be mutually exclusive, because differences in conformation likely influence the resultant glycan processing.

Site-specific glycan analysis of ConSOSL.UFO.664 showed that oligomannose forms, particularly $Man_9GlcNAc_2$, predominate on the trimer apex N156, N160, and N197 glycans (Figure 6D). This provides additional evidence of a tightly closed quaternary conformation protecting the TAMP formed by these three glycans and preserving the IMP. Previous studies have suggested that binding of PG9 and PG16 is dependent on complex glycan structures or $Man_5GlcNAc_2$ (McLellan et al., 2011; Pancera et al., 2013). This contrasts a more recent report that while the binding of PG9 and PG16 to BG505 SOSIP.664 is not dependent upon complex glycans or $Man_5GlcNAc_2$, the affinity of these bNAbs may be impeded by the presence of $Man_9GlcNAc_2$ in position N160 (Behrens et al., 2016). These previous assumptions are challenged by the high binding of PG9 and PG16 to ConSOSL.UFO.664, given the lack of $Man_5GlcNAc_2$ and predominant $Man_9GlcNAc_2$ at position N160. Our findings suggest that PG9 and PG16 have a more resilient binding capacity to a variety of oligomannose and complex glycans than previously thought. Further evidence for the native-like structure of ConSOSL.UFO.664 is provided by NS-EM, demonstrating a uniform closed pre-fusion native-like structure (Figures 7A and 7B) that proved to be stable with a high melting point temperature (Figure S6G).

Because both membrane and soluble forms of the ConSOSL.UFO design (750 and 664) presented favorable characteristics of native-like trimers, we compared their immunogenicity in animal models. In mice and guinea pigs, ConSOSL.UFO.750 induced higher Ab titers compared to ConSOSL.UFO.664 after two DNA immunizations, while the opposite was observed in rabbits (Figures 7A, S7A, and 7B). Membrane-bound and soluble DNA-delivered antigens triggered distinct T helper responses in mice (Figure S7C). While it is beyond the scope of the present study, it would be interesting to determine whether potential differences in T helper responses between mice and rabbits may account for differences in the kinetics of the Ab responses to membrane-tethered and soluble trimers.

Rabbit immunogenicity studies demonstrated that recombinant ConSOSL.UFO.664 protein efficiently boosted DNA primed responses. Autologous tier 2 neutralization was predominantly observed for ConSOSL.UFO.664 at week 10, following three DNA or two protein immunizations, with greater responses in the protein-only group. These data were somewhat surprising, given that membrane-expressed trimers may provide a more authentic presentation of native-like trimers. The greater responses in the protein-only group likely reflect differences in titer between protein-only and DNA regimens. However, while autologous neutralization was observed in the protein-only group at week 10 after two immunizations, these responses were reduced or absent at week 22 following a third immunization at week 20, suggesting either a shift in epitope specificity or immunodominance of non-neutralizing epitopes.

OPEN
ACCESS
CellPress

V3 competition showed that autologous neutralization was only partially directed to V3, which suggests that other epitopes likely contribute to the observed neutralization. Furthermore, there was no difference in magnitude of V3 responses between weeks 10 and 22. Unlike BG505 SOSIP, ConSOSL.UFO presents all of the conserved N-linked glycan sites (Klasse et al., 2016; McCoy et al., 2016; Torrents de la Peña et al., 2017) (Figure 7C); thus, the observed neutralization is not dependent upon the recognition of isolate-specific glycan holes. Determining the epitope specificity of these transient autologous neutralizing responses may be key to developing strategies to selectively boost and maintain them.

In summary, our data suggest that the ConSOSL.UFO design developed in this study would provide a suitable approach for DNA/RNA or vector-based immunogens. The fully glycosylated ConSOSL.UFO.664 trimer induced autologous tier 2 neutralization after DNA or protein immunizations and appeared to induce better responses than the membrane-tethered version. The derived design principles have the potential to be used as a generalizable method for the expression of stabilized native-like trimers for multiple HIV Envs when expressed from nucleic acid or viral vectors. Given the high cost of manufacture of recombinant proteins, the use of nucleic acid vaccines offers a cost-effective approach to assess multiple Env design strategies in experimental medicine phase I clinical trials. Furthermore, such a design strategy may be critical to appropriate priming of B cells in heterologous prime-boost regimes that combine nucleic acid and/or viral vector approaches with recombinant protein boosts.

STAR★METHODS

Detailed methods are provided in the online version of this paper and include the following:

- KEY RESOURCES TABLE
- CONTACT FOR REAGENT AND RESOURCE SHARING
- EXPERIMENTAL MODEL AND SUBJECT DETAILS
  - Cells
  - Animals
- METHOD DETAILS
  - Env and sCD4 proteins production
  - HIV-1 monoclonal Abs and 9E10
  - Cell surface-binding assays
  - Blue Native Western Blotting
  - Glycan analysis by HILIC-UPLC
  - Assigning glycan compositions using tandem ion mobility ESI MS
  - Site-specific N-glycosylation analysis
  - Negative-stain electron microscopy and NanoDSF
  - Animals and immunization
  - Antigen specific IgG capture ELISA
  - Neutralization assays
- QUANTIFICATION AND STATISTICAL ANALYSIS

SUPPLEMENTAL INFORMATION

Supplemental Information includes supplemental data and seven figures and can be found with this article online at https://doi.org/10.1016/j.celrep.2018.08.051.

ACKNOWLEDGMENTS

We thank Nathan P. Sweeney for advice on cultivating primary human muscle cells and Christine A. Bricault for discussions on trimer purification. This project has received funding from the European Union's Horizon 2020 for Research & Innovation program under grant agreement No. 681137. The sole responsibility for the content of this project lies with the authors. It does not necessarily reflect the opinion of the European Union. The European Commission is not responsible for any use that may be made of the information contained therein. The majority of this research was carried out at Imperial College London (London, UK). Electron microscopy imaging was carried out at The Scripps Research Institute in San Diego, CA, USA, and funded by the Bill and Melinda Gates Foundation Collaboration for AIDS Vaccine Discovery (OPP1115782) and the glycosylation work carried out at the Oxford Glycobiology Institute (Oxford, UK).

AUTHOR CONTRIBUTIONS

Conceptualization, R.J.S., Y.A., and P.F.M.; Methodology, R.J.S., Y.A., P.F.M., and J.Z.; Investigation, Y.A., P.F.M., J.A., G.O., R.F.L., M.T., P.R., K.F., L.H., and N.d.V.; Writing – Original Draft, Y.A.; Review & Editing, P.F.M., R.J.S., J.A., G.O., G.S., M.C., and A.B.W.

DECLARATION OF INTERESTS

The authors declare no competing interests.

Received: May 15, 2017
Revised: May 16, 2018
Accepted: August 17, 2018
Published: September 18, 2018

REFERENCES

Behrens, A.J., Vasiljevic, S., Pritchard, L.K., Harvey, D.J., Andev, R.S., Krumm, S.A., Struwe, W.B., Cupo, A., Kumar, A., Zitzmann, N., et al. (2016). Composition and antigenic effects of individual glycan sites of a trimeric HIV-1 envelope glycoprotein. Cell Rep. *14*, 2695–2706.

Bhakta, S.J., Shang, L., Prince, J.L., Claiborne, D.T., and Hunter, E. (2011). Mutagenesis of tyrosine and di-leucine motifs in the HIV-1 envelope cytoplasmic domain results in a loss of Env-mediated fusion and infectivity. Retrovirology *8*, 37.

Binley, J.M., Sanders, R.W., Clas, B., Schuelke, N., Master, A., Guo, Y., Kajumo, F., Anselma, D.J., Maddon, P.J., Olson, W.C., and Moore, J.P. (2000). A recombinant human immunodeficiency virus type 1 envelope glycoprotein complex stabilized by an intermolecular disulfide bond between the gp120 and gp41 subunits is an antigenic mimic of the trimeric virion-associated structure. J. Virol. *74*, 627–643.

Binley, J.M., Sanders, R.W., Master, A., Cayanan, C.S., Wiley, C.L., Schiffner, L., Travis, B., Kuhmann, S., Burton, D.R., Hu, S.L., et al. (2002). Enhancing the proteolytic maturation of human immunodeficiency virus type 1 envelope glycoproteins. J. Virol. *76*, 2606–2616.

Blattner, C., Lee, J.H., Sliepen, K., Derking, R., Falkowska, E., de la Peña, A.T., Cupo, A., Julien, J.P., van Gils, M., Lee, P.S., et al. (2014). Structural delineation of a quaternary, cleavage-dependent epitope at the gp41-gp120 interface on intact HIV-1 Env trimers. Immunity *40*, 669–680.

Burton, D.R., Ahmed, R., Barouch, D.H., Butera, S.T., Crotty, S., Godzik, A., Kaufmann, D.E., McElrath, M.J., Nussenzweig, M.C., Pulendran, B., et al. (2012). A blueprint for HIV vaccine discovery. Cell Host Microbe *12*, 396–407.

Byland, R., Vance, P.J., Hoxie, J.A., and Marsh, M. (2007). A conserved dileucine motif mediates clathrin and AP-2-dependent endocytosis of the HIV-1 envelope protein. Mol. Biol. Cell *18*, 414–425.

Chakrabarti, B.K., Walker, L.M., Guenaga, J.F., Ghobbeh, A., Poignard, P., Burton, D.R., and Wyatt, R.T. (2011). Direct antibody access to the HIV-1

membrane-proximal external region positively correlates with neutralization sensitivity. J. Virol. *85*, 8217–8226.

Chakrabarti, B.K., Feng, Y., Sharma, S.K., McKee, K., Karlsson Hedestam, G.B., Labranche, C.C., Montefiori, D.C., Mascola, J.R., and Wyatt, R.T. (2013). Robust neutralizing antibodies elicited by HIV-1 JRFL envelope glycoprotein trimers in nonhuman primates. J. Virol. *87*, 13239–13251.

Chen, L., Kwon, Y.D., Zhou, T., Wu, X., O'Dell, S., Cavacini, L., Hessell, A.J., Pancera, M., Tang, M., Xu, L., et al. (2009). Structural basis of immune evasion at the site of CD4 attachment on HIV-1 gp120. Science *326*, 1123–1127.

Chen, J., Kovacs, J.M., Peng, H., Rits-Volloch, S., Lu, J., Park, D., Zablowsky, E., Seaman, M.S., and Chen, B. (2015). HIV-1 ENVELOPE. Effect of the cytoplasmic domain on antigenic characteristics of HIV-1 envelope glycoprotein. Science *349*, 191–195.

Crispin, M., and Doores, K.J. (2015). Targeting host-derived glycans on enveloped viruses for antibody-based vaccine design. Curr. Opin. Virol. *11*, 63–69.

Crooks, E.T., Tong, T., Chakrabarti, B., Narayan, K., Georgiev, I.S., Menis, S., Huang, X., Kulp, D., Osawa, K., Muranaka, J., et al. (2015). Vaccine-elicited tier 2 HIV-1 neutralizing antibodies bind to quaternary epitopes involving glycan-deficient patches proximal to the CD4 binding site. PLoS Pathog. *11*, e1004932.

de Taeye, S.W., Ozorowski, G., Torrents de la Peña, A., Guttman, M., Julien, J.P., van den Kerkhof, T.L., Burger, J.A., Pritchard, L.K., Pugach, P., Yasmeen, A., et al. (2015). Immunogenicity of stabilized HIV-1 envelope trimers with reduced exposure of non-neutralizing epitopes. Cell *163*, 1702–1715.

Dev, J., Park, D., Fu, Q., Chen, J., Ha, H.J., Ghantous, F., Herrmann, T., Chang, W., Liu, Z., Frey, G., et al. (2016). Structural basis for membrane anchoring of HIV-1 envelope spike. Science *353*, 172–175.

Doores, K.J., Bonomelli, C., Harvey, D.J., Vasiljevic, S., Dwek, R.A., Burton, D.R., Crispin, M., and Scanlan, C.N. (2010). Envelope glycans of immunodeficiency virions are almost entirely oligomannose antigens. Proc. Natl. Acad. Sci. USA *107*, 13800–13805.

Flynn, N.M., Forthal, D.N., Harro, C.D., Judson, F.N., Mayer, K.H., and Para, M.F.; rgp120 HIV Vaccine Study Group (2005). Placebo-controlled phase 3 trial of a recombinant glycoprotein 120 vaccine to prevent HIV-1 infection. J. Infect. Dis. *191*, 654–665.

Frey, G., Chen, J., Rits-Volloch, S., Freeman, M.M., Zolla-Pazner, S., and Chen, B. (2010). Distinct conformational states of HIV-1 gp41 are recognized by neutralizing and non-neutralizing antibodies. Nat. Struct. Mol. Biol. *17*, 1486–1491.

Go, E.P., Herschhorn, A., Gu, C., Castillo-Menendez, L., Zhang, S., Mao, Y., Chen, H., Ding, H., Wakefield, J.K., Hua, D., et al. (2015). Comparative analysis of the glycosylation profiles of membrane-anchored HIV-1 envelope glycoprotein trimers and soluble gp140. J. Virol. *89*, 8245–8257.

Guttman, M., Cupo, A., Julien, J.P., Sanders, R.W., Wilson, I.A., Moore, J.P., and Lee, K.K. (2015). Antibody potency relates to the ability to recognize the closed, pre-fusion form of HIV Env. Nat. Commun. *6*, 6144.

Harvey, D.J. (2005a). Fragmentation of negative ions from carbohydrates: part 1. Use of nitrate and other anionic adducts for the production of negative ion electrospray spectra from N-linked carbohydrates. J. Am. Soc. Mass Spectrom. *16*, 622–630.

Harvey, D.J. (2005b). Fragmentation of negative ions from carbohydrates: part 2. Fragmentation of high-mannose N-linked glycans. J. Am. Soc. Mass Spectrom. *16*, 631–646.

Harvey, D.J. (2005c). Fragmentation of negative ions from carbohydrates: part 3. Fragmentation of hybrid and complex N-linked glycans. J. Am. Soc. Mass Spectrom. *16*, 647–659.

Harvey, D.J., Royle, L., Radcliffe, C.M., Rudd, P.M., and Dwek, R.A. (2008). Structural and quantitative analysis of N-linked glycans by matrix-assisted laser desorption ionization and negative ion nanospray mass spectrometry. Anal. Biochem. *376*, 44–60.

Huang, J., Kang, B.H., Pancera, M., Lee, J.H., Tong, T., Feng, Y., Imamichi, H., Georgiev, I.S., Chuang, G.Y., Druz, A., et al. (2014). Broad and potent HIV-1 neutralization by a human antibody that binds the gp41-gp120 interface. Nature *515*, 138–142.

Julien, J.P., Cupo, A., Sok, D., Stanfield, R.L., Lyumkis, D., Deller, M.C., Klasse, P.J., Burton, D.R., Sanders, R.W., Moore, J.P., et al. (2013a). Crystal structure of a soluble cleaved HIV-1 envelope trimer. Science *342*, 1477–1483.

Julien, J.P., Sok, D., Khayat, R., Lee, J.H., Doores, K.J., Walker, L.M., Ramos, A., Diwanji, D.C., Pejchal, R., Cupo, A., et al. (2013b). Broadly neutralizing antibody PGT121 allosterically modulates CD4 binding via recognition of the HIV-1 gp120 V3 base and multiple surrounding glycans. PLoS Pathog. *9*, e1003342.

Kesavardhana, S., and Varadarajan, R. (2014). Stabilizing the native trimer of HIV-1 Env by destabilizing the heterodimeric interface of the gp41 postfusion six-helix bundle. J. Virol. *88*, 9590–9604.

Klasse, P.J., LaBranche, C.C., Ketas, T.J., Ozorowski, G., Cupo, A., Pugach, P., Ringe, R.P., Golabek, M., van Gils, M.J., Guttman, M., et al. (2016). Sequential and simultaneous immunization of rabbits with HIV-1 envelope glycoprotein SOSIP.664 trimers from clades A, B and C. PLoS Pathog. *12*, e1005864.

Kong, L., He, L., de Val, N., Vora, N., Morris, C.D., Azadnia, P., Sok, D., Zhou, B., Burton, D.R., Ward, A.B., et al. (2016). Uncleaved prefusion-optimized gp140 trimers derived from analysis of HIV-1 envelope metastability. Nat. Commun. *7*, 12040.

Kovacs, J.M., Noeldeke, E., Ha, H.J., Peng, H., Rits-Volloch, S., Harrison, S.C., and Chen, B. (2014). Stable, uncleaved HIV-1 envelope glycoprotein gp140 forms a tightly folded trimer with a native-like structure. Proc. Natl. Acad. Sci. USA *111*, 18542–18547.

Kulp, D.W., Steichen, J.M., Pauthner, M., Hu, X., Schiffner, T., Liguori, A., Cottrell, C.A., Havenar-Daughton, C., Ozorowski, G., Georgeson, E., et al. (2017). Structure-based design of native-like HIV-1 envelope trimers to silence non-neutralizing epitopes and eliminate CD4 binding. Nat. Commun. *8*, 1655.

Kwon, Y.D., Pancera, M., Acharya, P., Georgiev, I.S., Crooks, E.T., Gorman, J., Joyce, M.G., Guttman, M., Ma, X., Narpala, S., et al. (2015). Crystal structure, conformational fixation and entry-related interactions of mature ligand-free HIV-1 Env. Nat. Struct. Mol. Biol. *22*, 522–531.

Kwong, P.D., Mascola, J.R., and Nabel, G.J. (2011). Rational design of vaccines to elicit broadly neutralizing antibodies to HIV-1. Cold Spring Harb. Perspect. Med. *1*, a007278.

Lee, J.H., Ozorowski, G., and Ward, A.B. (2016). Cryo-EM structure of a native, fully glycosylated, cleaved HIV-1 envelope trimer. Science *351*, 1043–1048.

Lee, J.H., Andrabi, R., Su, C.Y., Yasmeen, A., Julien, J.P., Kong, L., Wu, N.C., McBride, R., Sok, D., Pauthner, M., et al. (2017). A broadly neutralizing antibody targets the dynamic HIV envelope trimer apex via a long, rigidified, and anionic β-hairpin structure. Immunity *46*, 690–702.

Li, Y., O'Dell, S., Walker, L.M., Wu, X., Guenaga, J., Feng, Y., Schmidt, S.D., McKee, K., Louder, M.K., Ledgerwood, J.E., et al. (2011). Mechanism of neutralization by the broadly neutralizing HIV-1 monoclonal antibody VRC01. J. Virol. *85*, 8954–8967.

Liao, H.X., Sutherland, L.L., Xia, S.M., Brock, M.E., Scearce, R.M., Vanleeuwen, S., Alam, S.M., McAdams, M., Weaver, E.A., Camacho, Z., et al. (2006). A group M consensus envelope glycoprotein induces antibodies that neutralize subsets of subtype B and C HIV-1 primary viruses. Virology *353*, 268–282.

Liu, J., Bartesaghi, A., Borgnia, M.J., Sapiro, G., and Subramaniam, S. (2008). Molecular architecture of native HIV-1 gp120 trimers. Nature *455*, 109–113.

Lyumkis, D., Julien, J.P., de Val, N., Cupo, A., Potter, C.S., Klasse, P.J., Burton, D.R., Sanders, R.W., Moore, J.P., Carragher, B., et al. (2013). Cryo-EM structure of a fully glycosylated soluble cleaved HIV-1 envelope trimer. Science *342*, 1484–1490.

McCoy, L.E., van Gils, M.J., Ozorowski, G., Messmer, T., Briney, B., Voss, J.E., Kulp, D.W., Macauley, M.S., Sok, D., Pauthner, M., et al. (2016). Holes in the glycan shield of the native HIV envelope are a target of trimer-elicited neutralizing antibodies. Cell Rep. *16*, 2327–2338.

McLellan, J.S., Pancera, M., Carrico, C., Gorman, J., Julien, J.P., Khayat, R., Louder, R., Pejchal, R., Sastry, M., Dai, K., et al. (2011). Structure of HIV-1

gp120 V1/V2 domain with broadly neutralizing antibody PG9. Nature *480*, 336–343.

Meyerson, J.R., Tran, E.E., Kuybeda, O., Chen, W., Dimitrov, D.S., Gorlani, A., Verrips, T., Lifson, J.D., and Subramaniam, S. (2013). Molecular structures of trimeric HIV-1 Env in complex with small antibody derivatives. Proc. Natl. Acad. Sci. USA *110*, 513–518.

Moldt, B., Rakasz, E.G., Schultz, N., Chan-Hui, P.Y., Swiderek, K., Weisgrau, K.L., Piaskowski, S.M., Bergman, Z., Watkins, D.I., Poignard, P., and Burton, D.R. (2012). Highly potent HIV-specific antibody neutralization in vitro translates into effective protection against mucosal SHIV challenge in vivo. Proc. Natl. Acad. Sci. USA *109*, 18921–18925.

Moody, M.A., Yates, N.L., Amos, J.D., Drinker, M.S., Eudailey, J.A., Gurley, T.C., Marshall, D.J., Whitesides, J.F., Chen, X., Foulger, A., et al. (2012). HIV-1 gp120 vaccine induces affinity maturation in both new and persistent antibody clonal lineages. J. Virol. *86*, 7496–7507.

Pancera, M., Shahzad-Ul-Hussan, S., Doria-Rose, N.A., McLellan, J.S., Bailer, R.T., Dai, K., Loesgen, S., Louder, M.K., Staupe, R.P., Yang, Y., et al. (2013). Structural basis for diverse N-glycan recognition by HIV-1-neutralizing V1-V2-directed antibody PG16. Nat. Struct. Mol. Biol. *20*, 804–813.

Pantophlet, R., Wrin, T., Cavacini, L.A., Robinson, J.E., and Burton, D.R. (2008). Neutralizing activity of antibodies to the V3 loop region of HIV-1 gp120 relative to their epitope fine specificity. Virology *381*, 251–260.

Pauthner, M., Havenar-Daughton, C., Sok, D., Nkolola, J.P., Bastidas, R., Boopathy, A.V., Carnathan, D.G., Chandrashekar, A., Cirelli, K.M., Cottrell, C.A., et al. (2017). Elicitation of robust tier 2 neutralizing antibody responses in nonhuman primates by HIV envelope trimer immunization using optimized approaches. Immunity *46*, 1073–1088.e6.

Pritchard, L.K., Spencer, D.I., Royle, L., Bonomelli, C., Seabright, G.E., Behrens, A.J., Kulp, D.W., Menis, S., Krumm, S.A., Dunlop, D.C., et al. (2015a). Glycan clustering stabilizes the mannose patch of HIV-1 and preserves vulnerability to broadly neutralizing antibodies. Nat. Commun. *6*, 7479.

Pritchard, L.K., Vasiljevic, S., Ozorowski, G., Seabright, G.E., Cupo, A., Ringe, R., Kim, H.J., Sanders, R.W., Doores, K.J., Burton, D.R., et al. (2015b). Structural constraints determine the glycosylation of HIV-1 envelope trimers. Cell Rep. *11*, 1604–1613.

Pugach, P., Ozorowski, G., Cupo, A., Ringe, R., Yasmeen, A., de Val, N., Derking, R., Kim, H.J., Korzun, J., Golabek, M., et al. (2015). A native-like SOSIP.664 trimer based on an HIV-1 subtype B env gene. J. Virol. *89*, 3380–3395.

Sanders, R.W., Vesanen, M., Schuelke, N., Master, A., Schiffner, L., Kalyanaraman, R., Paluch, M., Berkhout, B., Maddon, P.J., Olson, W.C., et al. (2002). Stabilization of the soluble, cleaved, trimeric form of the envelope glycoprotein complex of human immunodeficiency virus type 1. J. Virol. *76*, 8875–8889.

Sanders, R.W., Derking, R., Cupo, A., Julien, J.P., Yasmeen, A., de Val, N., Kim, H.J., Blattner, C., de la Peña, A.T., Korzun, J., et al. (2013). A next-generation cleaved, soluble HIV-1 Env trimer, BG505 SOSIP.664 gp140, expresses multiple epitopes for broadly neutralizing but not non-neutralizing antibodies. PLoS Pathog. *9*, e1003618.

Santos da Silva, E., Mulinge, M., and Perez Bercoff, D. (2013). The frantic play of the concealed HIV envelope cytoplasmic tail. Retrovirology *10*, 54.

Schiffner, T., de Val, N., Russell, R.A., de Taeye, S.W., de la Peña, A.T., Ozorowski, G., Kim, H.J., Nieusma, T., Brod, F., Cupo, A., et al. (2015). Chemical cross-linking stabilizes native-like HIV-1 envelope glycoprotein trimer antigens. J. Virol. *90*, 813–828.

Seaman, M.S., Janes, H., Hawkins, N., Grandpre, L.E., Devoy, C., Giri, A., Coffey, R.T., Harris, L., Wood, B., Daniels, M.G., et al. (2010). Tiered categorization of a diverse panel of HIV-1 Env pseudoviruses for assessment of neutralizing antibodies. J. Virol. *84*, 1439–1452.

Sharma, S.K., de Val, N., Bale, S., Guenaga, J., Tran, K., Feng, Y., Dubrovskaya, V., Ward, A.B., and Wyatt, R.T. (2015). Cleavage-independent HIV-1 Env trimers engineered as soluble native spike mimetics for vaccine design. Cell Rep. *11*, 539–550.

Sok, D., Pauthner, M., Briney, B., Lee, J.H., Saye-Francisco, K.L., Hsueh, J., Ramos, A., Le, K.M., Jones, M., Jardine, J.G., et al. (2016). A prominent site of antibody vulnerability on HIV envelope incorporates a motif associated with CCR5 binding and its camouflaging glycans. Immunity *45*, 31–45.

Steckbeck, J.D., Sun, C., Sturgeon, T.J., and Montelaro, R.C. (2013). Detailed topology mapping reveals substantial exposure of the "cytoplasmic" C-terminal tail (CTT) sequences in HIV-1 Env proteins at the cell surface. PLoS One *8*, e65220.

Steichen, J.M., Kulp, D.W., Tokatlian, T., Escolano, A., Dosenovic, P., Stanfield, R.L., McCoy, L.E., Ozorowski, G., Hu, X., Kalyuzhniy, O., et al. (2016). HIV vaccine design to target germline precursors of glycan-dependent broadly neutralizing antibodies. Immunity *45*, 483–496.

Stewart-Jones, G.B., Soto, C., Lemmin, T., Chuang, G.Y., Druz, A., Kong, R., Thomas, P.V., Wagh, K., Zhou, T., Behrens, A.J., et al. (2016). Trimeric HIV-1-Env structures define glycan shields from clades A, B, and G. Cell *165*, 813–826.

Stieh, D.J., King, D.F., Klein, K., Aldon, Y., McKay, P.F., and Shattock, R.J. (2015). Discrete partitioning of HIV-1 Env forms revealed by viral capture. Retrovirology *12*, 81.

Torrents de la Peña, A., Julien, J.P., de Taeye, S.W., Garces, F., Guttman, M., Ozorowski, G., Pritchard, L.K., Behrens, A.J., Go, E.P., Burger, J.A., et al. (2017). Improving the immunogenicity of native-like HIV-1 envelope trimers by hyperstabilization. Cell Rep. *20*, 1805–1817.

Veillette, M., Coutu, M., Richard, J., Batraville, L.A., Désormeaux, A., Roger, M., and Finzi, A. (2014). Conformational evaluation of HIV-1 trimeric envelope glycoproteins using a cell-based ELISA assay. J. Vis. Exp. *91*, 51995.

Walker, L.M., Huber, M., Doores, K.J., Falkowska, E., Pejchal, R., Julien, J.P., Wang, S.K., Ramos, A., Chan-Hui, P.Y., Moyle, M., et al.; Protocol G Principal Investigators (2011). Broad neutralization coverage of HIV by multiple highly potent antibodies. Nature *477*, 466–470.

Zhou, T., Georgiev, I., Wu, X., Yang, Z.Y., Dai, K., Finzi, A., Kwon, Y.D., Scheid, J.F., Shi, W., Xu, L., et al. (2010). Structural basis for broad and potent neutralization of HIV-1 by antibody VRC01. Science *329*, 811–817.

Zhou, T., Lynch, R.M., Chen, L., Acharya, P., Wu, X., Doria-Rose, N.A., Joyce, M.G., Lingwood, D., Soto, C., Bailer, R.T., et al.; NISC Comparative Sequencing Program (2015). Structural repertoire of HIV-1-neutralizing antibodies targeting the CD4 supersite in 14 donors. Cell *161*, 1280–1292.

Zhu, P., Liu, J., Bess, J., Jr., Chertova, E., Lifson, J.D., Grisé, H., Ofek, G.A., Taylor, K.A., and Roux, K.H. (2006). Distribution and three-dimensional structure of AIDS virus envelope spikes. Nature *441*, 847–852.

STAR★METHODS

KEY RESOURCES TABLE

REAGENT or RESOURCE	SOURCE	IDENTIFIER
Antibodies		
2G12, PG9, PG16, b12, 447-52D, 5F3, 4E10, 2F5, F240, 4B3	Polymun Scientific	N/A
48d, E51, 10-1074, 2191, 2219, 3074, 3869, 35O22, 98-6D, 167-D	NIH AIDS Reagent Program	N/A
39F, 19b, 3BC176, 3BC315, b6, F105, PGT121, PGT135, PGT145, PGT151, VRC01	Produced in house	N/A
17b, 7B2	James Robinson	N/A
Mouse anti-human c-Myc IgG1 (9E10)	Produced in house	N/A
Goat anti-rabbit IgG Fc	Jackson ImmunoResearch	Cat# 111-005-046; RRID: AB_2337917
Rabbit IgG	Bio-Rad	Cat# PRABP01; RRID: AB_321631
Mouse anti-rabbit IgG biotinylated (Clone RG-96)	Sigma	Cat# B5283; RRID: AB_258574
Goat anti-guinea pig IgG F(ab')2	Jackson ImmunoResearch	Cat# 106-005-006; RRID: AB_2337395
Guinea pig IgG	Jackson ImmunoResearch	Cat# 006-000-003; RRID: AB_2337022
Donkey anti-guinea pig IgG (H+L) biotinylated	Sigma	Cat# 3700388
F(ab')2-goat anti-human IgG Fc PE	Invitrogen	Cat# H10104; RRID: AB_1500728
Goat anti-human IgG Fc biotinylated	Southern Biotech	Cat# 2048-08
Bacterial and Virus Strains		
Pseudotyped HIV-1: MW965.26, ConM, ConS, MN, SF162, TV1.29	Produced in house	N/A
Chemicals, Peptides, and Recombinant Proteins		
Soluble CD4 D1D2 His-tagged	Produced in house	N/A
ConSOSL.UFO.664 gp140	This paper	N/A
ConSOSL.UFO.664 Myc-His tagged	This paper	N/A
ConSOSL.UFO V3 cys-cyclised peptide	Synthesized by JPT	N/A
MN.3 V3 scrambled linear peptide	Synthesized by JPT	N/A
Polyethyleneimine "MAX" (MW 40,000)	Polysciences	Cat# 24765
Poly-HRP 40	Fitzgerald	Cat# 65RMS104PHRP
Casein buffer	ThermoFisher Scientific	Cat# 37528
Collagen I, rat tail	Invitrogen	Cat# A1048301
Recombinant human bFGF	GIBCO	Cat# 13256029
Luminata Crescendo ELISA HRP substrate	Merck Millipore	ELLUR0200
Experimental Models: Cell Lines		
HEK293T/17 cells	ATCC	Cat# CRL-11268; RRID: CVCL_1926
MYC 1-9E10.2 [9E10] hybridoma	ATCC	Cat# CRL-1729; RRID: CVCL_G671
Human skeletal muscle primary cells	DV Biologics	Cat# AM003-F-DMD
TZM-bl cells	NIH AIDS Reagent Program	Cat# 8129; RRID: CVCL_B478
Experimental Models: Organisms/Strains		
Female New Zealand white rabbits	Charles River	Crl:KBL(NZW) strain code 052
Female Dunkin-Hartley guinea pigs	Charles River	Crl:HA strain code 051
Female BALB/c mice	Charles River	Balb/cAnNCrl strain code 028
Recombinant DNA		
pSG3ΔEnv	NIH AIDS Reagent Program	Cat# 11051

(*Continued on next page*)

Continued

REAGENT or RESOURCE	SOURCE	IDENTIFIER
Software and Algorithms		
Flowjo v10	Treestar	https://www.flowjo.com/
Prism v7	GraphPad Software	https://www.graphpad.com/scientific-software/prism/
Chromlab v4	BioRad	https://www.bio-rad.com/en-uk/product/chromlab-software?ID=MFCVPXIVK
Microsoft Office	Microsoft	https://www.office.com/
Driftscope v2.8	Waters	http://www.waters.info/waters/library.htm?lid=10103987&cid=511436
MassLynx v4.1	Waters	http://www.waters.com/waters/en_GB/MassLynx-Mass-Spectrometry-Software-/nav.htm?cid=513164&locale=en_GB
Byonic v2.7	Protein Metrics	https://www.proteinmetrics.com/products/byonic/
Byologic v2.3	Protein Metrics	https://www.proteinmetrics.com/products/byologic/

CONTACT FOR REAGENT AND RESOURCE SHARING

Further information and requests for resources and reagents should be directed to and will be fulfilled by the Lead Contact, Robin J. Shattock (r.shattock@imperial.ac.uk).

EXPERIMENTAL MODEL AND SUBJECT DETAILS

Cells

HEK293T.17 cells (ATCC) and TZM-bl cells (NIH AIDS Research and Reference Reagent Program) were maintained in complete DMEM (Sigma) supplemented with 10% Fetal Bovine Serum (FBS), 2 mM glutamine, 100 U/mL penicillin G, and 100 μg/mL streptomycin (GIBCO). Duchene Muscular Dystrophy (DMD) human skeletal muscle primary cells were cultured as recommended by the supplier using rat collagen to coat flasks and culture plates and recombinant human bFGF to supplement the Muscle Cellulation medium (M-GRO medium, DV Biologics). Cells were handled in a sterile cabinet and cultured in a humidified, 5% CO2, 37°C incubator.

Animals

Female New Zealand white rabbits aged 2.5 months old were placed into 3 groups of n = 6. Female guinea pig (Dunkin-Hartley) aged 10 weeks old were placed into 2 groups of n = 6. Female BALB/c mice aged 6-8 weeks were placed into 2 groups of n = 5. Animals were handled and procedures were performed in accordance with the terms of a project license granted under the UK Home Office Animals (Scientific Procedures) Act 1986.

METHOD DETAILS

Env and sCD4 proteins production

HIV-1 *Env* and *sCD4 D1D2 His-Tagged* genes (codon optimized for *Homo sapiens* expression) were either created using published sequences or designed *in silico*, and cloned into pcDNA3.1(+) using GeneArt gene synthesis service (ThermoFisher Scientific). pConS gp160 clone was donated by David Montefiori. sCD4 D1D2 His-Tag was produced by transfecting 293T.17 cells using polyethyleneimine (PEI) (Polysciences) and purified on a cOmplete® His-Tag purification column (Roche), following the manufacturer's instructions, and stored in PBS at −20°C.

Env soluble trimers ConSOSL.UFO.664 and ConSOSL.UFO.664 Myc-HIS tagged version were produced in 293T.17 using triple layered T-175 flasks. After transfection, FreeStyle 293 medium (GIBCO) was added (90 mL per flask) and harvested 48h later. Cellular debris were pelleted and the supernatant filtered (0.45 μm). The trimers were concentrated using 100kDa MWCO Amicon ultrafiltration columns (Merck Millipore) and transferred in PBS. Trimers were then purified by size exclusion chromatography (SEC) on an NGC medium pressure liquid chromatography (MPLC) system (BioRad) using an Enrich SEC 650 column (BioRad). The collected trimer fractions were then concentrated using ultrafiltration columns, aliquoted and stored at −80°C for further analysis.

HIV-1 monoclonal Abs and 9E10

mAbs were obtained from their producers, purchased from commercial suppliers or produced in house. 2G12, PG9, PG16, b12, 447-52D, 5F3, 4E10, 2F5, F240 and 4B3 were acquired from Polymun Scientific (Austria); 17b and 7B2 were donated by James Robinson; 48d, E51, 10-1074, 2191, 2219, 3074, 3869, 35O22, 98-6D and 167-D were obtained from the NIH AIDS Research and Reference Reagent Program; expression vectors for 39F, 19b, 3BC176, 3BC315, PGT121, PGT135, PGT145, F105 and b6 were obtained from the IAVI Neutralizing Ab Consortium and produced in house; expression vectors for VRC01 and PGT151 were generated in house. In house mAbs were produced in 293T.17 cells and purified on HiTrap protein A HP column (GE LifeSciences). 9E10 Ab was produced from a hybridoma culture and purified on protein A column.

Cell surface-binding assays

FC

Transient expression of Env was assessed by FC. 48 h post transfection, 293T.17 cells were rinsed with PBS, dissociated with cell dissociation buffer (GIBCO), washed with FACS buffer (2.5% FBS, 1 mM EDTA, 25 mM HEPES in 1X PBS) and pelleted at 600 × g, 5 min. Cells were resuspended in FACS buffer and counted using trypan blue. Cells were then stained with aqua viability dye (1:400) for 20 min at RT in the dark, then washed twice with FACS buffer. 10 μg/mL in 100 μL FACS buffer of primary human IgG anti-Env Ab were used to stain 1 × 10ˆ6 cells per well in U bottom 96-well plates, 30 min at RT in the dark. For the titrations, 10-fold dilution series of the primary Ab were used. When needed, sCD4 was added to the primary Ab mix at a final concentration of 20 μg/mL. Cells were then washed twice with 125 μL FACS buffer and secondary F(ab')2-goat anti-human IgG Fc PE Ab (Invitrogen) was added onto the cells at 0.1 μg/10ˆ6 cells in 100 μL FACS buffer per well. After 20 min incubation in the dark, cells were washed twice, resuspended in 100 μL PBS and fixed with an additional 100 μL 3% paraformaldehyde (Polysciences), final 1.5%. Samples were acquired on a LSRFortessa FC (BD) using FACSDiva (BD) and data interpreted using FlowJo v.10.1 software (Treestar). Mean fluorescence intensity (MFI) values of the 'live cells' gate were used to analyze the results. Results are either reported as MFI and traces (all live cells) or reported as mAb:2G12 ratio in order to normalize the data – 2G12 giving among the highest binding signal to the tested designs. A pcDNA3 empty vector control was included to allow subtraction of each mAb background.

Cell-based ELISA

24 hr before transfection, cells were seeded onto rat collagen (Invitrogen) pre-coated 96-well white Costar® plates (Corning) in complete DMEM for 293T.17 and in supplemented M-GRO for DMD cells at 35 × 10ˆ4 and 30x10ˆ4 cells/well respectively. Transfections were carried out using a 1:3 DNA to PEI (w:w) ratio with 150 ng DNA + 450 ng PEI per well (triplicate wells for 293T.17, duplicate wells for DMD cells), in DMEM without antibiotics nor FBS at 37°C. 6 hr later, 100 μL/well of culture medium was added. 42 hr after, plates are emptied and washed once with 200 μL/well Tris Buffer Saline (TBS, 20 mM Tris). Plates were blocked with 100 μL/well casein buffer (CB) (Thermo Scientific) at RT, 40 min. CB was removed and primary human IgG anti-Env mAbs were added onto the cells at 1 μg/mL in 100 μL CB per well. After 1 hr incubation at RT, plates were washed 3x with TBS and detection Ab added: Goat anti-human IgG Fc biotinylated Ab (Southern Biotech) 1:10,000 dilution in CB (100 μL/well), 1 hr at RT. Followed 3x TBS washes and the addition of poly-HRP40 (Fitzgerald) 1:10,000 dilution in CB, 40 min in the dark at RT. Plates were developed using 80 μL/well of Luminata® Crescendo ELISA substrate (Merck Millipore) and luminescence was measured as relative lights units by a LUMIstar Omega microplate reader (BMG Labtech). A pcDNA3 empty vector control was included to each plate in order to subtract respective mAb background and data were then analyzed in Microsoft Excel and GraphPad Prism and expressed as mAb:2G12 ratios.

We evaluated whether the transfection conditions in T-75 flask (FC conditions) were comparable to transfection of cells directly onto the ELISA plate. Transfected cells from T-75 flasks were trypsinized 48 hr after transfection and seeded onto an ELISA plate, and the assay performed 24 hr after, and compared to transfected cells directly seeded onto the plate (Figure S3A-B). An IgG standard was included to these optimization assays. A comparison of collagen coated versus non-coated plates was also carried out (Figure S3C). This proved to be an improvement and allowed higher throughput with less cell loss during washing steps and showed similar Ab binding patterns to non-coated plates.

Blue Native Western Blotting

Samples were prepared using NuPAGE Novex sample loading buffer and loaded onto a 4%–12% polyacrylamide Bis-Tris or 3%–8% Tris-Acetate NuPAGE (Invitrogen) followed by transfer into nitrocellulose membranes (Invitrogen) or blue staining. Membranes were blocked using 2% (w/v) Bovine Serum Albumin (BSA) (Sigma), 0.05% Tween20 (v/v) in PBS. Primary mAb human anti-Env IgG (1 μg/mL) was then added. The membranes were washed and secondary goat anti-human IgG Fc biotinylated Ab added (1:10,000). After a washing step, the membranes were incubated with streptavidin-HRP 1:500 (R&D Systems), then washed, dried, WB Luminata® Classico (Merck Millipore) applied and finally developed on Amersham Hyperfilm ECL (GE LifeSciences). Native gels were stained using PageBlue™ protein staining solution (ThermoFisher Scientific) following the manufacturer's protocol.

Glycan analysis by HILIC-UPLC

N-linked glycans were released from envelope glycoproteins with Peptide-N-Glycosidase F (PNGase F), fluorescently labelled with 2-aminobenzoic acid and analyzed by HILIC-UPLC, as previously described (Behrens et al., 2016; Pritchard et al., 2015b) and Endo H released glycans enabled the quantitation of oligomannose-type glycans (Pritchard et al., 2015b).

Assigning glycan compositions using tandem ion mobility ESI MS

The compositions of the glycans were determined by analyzing released glycans from trimers by PNGase F digestion using ion mobility MS (Behrens et al., 2016). Negative ion mass, collision-induced dissociation (CID) and ion mobility spectra were recorded with a Waters Synapt G2Si mass spectrometer (Waters Corp.) fitted with a nano-electrospray ion source. Waters Driftscope (version 2.8) software and MassLynx^{TM} (version 4.1) was used for data acquisition and processing. Spectra were interpreted as described previously (Harvey, 2005a, b, c; Harvey et al., 2008). The results obtained served as the basis for the creation of sample-specific glycan libraries, which were used for subsequent site-specific N-glycosylation analyses.

Site-specific N-glycosylation analysis

Before proteolytic digestion, trimers were denatured and alkylated by incubation for 1 hr at room temperature (RT) in a 50 mM Tris/HCl, pH 8.0 buffer containing 6 M urea and 5 mM dithiothreitol (DTT), followed by the addition of 20 mM iodacetamide (IAA) for a further 1 hr at RT in the dark, and then additional DTT (20 mM) for another 1h, to eliminate any residual IAA. The alkylated trimers were buffer-exchanged into 50 mM Tris/HCl, pH 8.0 using Vivaspin columns and digested with trypsin and elastase (Mass Spectrometry Grade, Promega) at a ratio of 1:30 (w/w). Glycopeptides were selected from the protease-digested samples using the ProteoExtract Glycopeptide Enrichment Kit (Merck Millipore). Enriched glycopeptides were analyzed by LC-ESI MS on an Orbitrap fusion mass spectrometer (ThermoFisher Scientific), as previously described (Behrens et al., 2016), using higher energy collisional dissociation (HCD) fragmentation. Data analysis and glycopeptide identification were performed using Byonic^{TM} (Version 2.7) and Byologic^{TM} software (Version 2.3; Protein Metrics Inc.), as previously described (Behrens et al., 2016).

Negative-stain electron microscopy and NanoDSF

Purified ConSOSL.UFO.664 trimers, either alone or after 2 hr incubation with 6X molar excess (Fab:trimer) of VRC01 Fab, were diluted to ~0.02 mg/mL and adsorbed onto glow-discharged carbon-coated Cu400 mesh grids. The grids were stained with 2% (w/v) uranyl formate for 60 s. Data collection and processing methods have been reported previously (de Taeye et al., 2015).

Thermal stability measurements were obtained by loading ~0.25 mg/mL of ConSOSL.UFO.664, B41 SOSIP.664, or BG505 SOSIP.664 into a glass capillary tube and analyzed using a Prometheus NT.48 NanoDSF instrument (NanoTemper Technologies).

Animals and immunization

Female New Zealand white rabbits aged 2.5 months old were placed into 3 groups of n = 6. Two groups of rabbits were injected intramuscularly (IM; quadriceps) 3 times at 4-week intervals with 100 μg of plasmid DNA (pConSOSL.UFO.664 or pConSOSL. UFO.750) followed by electroporation (EP) using 5-mm electrodes using an ECM 830 square-wave electroporation system (BTX) (3 pulses of 100 V each. followed by 3 pulses of the opposite polarity with each pulse (P_{ON}) lasting 50 ms and an interpulse (P_{OFF}) interval of 50 ms) and boosted with 25μg of protein plus AddaVax adjuvant at week 20 and 1 group was immunized 3 times at week 4, 8 and 20 with 25μg of protein plus AddaVax adjuvant.

Guinea pig (Dunkin-Hartley) aged 10 weeks old were placed into 2 groups of n = 6. Guinea pigs were injected IM 4 times at 3-week interval with 20 μg of plasmid DNA (pConSOSL.UFO.664 or pConSOSL.UFO.750) in 50 μL PBS followed by EP. 2 animals died, 1 in each group) under anesthesia on the day of the 3rd immunization. Post-mortem analysis revealed a lung infection which led to respiratory deficiency under anesthesia.

Groups of BALB/c mice were injected IM 4 times at 3-week interval with 20 μg of plasmid DNA (pConSOSL.UFO.664 or pConSOSL.UFO.750) in 50 μL PBS followed by EP.

For all animals, serum samples were collected at each immunization time point and at additional time points indicated in the figures.

Antigen specific IgG capture ELISA

For rabbits and guinea pigs: MaxiSorp high binding ELISA plates were coated with 100 μL/well of 5 μg/mL in PBS of 9E10 mouse anti-cMyc Ab. Capture Ab for the standard IgG were coated using PBS as diluent with 1:3000 Goat anti-Rabbit IgG Fc for Rabbit and 1:2,000 dilution of Goat anti-Guinea pig IgG F(ab')2 (100 μL/well) (Jackson Immuno Research) for guinea pigs. The plates were incubated overnight at 4°C. The plates were washed 4 times with PBS-Tween20 0.05% and then 200 μL/well of Casein Buffer (CB) was added to block the plates. After 1 hr at 37°C, plates were washed 4 times with PBS-Tween20. 100 μL/well of 1 μg/mL (in CB) tagged ConSOSL.UFO.664 Myc-HIS SEC purified trimers were added onto the 9E10 coated wells whereas CB only was added to the standard wells. After a washing step, 50 μL of diluted serum samples: 1:100, 1:1,000 and 1:10,000 in CB were added in triplicate onto the plates as well as the Rabbit (Bio-Rad) or Guinea pig IgG standard Ab (Jackson Immuno Research) in a 1:5 serial dilution starting at 200 ng/mL. Plates were incubated for 1 hr at 37°C, washed 4 times and then the Mouse anti-Rabbit IgG biotinylated (Sigma) or the Donkey Anti-Guinea pig IgG (H+L) biotinylated Ab (Sigma) diluted 1:25,000 in CB (100 μL/well) was added. Plates were incubated for 1 hr at 37°C and washed. 1:10,000 dilution of poly-HRP40 (Fitzgerald) in CB was added (100 μL/well) – 1 hr at 37°C then wash. Plates were developed with 50 μL/well TMB (KPL) and the reaction stopped after 5 min using 50 μL/well Stop solution (Insight Biotechnologies, UK). The absorbance was read on a KC4 Spectrophotometer at 450 nm (BioTek). For the V3 competitive ELISA, samples were incubated 30 min prior loading onto the ELISA plates with 10 μg/mL ConSOSL.UFO V3 cys-cyclised peptide (CTRPNNNTRKSIR IGPGQWFYATGDIIGDIRQAHC) or MN.3 V3 scrambled linear peptide (HTGKYTYPTNIAIRGRGNKFRNKKI) as a control and the specific binding to ConSOSL.UFO.664 Myc-HIS captured antigen measured. For human mAbs titration, 2.5 μg/mL 9E10 was coated

following the same protocol. 1:5 dilution series of mAbs starting at 10 μg/mL were loaded. Secondary Mouse anti-Human IgG Fc biotynilated was used (Sigma) and development stopped at 8 min. For mouse ELISAs, ConSOSL.UFO.664 untagged protein was coated directly onto the plate (1 μg/mL, 100 μL/well in PBS) and 1:1,000 dilution of the captured goat anti-Kappa and anti-Lambda was used to coat the standard wells (Southern Biotech). After an overnight incubation at 4°C, plates were washed, blocked with 1% BSA + 0.05% Tween20 (Sigma) in PBS (200 μL/well) and incubated for 1 hr at 37°C. The plates were then washed, incubated with samples at describe above and the standard IgG, IgG1 and IgG2a added to the standard wells (start at 1 μg/mL then 1:5 dilution series). Following a 1 hr incubation at 37°C, plates were washed, incubated with 1:2,000 secondary goat anti-IgG-HRP, IgG1-HRP or IgG2a-HRP (Southern Biotech). Plates were then developed as described above.

Neutralization assays

HIV-1 pseudoviruses were produced in HEK293T cells (ATCC) using a combination of pSG3ΔEnv (NIH AIDS Research and Reference Reagent Program) and Envelope plasmids (MW965.26, ConM, ConS, MN, SF162, TV1.29) to transfect the cells using Lipofectamine 2000 (Invitrogen). 48 hr after transfection, supernatants containing pseudoviruses were clarified, filtered, aliquoted and stored at −80°C. The TZM-bl reporter cell line (NIH AIDS Research and Reference Reagent Program) were seeded in 96 well plates in complete medium one day before infection. Serially diluted rabbit sera were either directly incubated with pseudoviruses for 1 hr at room temperature or pre-incubated with 20 μg/mL ConSOSL.UFO V3 cys-cyclised peptide or MN.3 V3 scrambled linear peptide for 1 hr at room temperature (sera from week 22) and then pseudoviruses added for an additional hour. The sera mixture were then added onto the cells and incubated at 37°C, 5% $CO2$ for 48 hr. Following this incubation, medium was removed and cells were washed once with PBS then lysed in reporter lysis buffer (Promega). Using the luciferase assay kit (Promega) the luciferase activity was measure on a LUMIstar Omega microplate reader (BMG Labtech). Each condition tested was performed in duplicate except for the V3 competition neutralization assays were some rabbit sera from week 22 were tested in duplicate or 1 well only due to the limited quantities of peptide. Uninfected cells were used to determine the luciferase activity background. Analysis of nonlinear regression curves allow the determination of the ID50 (50% inhibition of infectivity) which are reported in the figures.

QUANTIFICATION AND STATISTICAL ANALYSIS

Statistical analyses were carried out using a One-way ANOVA with Sidak’s multiple comparisons or unpaired t test in order to determine statistical significance using GraphPad Prism v7.0h.

Cell Reports, Volume *24*

Supplemental Information

Rational Design of DNA-Expressed

Stabilized Native-Like HIV-1 Envelope Trimers

Yoann Aldon, Paul F. McKay, Joel Allen, Gabriel Ozorowski, Réka Felfödiné Lévai, Monica Tolazzi, Paul Rogers, Linling He, Natalia de Val, Katalin Fábián, Gabriella Scarlatti, Jiang Zhu, Andrew B. Ward, Max Crispin, and Robin J. Shattock

SUPPLEMENTAL FIGURES

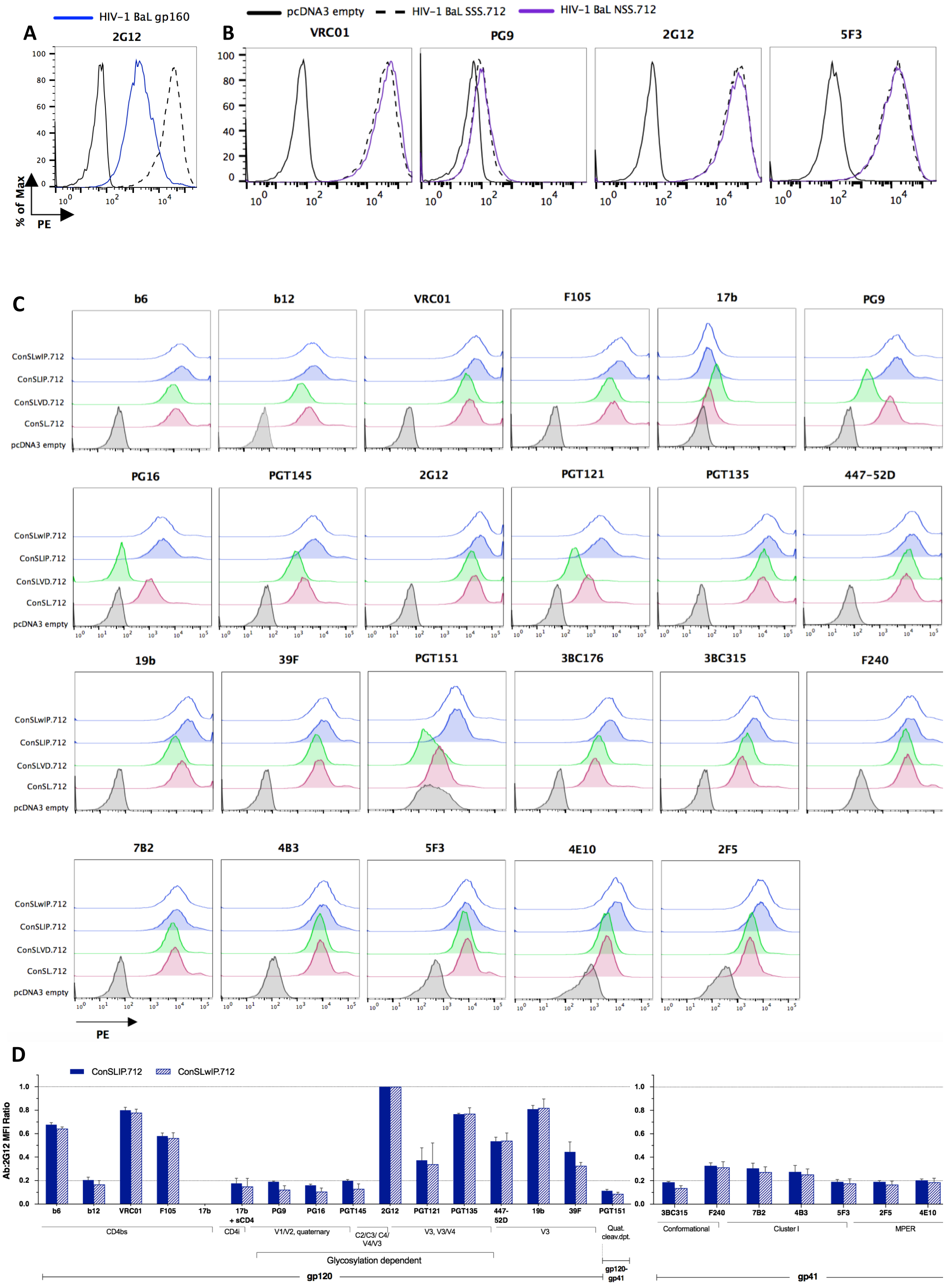

Figure S1. Effects of truncation at position 712 and signal sequence on surface expression of BaL and impact of IP, VD and linker orientation on the cleavage independent ConSL.712 design. Related to Figures 2 and 3. (**A**) FC analysis of HIV-1 BaL Env comparing the expression levels of gp160 and truncated version BaL SSS.712 transiently expressed in 293T.17 cells. Representative of n ≥ 2 independent experiments.

(**B**) Comparison of native signal sequence (NSS) versus secretion signal sequence (SSS) and their impact on Env surface antigenicity in 293T.17 cells. 293T.17 cells transfected with a pcDNA3 empty vector were included as a negative control to determine each mAb background. Representative of n ≥ 2 independent experiments. (**C**) 293T.17 cells were transiently transfected with the indicated constructs and traces were plotted in FlowJo. Representative of n ≥ 2 independent experiments. (**D**) Ab binding profile of ConSLIP.712 and ConSLwIP.712 were analysed as in Figure 2. Error bars represent mean ± SEM with n ≥ 2 independent experiments.

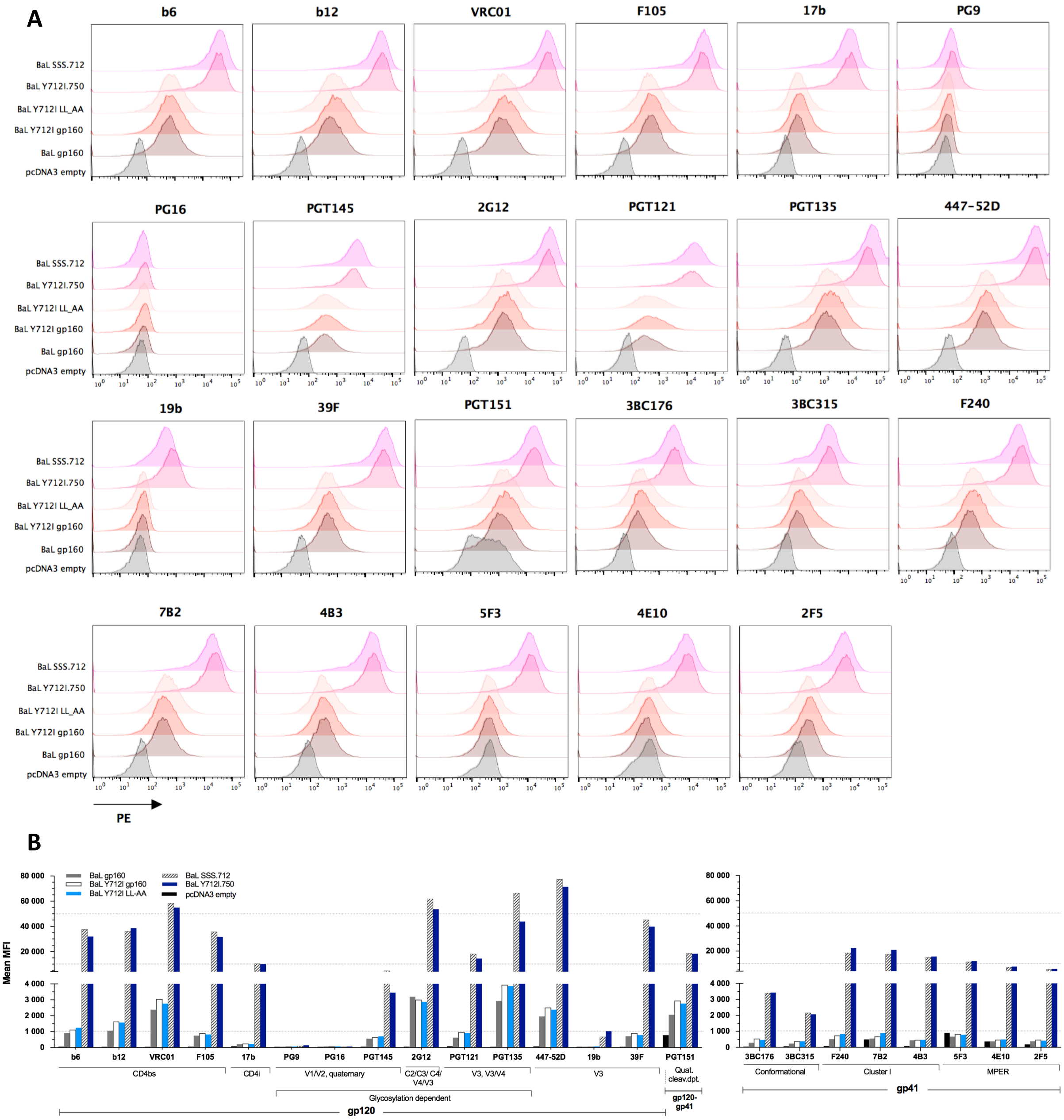

Figure S2. Effect of the endocytosis/recycling motif mutations and CT truncations on BaL Env surface expression. Related to Figure 3. (**A**) Traces overlay of extended BaL constructs FC data from Figure 3. (**B**) Extended mAbs panel from Figure 3.B with mean MFI values plotted. Representative of n ≥ 2 independent experiments.

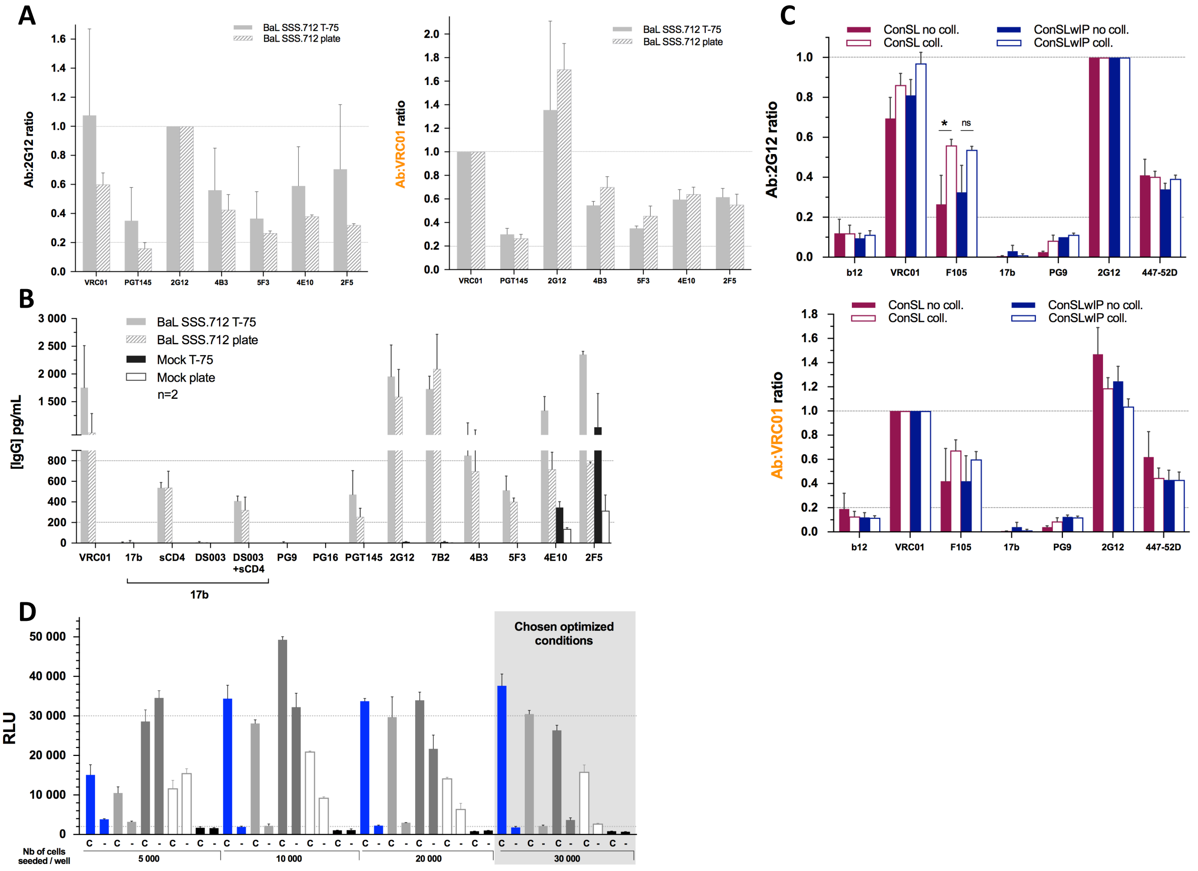

Figure S3. Cell ELISA assay optimization and validation for 293T.17 and human skeletal primary muscle cells. Related to Figure 3. (**A**) Comparison of CE transfection and seeded conditions using BaL SSS.712 expressing 293T.17 cells either transfected in T-75 then seeded onto the ELISA plate or cells directly seeded and transfected onto the plate. Both mAb:2G12 and mAb:VRC01 are shown (n ≥ 2). (**B**) Same as (**A**) using an extended panel and a standard IgG. DS003 is a microbicide from the International Partnership Microbicide and binds the CD4bs. (**C**) Comparison of collagen coated (coll.) plates versus non coated (no coll.) plates. Both mAb:2G12 and mAb:VRC01 are shown (n ≥ 2). (**D**) Muscle cells CE optimization using 4 mAbs at 10 µg/mL (2G12 blue, VRC01 light grey, PGT135 dark grey, F240 white) and different cell concentration seeded per well. ConSLIP.712 (‘C’ on the x axis) was used for optimization along with pcDNA3 empty (‘-‘ on the x axis) vector transfected wells to determine mAbs background. Best conditions are highlighted in grey. Error bars represent mean ± SEM with n ≥ 2 independent experiments for (**A**), (**B**) and (**C**) and mean ± SD of duplicate wells for (**D**). Unpaired t test *p* values: *<0.05.

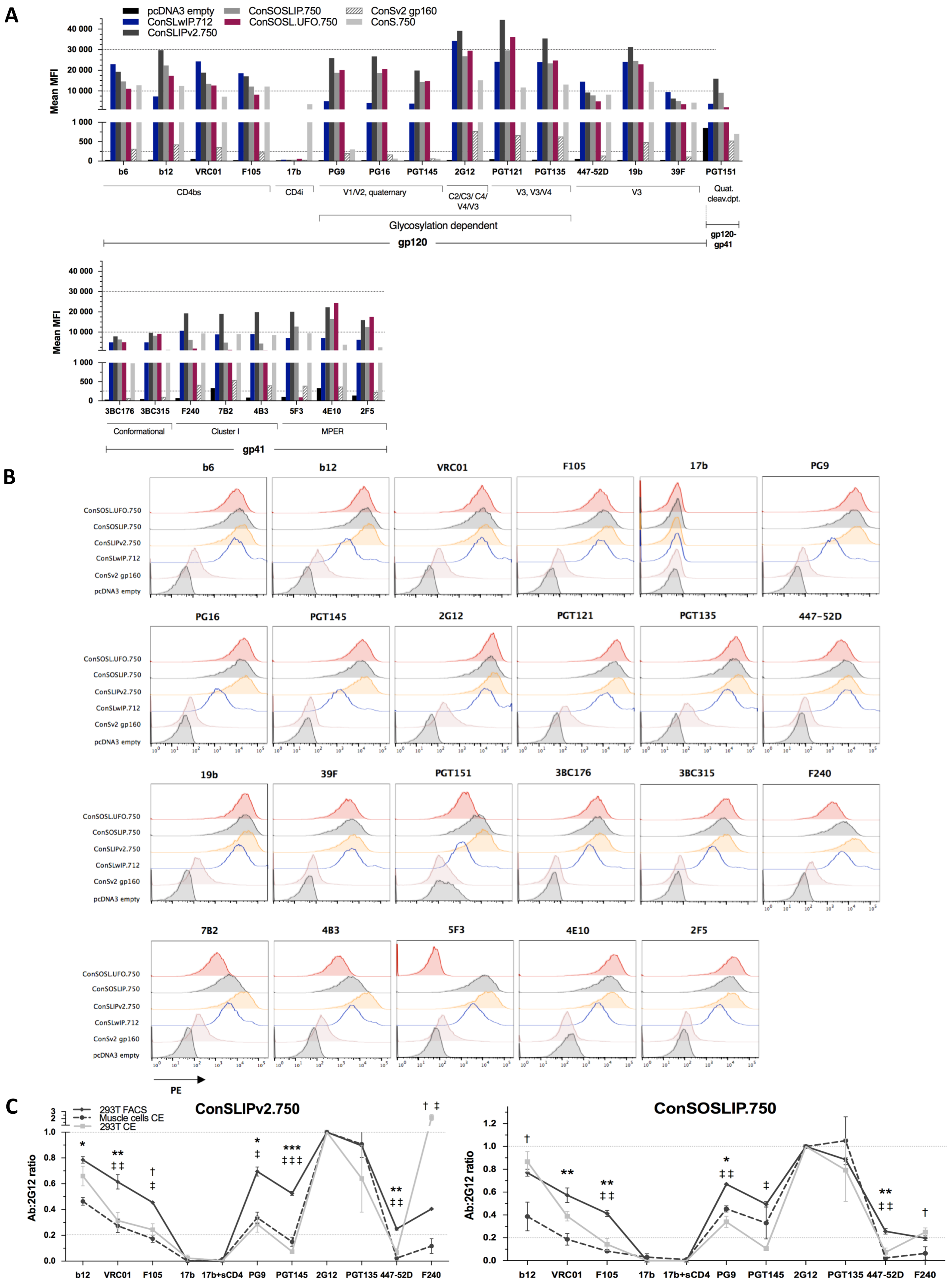

Figure S4. Enhancement of bNAbs binding and subsequent reduction of nNAbs binding by gp41 HR1 stabilization - Flow cytometry and cell ELISA. Related to Figure 4. (**A**) Mean MFI values corresponding to the bar graph of Figure 4.A of Env constructs expressed in 293T.17 and analysed by FC for 23 mAb. Representative of n ≥ 2 independent experiments (for ConSOSL.UFO.750 n ≥ 3). (**B**) Traces overlay of the corresponding experiment expressed in 293T.17 cells. Representative of n ≥ 2 independent experiments (ConSOSL.UFO.750 n ≥ 3). (**C**) Comparison of ConSLIPv2.750 and ConSOSLIP.750 epitope profiles in

muscle cell CE, 293T.17 CE and FC ($n \geq 2$). One-way ANOVA with Sidak's multiple comparisons, *p* values: *<0.05, **<0.01, ***<0.001 where each sign in (**C**) compares: *Muscle Cells CE vs. 293T.17 FC; † Muscle Cells CE vs. 293T.17 CE; ‡ 293T.17 FC vs. 293T CE.

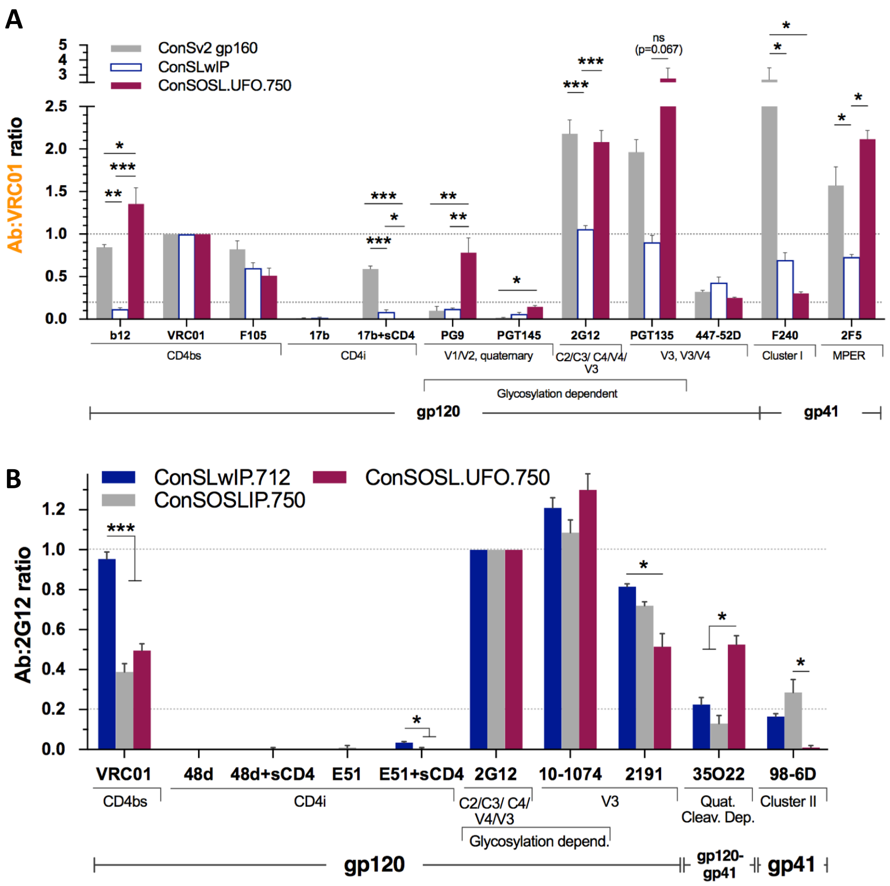

Figure S5. Enhancement of bNAbs binding and subsequent reduction of nNAbs binding by gp41 HR1 stabilization – Cell ELISA. Related to Figure 4. (**A**) 293T.17 CE assessing ConSv2 gp160, ConSLwIP.712 and ConSOSL.UFO.750 using mAb:VRC01 ratio to plot the mean ± SEM values ($n \geq 3$, except ConSv2 gp160 PGT135 $n = 2$). (**B**) Comparison of ConSLwIP.712, ConSOSLIP.750 and ConSOSL.UFO.750 in 293T.17 CE for mAb: VRC01, 48d (+/- sCD4), E51 (+/- sCD4), 2G12, 10-1074, 2191, 35O22 and 98-6D ($n \geq 2$). One-way ANOVA with Sidak's multiple comparisons, p values: *<0.05, **<0.01, ***<0.001.

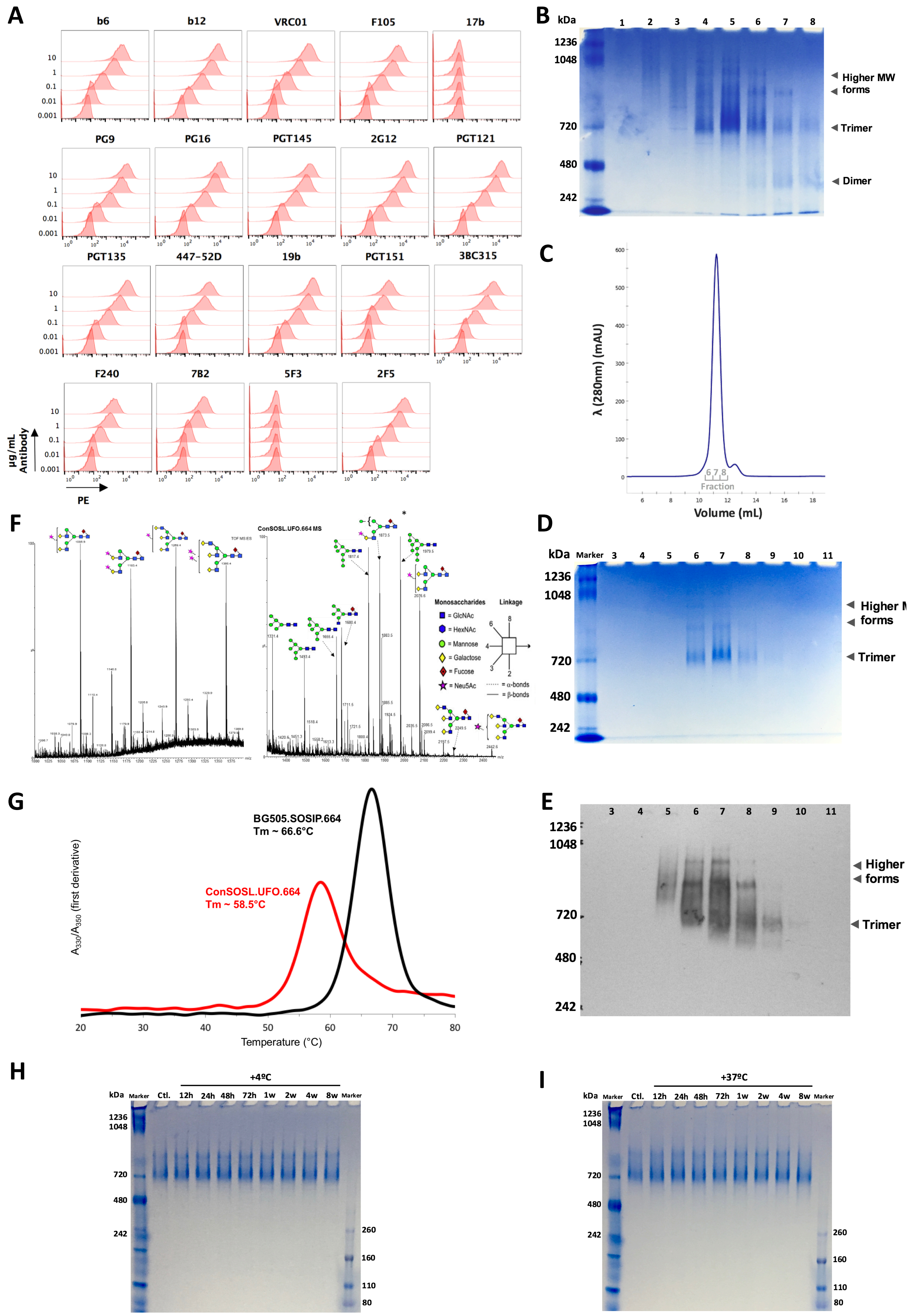

Figure S6. Antibodies titration against ConSOSL.UFO.750 and structural and glycosylation analysis of ConSOSL.UFO.664 Env trimers. Related to Figures 5 and 6. (**A**) Traces corresponding to the mean MFI value plotted in Figure 5. Representative of n = 2 independent experiments. (**B**) Colloidal blue stain of a native

gel running fractions of the SEC purification from Figure 6.A. (**C**) Fractions 4-6 of Figure 6.A were run a second time through a SEC column and the obtained SEC profile is shown. (**D**) Colloidal blue stain of a native gel running fractions of the SEC purification from (**C**). (**E**) Native PAGE western blot of (**D**) using 2G12 (1 µg/mL) for detection. **(B)-(E)** Representative of $n \geq 3$ independent experiments. (**F**) Mass spectrometry spectra of singly (left panel) and doubly charged ions extracted using driftscope. Abundant glycan structures and larger complex glycans are labelled. The m/z=1873 is a hybrid lacking a mannose on 6 arm, but both isomers are present. (**F**) Thermal stability assessment of ConSOSL.UFO.664 (red) using nanoDSF, Tm is indicated. BG505 SOSIP.664 (black) trimer is also plotted for comparison. (**H**) Thermostability of ConSOSL.UFO.664 at +4ºC and (**I**) +37ºC over 8 weeks, representative of $n = 1$ experiment.

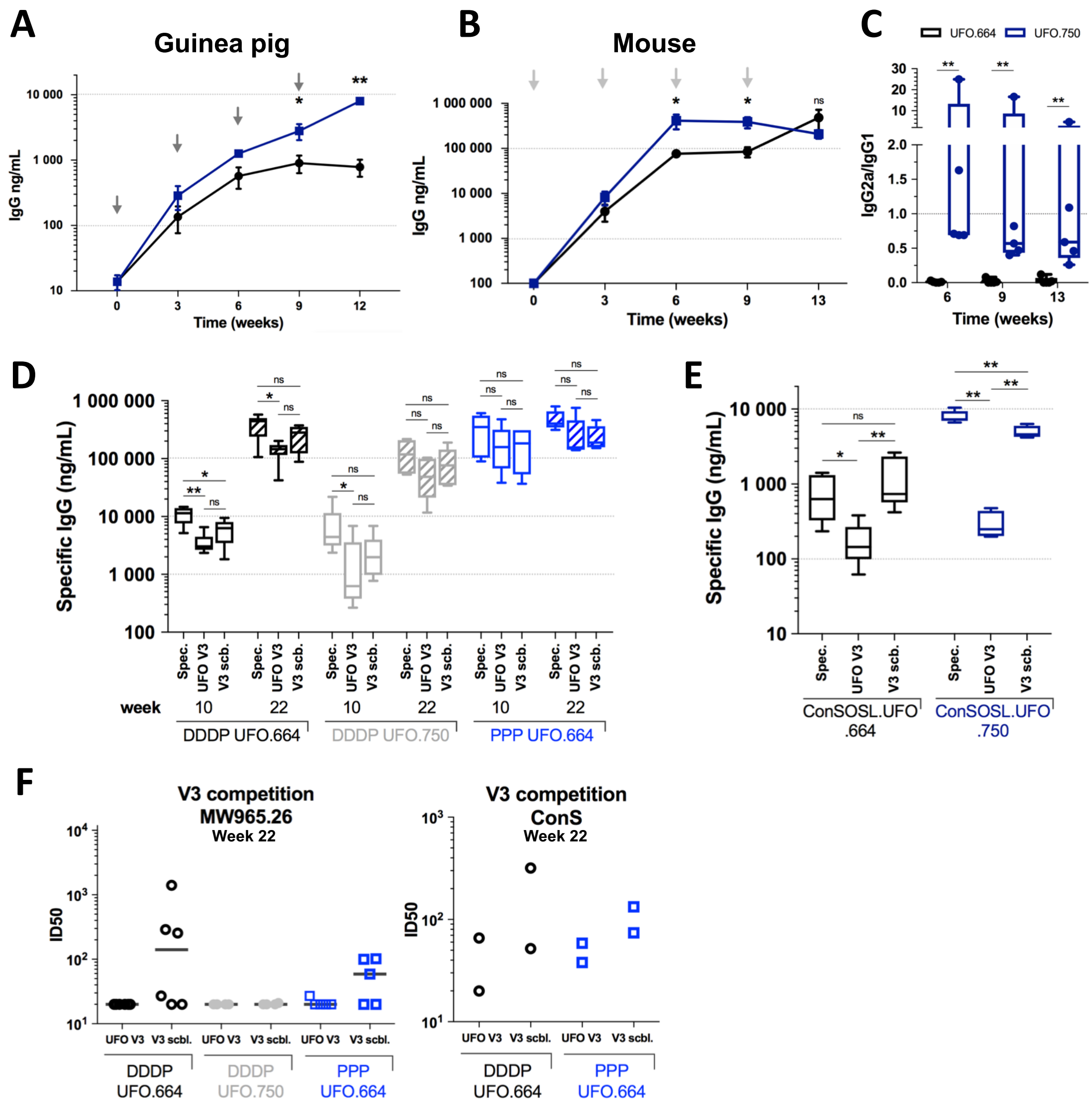

Figure S7. Immunogenicity of ConSOSL.UFO design in Guinea pigs and mice and V3 specific response in rabbits. Related to Figure 7. (**A**) Antigen specific serum IgG binding for DNA immunized Guinea pigs and (**B**) mice with ConSOSL.UFO.750 plasmid (blue) and ConSOSL.UFO.664 plasmid (black). Binding assessed by 9E10 capture ELISA for Guinea pig's sera and by directly coated ConSOSL.UFO.664 trimers for mouse sera. Arrows indicate immunization with DNA IM+EP. For (**A**) time points 0, 3 and 6, n = 6 animals per group, then for time points 9 and 12, n = 5. Mice, n = 5 per group. Error bars represent mean ± SEM values. (**C**) Mouse IgG1 and IgG2a antigen specific responses were assessed by ELISA and the IgG1/IgG2a ratio is shown here. (**D**) Rabbit serum samples from week 10 and 22 and (**E**) Guinea pig's serum samples from week 12 were incubated 30min prior loading onto the ELISA plates with 10ug/mL ConSOSL.UFO V3 cys-cyclised (UFO V3) peptide or MN.3 V3 scrambled linear (V3 scbl.) peptide as a control and the specific binding to ConSOSL.UFO.664 Myc-HIS captured antigen measured. The total specific response is plotted and referred as 'Spec.'. (**F**) Neutralization of HIV-1 MW965.26 and ConS pseudoviruses in TZM-bl assay by rabbit neutralizer sera from week 22 in competition with UFO V3 and V3 scbl. peptides. Data plotted as ID50 values (serum dilution that inhibits by 50% infectivity). Unpaired t-test, p values: *<0.05, **<0.01.

```
BG505_gp160_DQ208458_1    MRVMGIQRNCQHLFRWGTMILGMIIICSAAENLWVTVYYGVPVWKDAETTLFCASDAKAY     60
ConS_gp160                MRVRGIQRNCQHLWRWGTLILGMLMICSAAENLWVTVYYGVPVWKEANTTLFCASDAKAY     60
ConSOSIP_712              ---------MDRAKLLLLLLLLLLLPQAQAVENLWVTVYYGVPVWKEAETTLFCASDAKAY     51
ConSOSVD_712              ---------MDRAKLLLLLLLLLLLPQAQAVENLWVTVYYGVPVWKEAETTLFCASDAKAY     51
ConSL_712                 ---------MDRAKLLLLLLLLLLLPQAQAVENLWVTVYYGVPVWKEAETTLFCASDAKAY     51
ConSLVD_712               ---------MDRAKLLLLLLLLLLLPQAQAVENLWVTVYYGVPVWKEAETTLFCASDAKAY     51
ConSLIP_712               ---------MDRAKLLLLLLLLLLLPQAQAVENLWVTVYYGVPVWKEAETTLFCASDAKAY     51
ConSLwIP_712              ---------MDRAKLLLLLLLLLLLPQAQAVENLWVTVYYGVPVWKEAETTLFCASDAKAY     51
ConSLIPv2_750             ---------MDRAKLLLLLLLLLLLPQAQAVENLWVTVYYGVPVWKDAETTLFCASDAKAY     51
ConSOSLIP_750             ---------MDRAKLLLLLLLLLLLPQAQAVENLWVTVYYGVPVWKDAETTLFCASDAKAY     51
ConSOSL_UFO_750           ---------MDRAKLLLLLLLLLLLPQAQAVENLWVTVYYGVPVWKDAETTLFCASDAKAY     51
ConSOSL_UFO_664           ---------MDRAKLLLLLLLLLLLPQAQAVENLWVTVYYGVPVWKDAETTLFCASDAKAY     51
ConSv2_gp160              ---------MDRAKLLLLLLLLLLLPQAQAVENLWVTVYYGVPVWKDAETTLFCASDAKAY     51
                          |----   -Signal-Sequence  --||-----------------------------
                          |           |               |         |         |         |
                          1           10              30        40        50        60

BG505_gp160_DQ208458_1    ETEKHNVWATHACVPTDPNPQEIHLENVTEEFNMWKNNMVEQMHTDIISLWDQSLKPCVK     120
ConS_gp160                DTEVHNVWATHACVPTDPNPQEIVLENVTENFNMWKNNMVEQMHEDIISLWDQSLKPCVK     120
ConSOSIP_712              DTEKHNVWATHACVPTDPNPQEIHLENVTEEFNMWKNNMVEQMHTDIISLWDQSLKPCVK     111
ConSOSVD_712              DTEKHNVWATHACVPTDPNPQEIHLENVTEEFNMWKNNMVEQMHTDIISLWDQSLKPCVK     111
ConSL_712                 DTEKHNVWATHACVPTDPNPQEIHLENVTEEFNMWKNNMVEQMHTDIISLWDQSLKPCVK     111
ConSLVD_712               DTEKHNVWATHACVPTDPNPQEIHLENVTEEFNMWKNNMVEQMHTDIISLWDQSLKPCVK     111
ConSLIP_712               DTEKHNVWATHACVPTDPNPQEIHLENVTEEFNMWKNNMVEQMHTDIISLWDQSLKPCVK     111
ConSLwIP_712              DTEKHNVWATHACVPTDPNPQEIHLENVTEEFNMWKNNMVEQMHTDIISLWDQSLKPCVK     111
ConSLIPv2_750             DTEVRNVWATHACVPTDPNPQEIVLENVTENFNMWKNNMVEQMHTDIISLWDQSLKPCVK     111
ConSOSLIP_750             DTEVRNVWATHACVPTDPNPQEIVLENVTENFNMWKNNMVEQMHTDIISLWDQSLKPCVK     111
ConSOSL_UFO_750           DTEVRNVWATHACVPTDPNPQEIVLENVTENFNMWKNNMVEQMHTDIISLWDQSLKPCVK     111
ConSOSL_UFO_664           DTEVRNVWATHACVPTDPNPQEIVLENVTENFNMWKNNMVEQMHTDIISLWDQSLKPCVK     111
ConSv2_gp160              DTEVRNVWATHACVPTDPNPQEIVLENVTENFNMWKNNMVEQMHTDIISLWDQSLKPCVK     111
                          --------------------C1-Domain---------------------------||--
                                  |         |         |         |         |         |
                                  70        80        90        100       110       120

BG505_gp160_DQ208458_1    LTPLCVTLQCTNVTNNI---TDDMRGELKNCSFNMTTELRDKKQKVYSLFYRLDVVQINE     177
ConS_gp160                LTPLCVTLNCTNVNVTNTTNNTEEKGEIKNCSFNITTEIRDKKQKVYALFYRLDVVPIDD     180
ConSOSIP_712              LTPLCVTLNCTNVNVTNTTNNTEEKGEIKNCSFNMTTELRDKKQKVYSLFYRLDVVPIDD     171
ConSOSVD_712              LTPLCVTLNCTNVNVTNTTNNTEEKGEIKNCSFNMTTELRDKKQKVYSLFYRLDVVPIDD     171
ConSL_712                 LTPLCVTLNCTNVNVTNTTNNTEEKGEIKNCSFNMTTELRDKKQKVYSLFYRLDVVPIDD     171
ConSLVD_712               LTPLCVTLNCTNVNVTNTTNNTEEKGEIKNCSFNMTTELRDKKQKVYSLFYRLDVVPIDD     171
ConSLIP_712               LTPLCVTLNCTNVNVTNTTNNTEEKGEIKNCSFNMTTELRDKKQKVYSLFYRLDVVPIDD     171
ConSLwIP_712              LTPLCVTLNCTNVNVTNTTNNTEEKGEIKNCSFNMTTELRDKKQKVYSLFYRLDVVPIDD     171
ConSLIPv2_750             LTPLCVTLNCTNVNVTNTTNNTEEKGEIKNCSFNITTELRDKKKKVYALFYRLDVVPIDD     171
ConSOSLIP_750             LTPLCVTLNCTNVNVTNTTNNTEEKGEIKNCSFNITTELRDKKKKVYALFYRLDVVPIDD     171
ConSOSL_UFO_750           LTPLCVTLNCTNVNVTNTTNNTEEKGEIKNCSFNITTELRDKKKKVYALFYRLDVVPIDD     171
ConSOSL_UFO_664           LTPLCVTLNCTNVNVTNTTNNTEEKGEIKNCSFNITTELRDKKKKVYALFYRLDVVPIDD     171
ConSv2_gp160              LTPLCVTLNCTNVNVTNTTNNTEEKGEIKNCSFNITTELRDKKKKVYALFYRLDVVPIDD     171
                          ------V1-Domain------ --------||------------V2-Domain-------
                                  |         |    |         |         |         |
                                  130       140  150       160       170       180

BG505_gp160_DQ208458_1    NQGNRSNNSNKEYRLINCNTSAITQACPKVSFEPIPIHYCAPAGFAILKCKDKKFNGTGP     237
ConS_gp160                NNN-----NSSNYRLINCNTSAITQACPKVSFEPIPIHYCAPAGFAILKCNDKKFNGTGP     235
ConSOSIP_712              NNN-----NSSNYRLINCNTSAITQACPKVSFEPIPIHYCAPAGFAILKCNDKKFNGTGP     226
ConSOSVD_712              NNN-----NSSNYRLINCNTSACTQACPKVSFEPIPIHYCAPAGFAILKCNDKKFNGTGP     226
ConSL_712                 NNN-----NSSNYRLINCNTSAITQACPKVSFEPIPIHYCAPAGFAILKCNDKKFNGTGP     226
ConSLVD_712               NNN-----NSSNYRLINCNTSACTQACPKVSFEPIPIHYCAPAGFAILKCNDKKFNGTGP     226
ConSLIP_712               NNN-----NSSNYRLINCNTSAITQACPKVSFEPIPIHYCAPAGFAILKCNDKKFNGTGP     226
ConSLwIP_712              NNN-----NSSNYRLINCNTSAITQACPKVSFEPIPIHYCAPAGFAILKCNDKKFNGTGP     226
ConSLIPv2_750             NNN-----NSSNYRLINCNTSAITQACPKVSFEPIPIHYCAPAGFAILKCNDKKFNGTGP     226
ConSOSLIP_750             NNN-----NSSNYRLINCNTSAITQACPKVSFEPIPIHYCAPAGFAILKCNDKKFNGTGP     226
ConSOSL_UFO_750           NNN-----NSSNYRLINCNTSAITQACPKVSFEPIPIHYCAPAGFAILKCNDKKFNGTGP     226
ConSOSL_UFO_664           NNN-----NSSNYRLINCNTSAITQACPKVSFEPIPIHYCAPAGFAILKCNDKKFNGTGP     226
ConSv2_gp160              NNN-----NSSNYRLINCNTSAITQACPKVSFEPIPIHYCAPAGFAILKCNDKKFNGTGP     226
                                  ------------------||--------------C2-Domain---------
                                     |         |         |         |         |
                                     190       200       210       220       230
```

```
BG505_gp160_DQ208458_1       CPSVSTVQCTHGIKPVVSTQLLLNGSLAEEEVMIRSENITNNAKNILVQFNTPVQINCTR     297
ConS_gp160                   CKNVSTVQCTHGIKPVVSTQLLLNGSLAEEEIIIRSENITNNAKTIIVQLNESVEINCTR     295
ConSOSIP_712                 CKNVSTVQCTHGIKPVVSTQLLLNGSLAEEEVMIRSENITNNAKNILVQFNTPVQINCTR     286
ConSOSVD_712                 CKNVSTVQCTHGIKPVVSTQLLLNGSLAEEEVMIRSENITNNAKNILVQFNTPVQINCTR     286
ConSL_712                    CKNVSTVQCTHGIKPVVSTQLLLNGSLAEEEVMIRSENITNNAKNILVQFNTPVQINCTR     286
ConSLVD_712                  CKNVSTVQCTHGIKPVVSTQLLLNGSLAEEEVMIRSENITNNAKNILVQFNTPVQINCTR     286
ConSLIP_712                  CKNVSTVQCTHGIKPVVSTQLLLNGSLAEEEVMIRSENITNNAKNILVQFNTPVQINCTR     286
ConSLwIP_712                 CKNVSTVQCTHGIKPVVSTQLLLNGSLAEEEVMIRSENITNNAKNILVQFNTPVQINCTR     286
ConSLIPv2_750                CKNVSTVQCTHGIKPVVSTQLLLNGSLAEEEIIIRSENITNNAKTIIVQLNESVEINCTR     286
ConSOSLIP_750                CKNVSTVQCTHGIKPVVSTQLLLNGSLAEEEIIIRSENITNNAKTIIVQLNESVEINCTR     286
ConSOSL_UFO_750              CKNVSTVQCTHGIKPVVSTQLLLNGSLAEEEIIIRSENITNNAKTIIVQLNESVEINCTR     286
ConSOSL_UFO_664              CKNVSTVQCTHGIKPVVSTQLLLNGSLAEEEIIIRSENITNNAKTIIVQLNESVEINCTR     286
ConSv2_gp160                 CKNVSTVQCTHGIKPVVSTQLLLNGSLAEEEIIIRSENITNNAKTIIVQLNESVEINCTR     286
                             ----------------------------C2-Domain--------------------||-
                              |         |         |         |         |         |
                             240       250       260       270       280       290

BG505_gp160_DQ208458_1       PNNNTRKSIRIGPGQAFYATGDIIGDIRQAHCTVSKATWNETLGKVVKQLRKHFGNNTII     357
ConS_gp160                   PNNNTRKSIRIGPGQAFYATGDIIGDIRQAHCNISGTKWNKTLQQVAKKLREHFNNKTI-     354
ConSOSIP_712                 PNNNTRKSIRIGPGQAFYATGDIIGDIRQAHCNVSKATWNETLGKVVKQLRKHFGNNTII     346
ConSOSVD_712                 PNNNTRKSIRIGPGQAFYATGDIIGDIRQAHCNVSKATWNETLGKVVKQLRKHFGNNTII     346
ConSL_712                    PNNNTRKSIRIGPGQAFYATGDIIGDIRQAHCNVSKATWNETLGKVVKQLRKHFGNNTII     346
ConSLVD_712                  PNNNTRKSIRIGPGQAFYATGDIIGDIRQAHCNVSKATWNETLGKVVKQLRKHFGNNTII     346
ConSLIP_712                  PNNNTRKSIRIGPGQAFYATGDIIGDIRQAHCNVSKATWNETLGKVVKQLRKHFGNNTII     346
ConSLwIP_712                 PNNNTRKSIRIGPGQAFYATGDIIGDIRQAHCNVSKATWNETLGKVVKQLRKHFGNNTII     346
ConSLIPv2_750                PNNNTRKSIRIGPGQWFYATGDIIGDIRQAHCNISGTKWNKTLQQVVKKLREHFNNKTI-     345
ConSOSLIP_750                PNNNTRKSIRIGPGQWFYATGDIIGDIRQAHCNISGTKWNKTLQQVVKKLREHFNNKTI-     345
ConSOSL_UFO_750              PNNNTRKSIRIGPGQWFYATGDIIGDIRQAHCNISGTKWNKTLQQVVKKLREHFNNKTI-     345
ConSOSL_UFO_664              PNNNTRKSIRIGPGQWFYATGDIIGDIRQAHCNISGTKWNKTLQQVVKKLREHFNNKTI-     345
ConSv2_gp160                 PNNNTRKSIRIGPGQWFYATGDIIGDIRQAHCNISGTKWNKTLQQVVKKLREHFNNKTI-     345
                             -----------------V3-Loop- ----||--------------C3-Domain-----
                              |       ||        |          |         |         |
                             300    308/311    320        330       340       350

BG505_gp160_DQ208458_1       RFANSSGGDLEVTTHSFNCGGEFFYCNTSGLFNSTWISNTSVQGSNSTGSNDSITLPCRI     417
ConS_gp160                   IFKPSSGGDLEITTHSFNCRGEFFYCNTSGLFNSTWIGNGTKN---NNNTNDTITLPCRI     411
ConSOSIP_712                 RFANSSGGDLEVTTHSFNCGGEFFYCNTSGLFNSTWIGNGTKN---NNNTNDTITLPCRI     403
ConSOSVD_712                 RFANSSGGDLEVTTHSFNCGGEFFYCNTSGLFNSTWIGNGTKN---NNNTNDTITLPCRI     403
ConSL_712                    RFANSSGGDLEVTTHSFNCGGEFFYCNTSGLFNSTWIGNGTKN---NNNTNDTITLPCRI     403
ConSLVD_712                  RFANSSGGDLEVTTHSFNCGGEFFYCNTSGLFNSTWIGNGTKN---NNNTNDTITLPCRI     403
ConSLIP_712                  RFANSSGGDLEVTTHSFNCGGEFFYCNTSGLFNSTWIGNGTKN---NNNTNDTITLPCRI     403
ConSLwIP_712                 RFANSSGGDLEVTTHSFNCGGEFFYCNTSGLFNSTWIGNGTKN---NNNTNDTITLPCRI     403
ConSLIPv2_750                IFNPSSGGDLEITTHSFNCGGEFFYCNTSGLFNSTWIGNGTKN---NNNTNDTITLPCRI     402
ConSOSLIP_750                IFNPSSGGDLEITTHSFNCGGEFFYCNTSGLFNSTWIGNGTKN---NNNTNDTITLPCRI     402
ConSOSL_UFO_750              IFNPSSGGDLEITTHSFNCGGEFFYCNTSGLFNSTWIGNGTKN---NNNTNDTITLPCRI     402
ConSOSL_UFO_664              IFNPSSGGDLEITTHSFNCGGEFFYCNTSGLFNSTWIGNGTKN---NNNTNDTITLPCRI     402
ConSv2_gp160                 IFNPSSGGDLEITTHSFNCGGEFFYCNTSGLFNSTWIGNGTKN---NNNTNDTITLPCRI     402
                             -------------------------||----V4-Loop-------------------||--
                             |         |         |         |       ||         |         |
                            360       370       380       390    399/401     410       420

BG505_gp160_DQ208458_1       KQIINMWQRIGQAMYAPPIQGVIRCVSNITGLILTRDGGSTNS-TTETFRPGGGDMRDNW     476
ConS_gp160                   KQIINMWQGVGQAMYAPPIEGKITCKSNITGLLLTRDGGNNNTNETEIFRPGGGDMRDNW     471
ConSOSIP_712                 KQIINMWQRIGQAMYAPPIQPVIRCVSNITGLILTRDGGSTNS-TTETFRPGGGDMRDNW     462
ConSOSVD_712                 KQIINMWQRIGQCMYAPPIQCVIRCVSNITGLILTRDGGSTNS-TTETFRPGGGDMRDNW     462
ConSL_712                    KQIINMWQRIGQPMYAPPIQGVIRCVSNITGLILTRDGGSTNS-TTETFRPGGGDMRDNW     462
ConSLVD_712                  KQIINMWQRIGQCMYAPPIQGVIRCVSNITGLILTRDGGSTNS-TTETFRPGGGDMRDNW     462
ConSLIP_712                  KQIINMWQRIGQPMYAPPIQGVIRCVSNITGLILTRDGGSTNS-TTETFRPGGGDMRDNW     462
ConSLwIP_712                 KQIINMWQRIGQPMYAPPIQGVIRCVSNITGLILTRDGGSTNS-TTETFRPGGGDMRDNW     462
ConSLIPv2_750                KQIINMWQRVGQPMYAPPIQGKIRCVSNITGLLLTRDGGNNNTNETETFRPGGGDMRDNW     462
ConSOSLIP_750                KQIINMWQRVGQPMYAPPIQGKIRCVSNITGLLLTRDGGNNNTNETETFRPGGGDMRDNW     462
ConSOSL_UFO_750              KQIINMWQRVGQPMYAPPIQGKIRCVSNITGLLLTRDGGNNNTNETETFRPGGGDMRDNW     462
ConSOSL_UFO_664              KQIINMWQRVGQPMYAPPIQGKIRCVSNITGLLLTRDGGNNNTNETETFRPGGGDMRDNW     462
ConSv2_gp160                 KQIINMWQRVGQAMYAPPIQGKIRCVSNITGLLLTRDGGNNNTNETETFRPGGGDMRDNW     462
                             ---------------C4-Domain-------------||V5Lp||-b24--||----C5-
                                      |         |         |         |          |        |
                                     430       440       450       460        470      479
```

```
BG505_gp160_DQ208458_1      RSELYKYKVVKIEPLGVAPTRAKRRVVGREKR----------AVGIGAVFLGFLGAAGST     526
ConS_gp160                  RSELYKYKVVKIEPLGVAPTKAKRRVVEREKR----------AVGIGAVFLGFLGAAGST     521
ConSOSIP_712                RSELYKYKVVKIEPLGVAPTRCKRRVVGRRRR--------RRAVGIGAVFLGFLGAAGST     514
ConSOSVD_712                RSELYKYKVVKIEPLGVAPTRCKRRVVGRRRR--------RRAVGIGAVFLGFLGAAGST     514
ConSL_712                   RSELYKYKVVKIEPLGVAPTKAKRRVVESEKRSGGGGSGGGGAVGIGAVFLGFLGAAGST     522
ConSLVD_712                 RSELYKYKVVKIEPLGVAPTKAKRRVVESEKRSGGGGSGGGGAVGIGAVFLGFLGAAGST     522
ConSLIP_712                 RSELYKYKVVKIEPLGVAPTKAKRRVVESEKRSGGGGSGGGGAVGIGAVFLGFLGAAGST     522
ConSLwIP_712                RSELYKYKVVKIEPLGVAPTKAKRRVVESEKRGGGGSGGGGSAVGIGAVFLGFLGAAGST     522
ConSLIPv2_750               RSELYKYKVVKIEPLGVAPTKAKRRVVESEKRGGGGSGGGGSAVGIGAVFLGFLGAAGST     522
ConSOSLIP_750               RSELYKYKVVKIEPLGVAPTRCKRRVVE----GGGGSGGGGSAVGIGAVFLGFLGAAGST     518
ConSOSL_UFO_750             RSELYKYKVVKIEPLGVAPTRCKRRVVE----GGGGSGGGGSAVGIGAVFLGFLGAAGST     518
ConSOSL_UFO_664             RSELYKYKVVKIEPLGVAPTRCKRRVVE----GGGGSGGGGSAVGIGAVFLGFLGAAGST     518
ConSv2_gp160                RSELYKYKVVKIEPLGVAPTRAKRRVVEREKR----------AVGIGAVFLGFLGAAGST     512
                            ---------C5-Domain------------|            |-----------HR-1--
                            |         |         |         |            |       |
                           480       490       500       510                  520

BG505_gp160_DQ208458_1      MGAASMTLTVQARNLLSGIVQQQSNLLRAIEAQQHLLKLTVWGIKQLQARVLAVERYLRD     586
ConS_gp160                  MGAASITLTVQARQLLSGIVQQQSNLLRAIEAQQHLLQLTVWGIKQLQARVLAVERYLKD     581
ConSOSIP_712                MGAASMTLTVQARNLLSGIVQQQSNLLRAPEAQQHLLKLTVWGIKQLQARVLAVERYLRD     574
ConSOSVD_712                MGAASMTLTVQARNLLSGIVQQQSNLLRAIEAQQHLLKLTDWGIKQLQARVLAVERYLRD     574
ConSL_712                   MGAASMTLTVQARNLLSGIVQQQSNLLRAIEAQQHLLKLTVWGIKQLQARVLAVERYLRD     582
ConSLVD_712                 MGAASMTLTVQARNLLSGIVQQQSNLLRAIEAQQHLLKLTDWGIKQLQARVLAVERYLRD     582
ConSLIP_712                 MGAASMTLTVQARNLLSGIVQQQSNLLRAPEAQQHLLKLTVWGIKQLQARVLAVERYLRD     582
ConSLwIP_712                MGAASMTLTVQARNLLSGIVQQQSNLLRAPEAQQHLLKLTVWGIKQLQARVLAVERYLRD     582
ConSLIPv2_750               MGAASMTLTVQARNLLSGIVQQQSNLLRAPEAQQHLLQLTVWGIKQLQARVLAVERYLRD     582
ConSOSLIP_750               MGAASMTLTVQARNLLSGIVQQQSNLLRAPEAQQHLLQLTVWGIKQLQARVLAVERYLRD     578
ConSOSL_UFO_750             MGAASMTLTVQARNLLSGGSGSG-------------SGSTVWGIKQLQARVLAVERYLRD     565
ConSOSL_UFO_664             MGAASMTLTVQARNLLSGGSGSG-------------SGSTVWGIKQLQARVLAVERYLRD     565
ConSv2_gp160                MGAASMTLTVQARNLLSGIVQQQSNLLRAIEAQQHLLQLTVWGIKQLQARVLAVERYLRD     572
                            ------------------Heptad-Repeat-Helices-1-------------------
                            |         |         |         |         |         |        |
                           530       540       550       560       570       580      589

BG505_gp160_DQ208458_1      QQLLGIWGCSGKLICTTNVPWNSSWSNRNLSEIWDNMTWLQWDKEISNYTQIIYGLLEES     646
ConS_gp160                  QQLLGIWGCSGKLICTTTVPWNSSWSNKSQDEIWDNMTWMEWEREINNYTDIIYSLIEES     641
ConSOSIP_712                QQLLGIWGCSGKLICCTNVPWNSSWSNRNLSEIWDNMTWLQWDKEISNYTQIIYGLLEES     634
ConSOSVD_712                QQLLGIWGCSGKLICCTNVPWNSSWSNRNLSEIWDNMTWLQWDKEISNYTQIIYGLLEES     634
ConSL_712                   QQLLGIWGCSGKLICTTNVPWNSSWSNRNLSEIWDNMTWLQWDKEISNYTQIIYGLLEES     642
ConSLVD_712                 QQLLGIWGCSGKLICTTNVPWNSSWSNRNLSEIWDNMTWLQWDKEISNYTQIIYGLLEES     642
ConSLIP_712                 QQLLGIWGCSGKLICTTNVPWNSSWSNRNLSEIWDNMTWLQWDKEISNYTQIIYGLLEES     642
ConSLwIP_712                QQLLGIWGCSGKLICTTNVPWNSSWSNRNLSEIWDNMTWLQWDKEISNYTQIIYGLLEES     642
ConSLIPv2_750               QQLLGIWGCSGKLICTTNVPWNSSWSNKSQDEIWDNMTWMEWDKEINNYTDIIYSLIEES     642
ConSOSLIP_750               QQLLGIWGCSGKLICCTNVPWNSSWSNKSQDEIWDNMTWMEWDKEINNYTDIIYSLIEES     638
ConSOSL_UFO_750             QQLLGIWGCSGKLICCTNVPWNSSWSNKSQDEIWDNMTWMEWDKEINNYTDIIYSLIEES     625
ConSOSL_UFO_664             QQLLGIWGCSGKLICCTNVPWNSSWSNKSQDEIWDNMTWMEWDKEINNYTDIIYSLIEES     625
ConSv2_gp160                QQLLGIWGCSGKLICTTNVPWNSSWSNKSQDEIWDNMTWMEWDKEINNYTDIIYSLIEES     632
                            -------||CC-Lp||---------------Heptad-Repeat-Helices-2------
                            |         |         |         |         |         |        |
                           590       600       610       620       630       640      649

BG505_gp160_DQ208458_1      QNQQEKNEQDLLALDKWASLWNWFDISNWLWYIKIFIMIVGGLIGLRIVFAVLSVIHRVR     706
ConS_gp160                  QNQQEKNEQELLALDKWASLWNWFDITNWLWYIKIFIMIVGGLIGLRIVFAVLSIVNRVR     701
ConSOSIP_712                QNQQEKNEQDLLALDKWASLWNWFDISNWLWYIKIFIMIVGGLIGLRIVFAVLSVIHRVR     694
ConSOSVD_712                QNQQEKNEQDLLALDKWASLWNWFDISNWLWYIKIFIMIVGGLIGLRIVFAVLSVIHRVR     694
ConSL_712                   QNQQEKNEQDLLALDKWASLWNWFDISNWLWYIKIFIMIVGGLIGLRIVFAVLSVIHRVR     702
ConSLVD_712                 QNQQEKNEQDLLALDKWASLWNWFDISNWLWYIKIFIMIVGGLIGLRIVFAVLSVIHRVR     702
ConSLIP_712                 QNQQEKNEQDLLALDKWASLWNWFDISNWLWYIKIFIMIVGGLIGLRIVFAVLSVIHRVR     702
ConSLwIP_712                QNQQEKNEQDLLALDKWASLWNWFDISNWLWYIKIFIMIVGGLIGLRIVFAVLSVIHRVR     702
ConSLIPv2_750               QNQQEKNEQDLLALDKWASLWNWFDITNWLWYIKIFIMIVGGLIGLRIVFAVLSIVNRVR     702
ConSOSLIP_750               QNQQEKNEQDLLALDKWASLWNWFDITNWLWYIKIFIMIVGGLIGLRIVFAVLSIVNRVR     698
ConSOSL_UFO_750             QNQQEKNEQDLLALDKWASLWNWFDITNWLWYIKIFIMIVGGLIGLRIVFAVLSIVNRVR     685
ConSOSL_UFO_664             QNQQEKNEQDLLALD---------------------------------------------     640
ConSv2_gp160                QNQQEKNEQDLLALDKWASLWNWFDITNWLWYIKIFIMIVGGLIGLRIVFAVLSIVNRVR     692
                            -----------||--------MPER--------||------TM-Domain-----||---
                            |         |         |         |         |         |        |
                           650       660       670       680       690       700      709
```

```
BG505_gp160_DQ208458_1      QGYSPLSFQTHTPNPRGLDRPERIEEEDGEQDRGRSTRLVSGFLALAWDDLRSLCLFCYH    766
ConS_gp160                  QGYSPLSFQTLIPNPRGPDRPEGIEEEGGEQDRDRSIRLVNGFLALAWDDLRSLCLFSYH    761
ConSOSIP_712                QG----------------------------------------------------------    696
ConSOSVD_712                QG----------------------------------------------------------    696
ConSL_712                   QG----------------------------------------------------------    704
ConSLVD_712                 QG----------------------------------------------------------    704
ConSLIP_712                 QG----------------------------------------------------------    704
ConSLwIP_712                QG----------------------------------------------------------    704
ConSLIPv2_750               QGISPLSFQTLIPNPRGPDRPEGIEEEGGEQDRDRSIRLVN-------------------    743
ConSOSLIP_750               QGISPLSFQTLIPNPRGPDRPEGIEEEGGEQDRDRSIRLVN-------------------    739
ConSOSL_UFO_750             QGISPLSFQTLIPNPRGPDRPEGIEEEGGEQDRDRSIRLVN-------------------    726
ConSOSL_UFO_664             ------------------------------------------------------------    640
ConSv2_gp160                QGYSPLSFQTLIPNPRGPDRPEGIEEEGGEQDRDRSIRLVNGFLALAWDDLRSLCLFSYH    752
                            ------------CT-Domains----------------CT-Domains------------
                            |         |         |         |         |         |        |
                           710       720       730       740       750       760      769

BG505_gp160_DQ208458_1      RLRDFILIAARIVELLGHSSLKGLRLGWEGLKYLWNLLAYWGRELKISAINLFDTIAIAV    826
ConS_gp160                  RLRDFILIAARTVELLGR---KGLRRGWEALKYLWNLLQYWGQELKNSAISLLDTTAIAV    818
ConSOSIP_712                ------------------------------------------------------------    696
ConSOSVD_712                ------------------------------------------------------------    696
ConSL_712                   ------------------------------------------------------------    704
ConSLVD_712                 ------------------------------------------------------------    704
ConSLIP_712                 ------------------------------------------------------------    704
ConSLwIP_712                ------------------------------------------------------------    704
ConSLIPv2_750               ------------------------------------------------------------    743
ConSOSLIP_750               ------------------------------------------------------------    739
ConSOSL_UFO_750             ------------------------------------------------------------    726
ConSOSL_UFO_664             ------------------------------------------------------------    640
ConSv2_gp160                RLRDFILIAARTVELLGR---KGLRRGWEALKYLWNLLQYWGQELKNSAISLLDTTAIAV    809
                            ---------CT-------       ----------------CT-----------------
                            |         |                |         |         |         |
                           770       780              790       800       810       820

BG505_gp160_DQ208458_1      AEWTDRVIEIGQRLCRAFLHIPRRIRQGLERALL    860
ConS_gp160                  AEGTDRVIEVVQRACRAILNIPRRIRQGLERALL    852
ConSOSIP_712                ----------------------------------    696
ConSOSVD_712                ----------------------------------    696
ConSL_712                   ----------------------------------    704
ConSLVD_712                 ----------------------------------    704
ConSLIP_712                 ----------------------------------    704
ConSLwIP_712                ----------------------------------    704
ConSLIPv2_750               ----------------------------------    743
ConSOSLIP_750               ----------------------------------    739
ConSOSL_UFO_750             ----------------------------------    726
ConSOSL_UFO_664             ----------------------------------    640
ConSv2_gp160                AEGTDRVIEVVQRACRAILNIPRRIRQGLERALL    843
                            -----------------CT---------------|
                                   |         |         |     |
                                  830       840       850   856
```

B. ConSOSL.UFO.750

```
MDRAKLLLLLLLLLLPQAQAVENLWVTVYYGVPVWKDAETTLFCASDAKAYDTEVRNVWATHACVPTDPNPQEIVLENVTENFNMWKNNMV
EQMHTDIISLWDQSLKPCVKLTPLCVTLNCTNVNVTNTTNNTEEKGEIKNCSFNITTELRDKKKKVYALFYRLDVVPIDDNNNNSSNYRLI
NCNTSAITQACPKVSFEPIPIHYCAPAGFAILKCNDKKFNGTGPCKNVSTVQCTHGIKPVVSTQLLLNGSLAEEEIIIRSENITNNAKTII
VQLNESVEINCTRPNNNTRKSIRIGPGQWFYATGDIIGDIRQAHCNISGTKWNKTLQQVVKKLREHFNNKTIIFNPSSGGDLEITTHSFNC
GGEFFYCNTSGLFNSTWIGNGTKNNNNTNDTITLPCRIKQIINMWQRVGQPMYAPPIQGKIRCVSNITGLLLTRDGGNNNTNETETFRPGG
GDMRDNWRSELYKYKVVKIEPLGVAPTRCKRRVVEGGGGSGGGGSAVGIGAVFLGFLGAAGSTMGAASMTLTVQARNLLSGGSGSGSGSTV
WGIKQLQARVLAVERYLRDQQLLGIWGCSGKLICCTNVPWNSSWSNKSQDEIWDNMTWMEWDKEINNYTDIIYSLIEESQNQQEKNEQDLL
ALDKWASLWNWFDITNWLWYIKIFIMIVGGLIGLRIVFAVLSIVNRVRQGISPLSFQTLIPNPRGPDRPEGIEEEGGEQDRDRSIRLVN*
```

Data S1. Sequence alignment and numbering using HXB2 numbering reference. Related to Figure 1. (A) Sequences were aligned using Clustal Omega online tool (EMBL-EBI). HXB2 numbering was attributed according to the lanl database using the HXB2 K03455 reference sequence (https://www.hiv.lanl.gov/content/sequence/HIV/REVIEWS/HXB2.html). Highlighted in grey are the amino acid introduced from BG505 (or specific to BG505), in yellow the amino acid reverted to the ConS gp160 sequence (or specific to ConS gp160) while pink highlighted amino acids indicate stabilization substitutions. The optimized cleavage site RRRRRR is coloured in blue and both cleavage site linker $(SG_4)_2$ and $(G_4S)_2$ are coloured in purple and pink respectively. (**B**) ConSOSL.UFO.750 amino acid sequence.
